# Targeting CXCR2 in prostate cancer cells can block CD47-SIRPα interaction and reverse M2 macrophage polarization in the TME

**DOI:** 10.1186/s12943-025-02436-1

**Published:** 2025-10-30

**Authors:** Yi Sun, Shangqing Ren, Wei Wen, Jun Jing, Xu Luo, Shuai Shao, Ruiqi Duan, Guohua Zeng, Ju Guo

**Affiliations:** 1 Department of Urology, Guangdong Provincial Key Laboratory of Urological Diseases, Guangdong Engineering Research Center of Urinary Minimally Invasive Surgery Robot and Intelligent Equipment, The First Affiliated Hospital of Guangzhou Medical University, 151 West Yanjiang Road, Guangzhou, Guangdong 510230 China; 2 Robotic Minimally Invasive Surgery Center, Sichuan Provincial People’s Hospital, School of Medicine, University of Electronic Science and Technology of China, Chengdu, Sichuan 610072 China; 3 Department of Urology, West China Tianfu Hospital, Sichuan University, Chengdu, Sichuan 610000 China; 4 Department of Rheumatology and Clinical Immunology, Shenshan Medical Center, Sun Yat-sen Memorial Hospital, Sun Yat-sen University, Shanwei, Guangdong 516621 China; 5 Department of Urology, West China School of Public Health and West China Fourth Hospital, Sichuan University, Chengdu, Sichuan 610000 China; 6 Department of Urology, The Second Xiangya Hospital, Key Laboratory of Diabetes Immunology (Central South University), Central South University, Ministry of Education, National Clinical Research Center for Metabolic Disease, Changsha, Hunan 410011 China; 7 Department of Obstetrics and Gynecology, Key Laboratory of Birth Defects and Related Diseases of Women and Children (Sichuan University), Ministry of Education, West China Second University Hospital, Sichuan University, Chengdu, Sichuan 610041 China; 8 Department of Urology, The First Affiliated Hospital of Nanchang University, Nanchang, Jiangxi 330008 China

**Keywords:** CD47, CD36, IL-8/CXCR2, Prostate cancer, Macrophage, Lipid metabolism

## Abstract

Neuroendocrine prostate cancer (NEPC) exhibits strong immune evasion and plays a critical role in regulating metabolic reprogramming within prostate cancer. High infiltration of CD36 + M2 tumour-associated macrophages (TAMs) and elevated CD47 expression in NEPC cells are often associated with poor progression-free survival in cancer patients. Understanding the mechanisms that regulate CD36 + M2 TAM infiltration and high CD47 expression in tumour cells within the prostate cancer tumour microenvironment (TME) is essential. Using cell models and two animal models, we discovered that the IL-8/CXCR2 pathway increases acetyl-CoA levels through metabolic reprogramming, which subsequently increases CD47 expression via acetylation. Moreover, this pathway modulates the membrane localization of CD47 by stimulating tumour cells to secrete palmitic acid and utilize palmitoylation mechanisms, thereby protecting tumour cells from macrophage-mediated phagocytosis. The IL-8/CXCR2 pathway also reshapes the metabolic microenvironment of the TME, increasing the infiltration of ω-3/6 polyunsaturated fatty acids (PUFAs) in the TME, which promotes the recruitment of CD36 + M2 TAMs. Preclinical studies in both NSG and C57BL/6 animal models demonstrated that targeting CXCR2 restored TAM phagocytic activity and significantly reduced tumour growth. These findings suggest that CXCR2-targeted immunotherapy holds promising therapeutic potential for prostate cancer and underscores its importance in translational medicine.

**Supplementary Information:**

The online version contains supplementary material available at 10.1186/s12943-025-02436-1.

## Introduction

Evasion of immune surveillance is a hallmark of therapy-resistant neuroendocrine prostate cancer (NEPC), yet current immune checkpoint inhibitors show minimal efficacy against this cancer [1]. This therapeutic gap may stem from the unique reliance of NEPC on lipid metabolism-driven immune suppression [2–5], in which the interleukin-8 (IL-8)/C-X-C motif chemokine receptor 2 (CXCR2) axis has emerged as a critical but underexplored node. Our previous work revealed that IL-8/CXCR2 activation orchestrates a triad of immunosuppressive events: (i) tumour-infiltrating regulatory T-cell (Treg) recruitment, (ii) CD8⁺ T-cell exhaustion, and (iii) M2-like tumour-associated macrophage (TAM) polarization [5]– [6], which are effects partially mediated by metabolic reprogramming. Moreover, recent evidence suggests that CXCR2 inhibition suppresses CD47 expression and reprograms macrophages away from the M2 phenotype, suggesting a direct link between CXCR2 signalling and phagocytic immune evasion [7]. Despite these findings, the mechanistic links between CXCR2 signalling, lipid metabolic remodelling, and CD47-mediated immune evasion remain poorly defined. CD47, also known as the “do not eat me” signal, is elevated in advanced prostate cancer and is modulated by inflammatory changes in the tumour microenvironment (TME) [8–11]. However, CD47 upregulation alone does not fully account for M2 macrophage polarization, highlighting the need to explore additional metabolic cues that govern this process. By integrating molecular, metabolic, and immunological analyses, we delineated a CXCR2-dependent immunosuppressive circuit involving CD47 palmitoylation, CD36–PPARγ signalling, and altered very long-chain polyunsaturated fatty acid (VLC-PUFA) metabolism. Given its upstream role in modulating lipid metabolism, immune checkpoint expression, and macrophage polarization, CXCR2 represents a therapeutically actionable node in NEPC. In this study, we hypothesized that IL-8/CXCR2 signalling drives immune evasion in NEPC by promoting CD47 expression and M2 TAM polarization through lipid metabolic reprogramming. Inhibition of IL-8/CXCR2 may disrupt multiple immunosuppressive pathways simultaneously, providing a rationale for therapeutic targeting of the CXCR2 axis in advanced prostate cancer.

## Results

### The IL-8/CXCR2 pathway in tumour cells regulates TAM infiltration

**Fig. 1:**
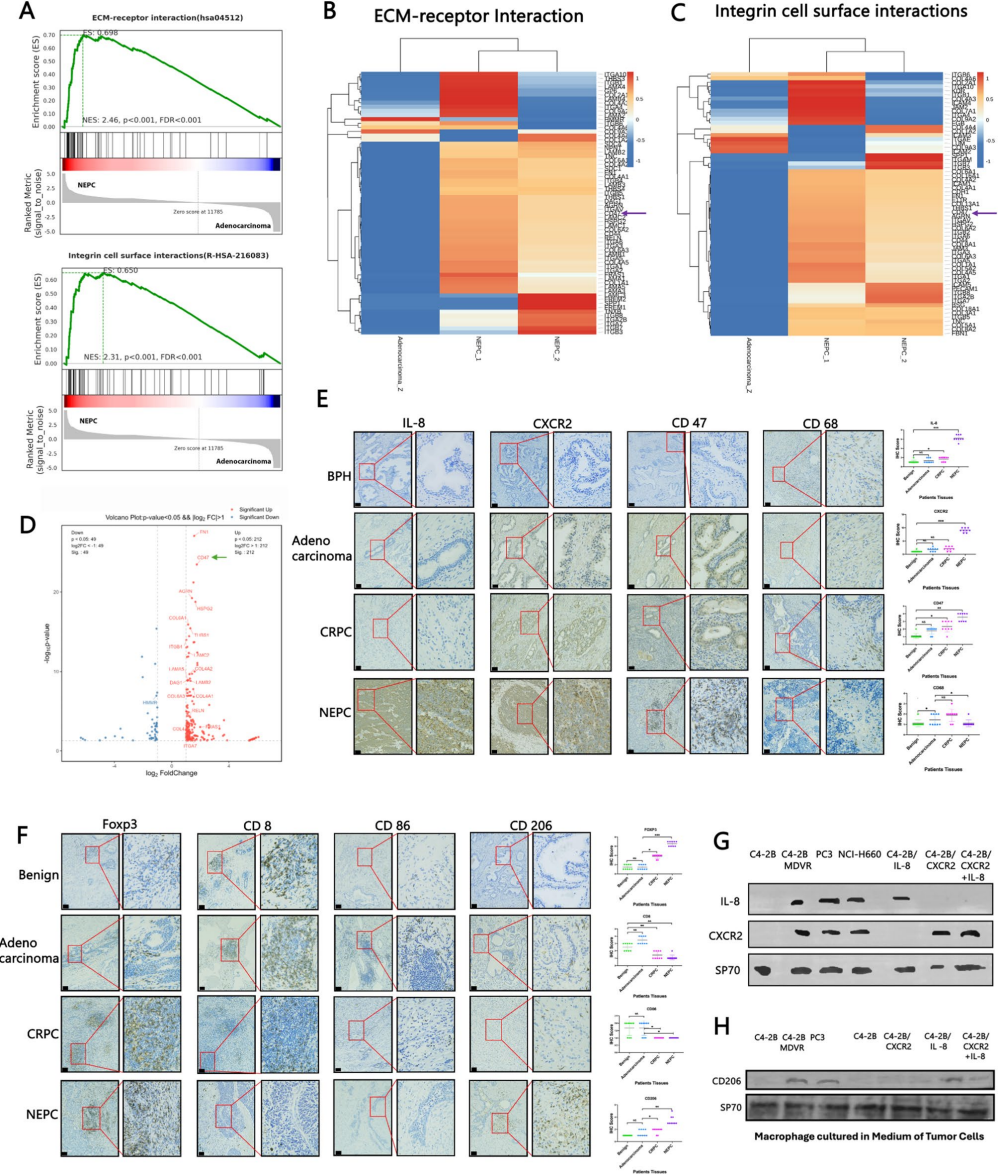
(**A**) GSEA of the ECM‒receptor interaction group and integrin cell surface interaction group compared among the NEPC and adenocarcinoma groups. Heatmap of differentially expressed genes from the comparisons of NEPC and Adenocarcinoma in the ECM-receptor interaction group (**B**) and integrin cell surface interaction group (**C**); **D** The top 20 genes with log2 (fold change) values greater than 1 or less than 1 with adjusted P values lower than 0.05 were considered significantly differentially expressed. **E** Immunohistochemical (IHC) staining for CXCR2, IL‒8, and CD47 expression and the infiltration of TAMs in benign, adenocarcinoma, castration‒resistant prostate cancer (CRPC) and neuroendocrine prostate cancer (NEPC) patient tissue samples; the black bars in the IHC images represent 200 μm. **F** IHC staining for infiltration of TILs and TAMs in benign, adenocarcinoma, CRPC and NEPC patient tissue samples. The black bars in the IHC images represent 200 μm. **G **The expression of IL-8/CXCR2 in several cell lines. **H** The expression of CD206 in macrophages cultured with several cell lines. The data are presented as the means ±SDs of at least three replicates. NS indicates no significance, * indicates *p* < 0.05, ** indicates *p* < 0.01, and *** indicates *p* < 0.001; *p* < 0.05 was considered statistically significant

To explore the role of the IL-8/CXCR2 pathway in shaping the immunosuppressive TME, particularly through its effect on macrophage polarization, we performed transcriptomic sequencing on clinical tissue samples. We compared gene enrichment profiles between the adenocarcinoma and NEPC groups. The NEPC group was significantly enriched in pathways related to extracellular matrix (ECM) interactions and integrin-mediated cell surface interactions (Fig. 1A). Among the genes involved, CD47 was consistently upregulated, appearing prominently in both the ECM interaction (Fig. 1B) and integrin interaction pathways (Fig. 1C). A genome-wide analysis further confirmed that CD47 expression was significantly higher in NEPC tissues than in adenocarcinoma tissues (Fig. 1D). To validate these findings, we performed immunohistochemical (IHC) staining on prostate tissue samples from various stages of prostate cancer and on benign prostate tissues. We used antibodies against IL-8, CXCR2, CD47, CD8, and macrophage markers. NEPC cells presented high expression of IL-8, CXCR2, and CD47, along with reduced infiltration of TAMs (Fig. 1E). In the NEPC tumour microenvironment, TAMs were predominantly polarized towards the M2 phenotype, and there was a noticeable increase in Treg infiltration (Fig. 1F). In contrast, adenocarcinoma samples presented greater infiltration of CD8 + T cells and a greater number of TAMs (Fig. 1E–F). To further confirm these observations, we performed cell-based experiments using several prostate cancer cell lines. C4-2B and C4-2B/MDVR cells, which are commonly used models of castration-resistant prostate cancer (CRPC), were used; notably, C4-2B/MDVR cells present some NEPC phenotypes. To investigate the role of the IL-8/CXCR2 pathway, we overexpressed CXCR2 and IL-8 in C4-2B cells. CXCR2 and IL-8 were detected only in the CRPC and NEPC cell lines (Fig. 1G). Moreover, when lymphocytes were cultured in conditioned media from cells with activated IL-8/CXCR2 signalling, the expression of CD206, a marker of M2-polarized TAMs, was significantly increased (Fig. 1H).

In our previous studies, we reported that neuroendocrine differentiation (NED) in prostate cancer is associated with increased immune evasion by tumour cells [5]– [6]. Consistent with these findings, CD47 expression was significantly elevated in the NEPC group (Fig. 1E), which can be interpreted as a complementary immune evasion mechanism in prostate cancer. Similarly, activation of the CXCR2 pathway led to the clear coexpression of CXCR2 and CD47 in the cell creep immunofluorescence (multiplex staining) experiment (Supplemental Fig. 1 A). Additionally, we identified macrophages (CD11b + F4/80+) by flow cytometry and conducted coculture experiments with immune cells and tumour cells. This experiment was designed to evaluate both macrophage infiltration—differentiated from PBMCs with M-CSF and GM-CSF—and phagocytic capacity. When cultured with IL-8/CXCR2-expressing cell lines (C4-2B/MDVR, PC3, and NCI-H660), macrophage infiltration was significantly reduced, and the ability of these cells to engulf tumour cells was also impaired (Supplemental Fig. 1B). Moreover, we assessed the differentiation of PBMCs (stimulated with IL-2, M-CSF, and GM-CSF) into CD8 + T cells, CD4 + Tregs, M1 macrophages (CD86⁺), and M2 macrophages (CD206⁺). Culture with tumour cell supernatants led to reduced Ki67 expression in CD8 + T cells and a decrease in M1 macrophage infiltration, whereas CD4 + Tregs presented increased Ki67 expression and a corresponding increase in M2 macrophage infiltration (Supplemental Fig. 1 C). Activation of the IL-8/CXCR2 pathway resulted in decreased production of cytotoxic cytokines by M1 macrophages but promoted increased secretion of immunosuppressive cytokines by M2 macrophages (Supplemental Fig. 1D). Collectively, these findings indicate that the IL-8/CXCR2 signalling axis plays a key role in modulating TAM infiltration and polarization within the prostate cancer microenvironment.

### The IL-8/CXCR2 pathway in tumour cells attenuates the migration of macrophages

**Fig. 2:**
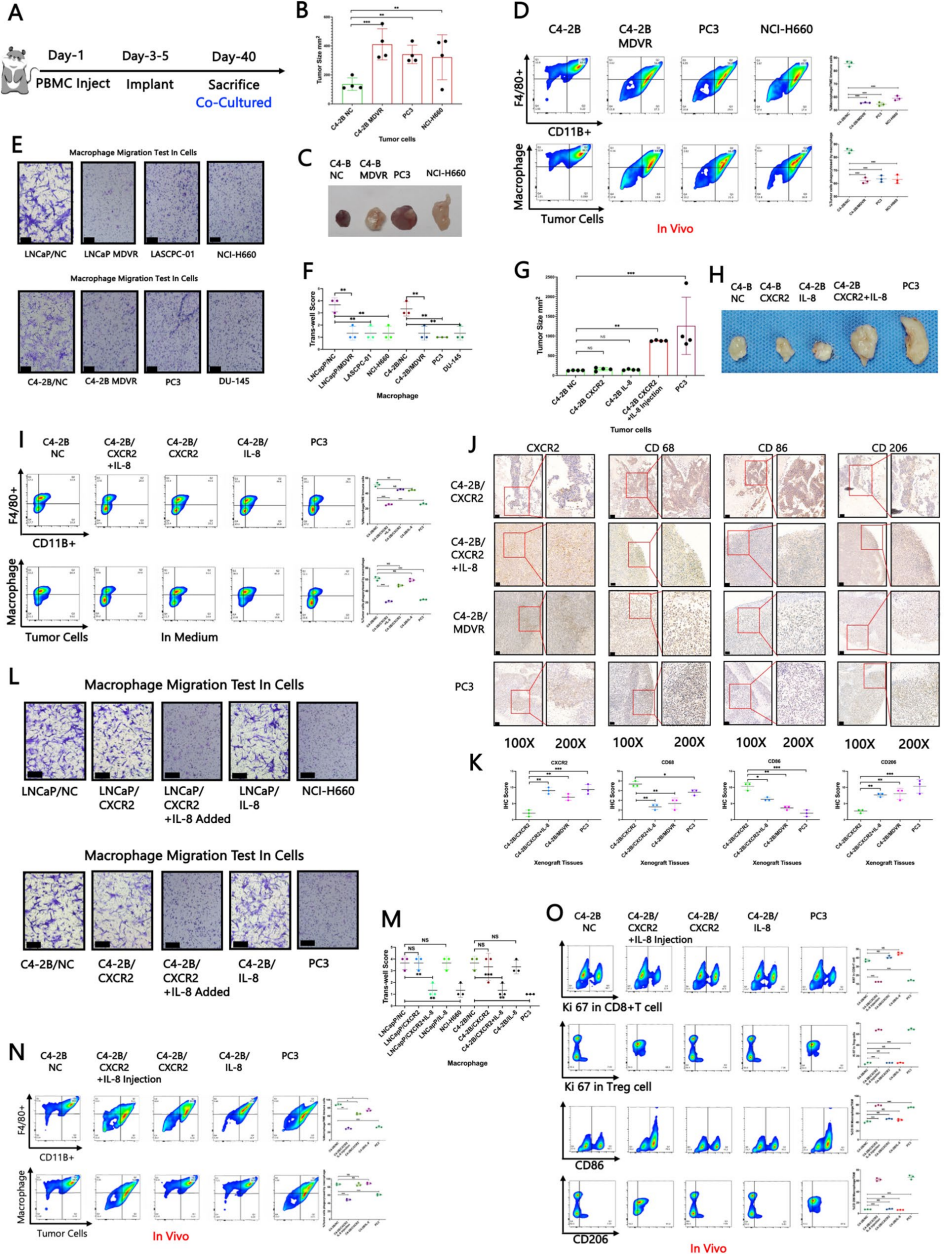
(**A**-**C**) Mouse experiment diagram drawing and tumour size in several tumour-bearing mouse models. **D **Tumour-associated macrophages (CD11b + F4/80+) identified by flow cytometry from tumours collected from mice bearing tumours generated from different cell lines and the ability of tumour-associated macrophages to engulf tumour cells identified by flow cytometry; the data points represent biological replicates derived from three individual mice.** E-F **Macrophage migration test by the Transwell method in several cell Lines; the black bars in the images represent 200 μm. **G-H **Tumour size in different tumour-bearing mice (constructed from different cell lines); each experimental group included three biological replicates. **I **Macrophages (CD11b + F4/80+) identified by flow cytometry from a coculture model of immune cells and tumour cells; the ability of macrophages to engulf tumour cells was assessed by flow cytometry, and the data points represent biological replicates derived from three individual mice (each experiment was independently repeated three times to ensure reproducibility).** J-K** IHC staining of CD47, CD68, CD86, and CD206 in tissue samples collected from several tumour-bearing mouse models; the black bars in the IHC images represent 200 μm, and data points represent biological replicates derived from three individual mice (each experiment was independently repeated three times to ensure reproducibility). **L-M** The migration ability of macrophages after activation of the IL-8/CXCR2 pathway; the black bars in the images represent 200 μm, with each experiment independently repeated three times to ensure reproducibility. **N **Tumour-associated macrophages (CD11b + F4/80+) were assessed by flow cytometry from tumours collected from mice bearing tumours constructed by different cell lines in which IL-8/CXCR2 pathway was highly active; the ability of tumour-associated macrophages to engulf tumour cells when the IL-8/CXCR2 pathway was active was assessed by flow cytometry, and data points represent biological replicates derived from three individual mice (each experiment was independently repeated three times to ensure reproducibility). **O **Cell proliferation of tumour-infiltrating CD8 + T cells and CD4 + Tregs determined by Ki67 expression and the expression of Ki67 in tumour-infiltrating CD8 + T cells and CD4 + Tregs; the infiltration of M1 macrophages (CD86⁺) and M2 macrophages (CD206⁺) was assessed by flow cytometry when the IL-8/CXCR2 pathway was active, and data points represent biological replicates derived from three individual mice (each experiment was independently repeated three times to ensure reproducibility). The data are presented as the means ±SDs. NS indicates no significance, * indicates *p* < 0.05, ** indicates *p* < 0.01, and *** indicates *p* < 0.001; *p* < 0.05 was considered statistically significant

To further validate these findings, we conducted in vivo experiments (Fig. 2A). Compared with control tumours, tumours with activated IL-8/CXCR2 signalling were significantly larger (Fig. 2B–C). Additionally, flow cytometry analysis revealed reduced infiltration and phagocytic activity of macrophages within these tumours (Fig. 2D). To better characterize the immunosuppressive effects of the IL-8/CXCR2 axis, we tested macrophage migration towards various cell lines with a Transwell assay. In NEPC cell models expressing IL-8/CXCR2, macrophage migration and tumour cell phagocytosis were markedly decreased (Fig. 2E–F). To further explore the mechanistic role of CXCR2 in immune evasion, we administered exogenous human IL-8 to NOD-SCID gamma (NSG) mice and monitored tumour growth. The results demonstrated that IL-8, in combination with its receptor CXCR2, impaired tumour responsiveness to PBMC treatment (Fig. 2G–H). Notably, exogenous human IL-8 did not activate the CXCR1 pathway and could not facilitate tumour escape from PBMC therapy (Supplemental Fig. 2 A). In vitro coculture models supplemented with human IL-8 presented consistent trends, including reduced macrophage infiltration and phagocytosis of tumour cells (Fig. 2I). Furthermore, IL-8 stimulation promoted the differentiation of PBMCs into CD4 + Tregs and M2 macrophages while decreasing the proportions of CD8 + T cells and M1 macrophages, as confirmed in vivo in multiple tumour models (Supplemental Fig. 2B). CXCR2 activation also led to increased Ki67 expression in Tregs and elevated infiltration of M2 macrophages, in addition to blocking the migration of CD8 + T cells and M1 macrophages, as verified in IL-8-supplemented coculture models (Supplemental Fig. 2 C). Consistent with these results, IHC analysis of tumour-bearing mouse tissues revealed that elevated CXCR2 expression was correlated with decreased levels of CD68 and CD86 (markers of macrophages and M1 polarization) and increased expression of the M2 marker CD206 (Fig. 2J–K). To further verify the role of the IL-8/CXCR2 pathway in regulating TAM migration, we conducted a Transwell migration assay to investigate how CXCR2 activation modulates TAM behaviour. Upon the addition of IL-8 to activate the CXCR2 pathway, a significant reduction in macrophage migration was observed, as reflected by decreased clonogenicity in the lower chamber (Fig. 2L–M). Additionally, flow cytometry analysis of tumours harvested from mice implanted with different cell lines and treated with IL-8 revealed a decreased population of TAMs; CD11b⁺F4/80⁺) (Fig. 2N). The ability of TAMs to engulf tumour cells also decreased after activation of the IL-8/CXCR2 pathway (Fig. 2N). Following activation of the IL-8/CXCR2 pathway, Ki67 expression in CD8 + T cells and infiltration of M1 macrophages in the TME decreased, whereas Ki67 expression in CD4 + Tregs and infiltration of M2 macrophages increased (Fig. 2O). Additionally, ELISA analysis revealed that the levels of cytokines secreted by tumour-associated M2 macrophages increased and that the levels of M1-associated cytokines concomitantly decreased in response to IL-8/CXCR2 pathway activation (Supplemental Fig. 2D).

Given the observed coexpression of CXCR2 and CD47 (Fig. 1E), we propose that the IL-8/CXCR2 pathway may regulate CD47 expression, thereby impairing TAM recruitment and increasing tumour immune evasion. Therefore, further investigations are warranted to elucidate the precise molecular mechanisms by which IL-8/CXCR2 regulate CD47 expression and M2 macrophage polarization.

### The IL-8/CXCR2 pathway redefines the metabolic profile of tumour cells

**Fig. 3:**
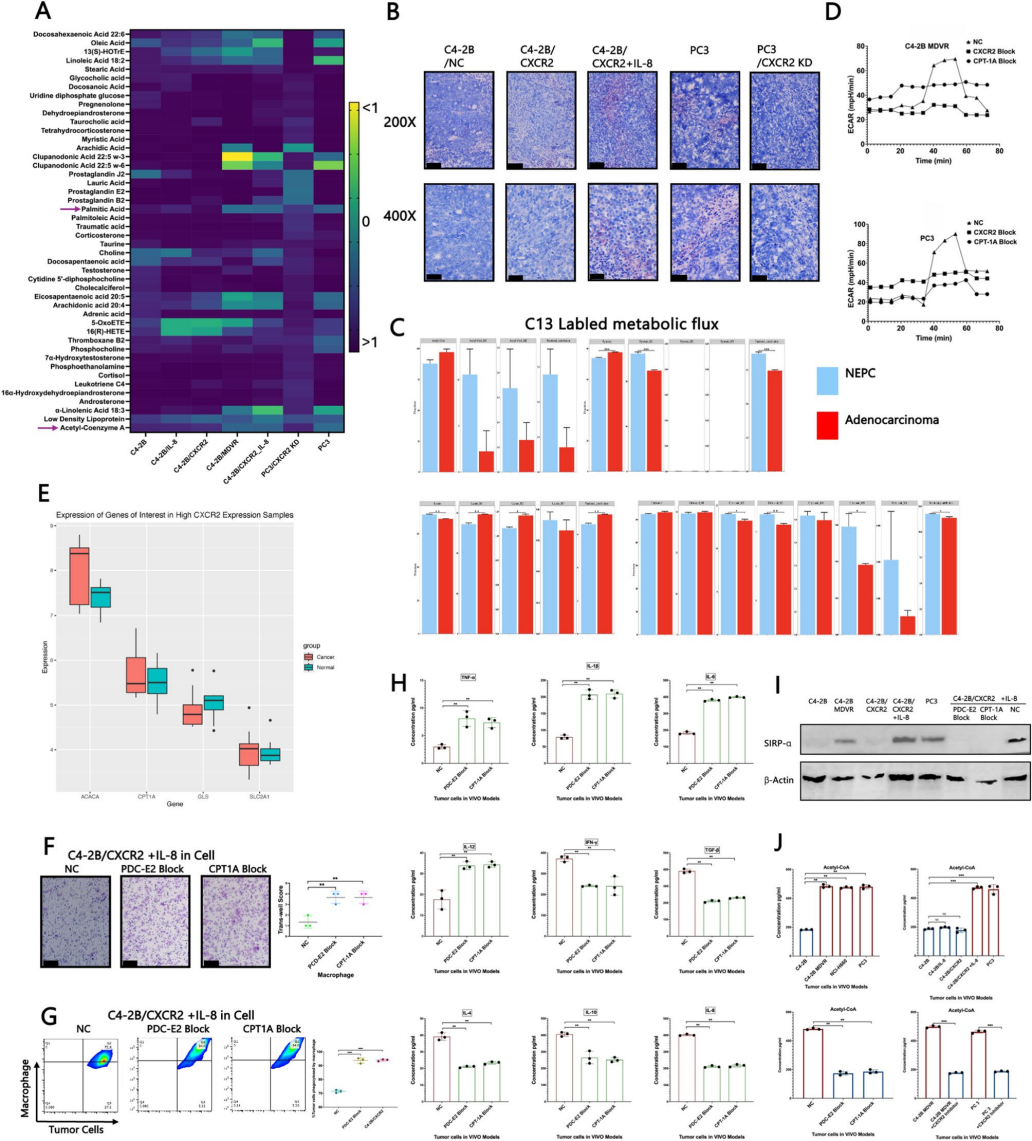
(**A**) Metabolomic mass spectrometry of samples collected from mice bearing tumours constructed from different cell lines.** B** Representative images of cytoplasmic Lipid accumulation detected by Oil Red O staining of samples from mice bearing tumours constructed from different cell Lines; the black bars in the images represent 200 μm. **C **^13^C-labelled metabolic flux to trace the origin of acetyl-CoA in the process of glucose metabolism. **D **Seahorse X assay results for the ECAR of C4-2B MDVR and PC3 cells after CXCR2 blockade and CPT-1 A blockade. **E **Expression of ACACA, CPT1A, GLS and SLC2A1 in the CXCR2-expressing group versus the normal group. **F **Transwell results of macrophages after culture in different media with supernatants (C4-2B/CXCR2 cells cultured in medium with IL-8 and treated with PDC-E2/CPT-1 A inhibitors for 24 h, followed by collection of the supernatants) or 1640 medium; the black bars in the images represent 200 μm, and each experimental group included three biological replicates. **G **The ability of macrophages to engulf tumour cells from the coculture model of immune cells and tumour cells after PDC-E2/CPT-1 A was blocked (each experimental group included three biological replicates). **H** Cell factors secreted by tumour-associated macrophages after PDC-E2/CPT-1 A was blocked were assessed by ELISA, with standard concentrations prepared by serial 1:2 dilutions of a recombinant protein standard provided by the ELISA Kit; data points represent biological replicates derived from three individual mice and each experiment was independently repeated three times to ensure reproducibility. **I **Western blot of SIRP-α in several cell lines; **J **ELISA of acetyl-CoA in several tumour model; data points represent biological replicates derived from three individual mice, and each experiment was independently repeated three times to ensure reproducibility. The data are presented as the means ±SDs. NS indicates no significance, * indicates *p* < 0.05, ** indicates *p* < 0.01, and *** indicates *p* < 0.001; *p* < 0.05 was considered statistically significant

Metabolic mass spectrometry (MS) analysis revealed significant upregulation of lipid metabolism in tumour-bearing mouse models generated from various tumour cell lines with an activated IL-8/CXCR2 pathway (Fig. 3A). Notably, acetyl-CoA (AC-CoA) levels were markedly elevated in association with IL-8/CXCR2 activation (Fig. 3A). Consistent with these findings, Oil Red O staining demonstrated increased cytoplasmic lipid accumulation in tumours derived from cells with an activated IL-8/CXCR2 pathway (Fig. 3B). These findings align with previous reports indicating that high-grade prostate cancer cells preferentially engage in lipid metabolism [12] and that lipid metabolism often coexists with immune evasion mechanisms in advanced cancers [13]. To characterize the lipid metabolic landscape further, we performed differential gene expression analysis between NEPC and adenocarcinoma patient tissues, including Gene Ontology (GO) enrichment (Supplemental Fig. 3 A), Kyoto Encyclopedia of Genes and Genomes (KEGG) pathway analysis (Supplemental Fig. 3B), Clusters of Orthologous Groups (COG)/Karyotic Orthologous Groups (KOG) classification (Supplemental Fig. 3 C), and LipidMaps annotation (Supplemental Fig. 3D). All analyses consistently revealed elevated lipid metabolic activity in the NEPC group. Subcellular localization analysis revealed that the majority of affected biological processes were cytoplasmic (44.45%), suggesting increased lipid synthesis and cell proliferation in NEPC (Supplemental Fig. 3E). Additionally, a substantial proportion of mitochondrial involvement (15.14%) was associated with increased mitochondrial metabolic activity (Supplemental Fig. 3E). Human Metabolome Database (HMDB) annotation further underscored the enrichment of lipid-associated molecular processes in NEPC tissues (Supplemental Fig. 3 F). In addition to lipid metabolism, prostate cancer cells also rely on glycolysis for energy production [14]. ^13 C-labelled metabolic flux analysis demonstrated that AC-CoA was partly derived from glucose metabolism (Fig. 3C). Moreover, Seahorse XF assays revealed that inhibition of CPT1A impaired mitochondrial function, and a similar reduction in mitochondrial activity was observed upon CXCR2 pathway blockade (Fig. 3D).

Western blot analysis across multiple cell lines revealed elevated expression of both PDC-E2 and CPT1A in those with an activated IL-8/CXCR2 pathway. PDC-E2 catalyses the conversion of pyruvate—a key glycolysis byproduct—into acetyl-CoA, offering complementary evidence that links glycolytic flux to acetyl-CoA production and emphasizing the pivotal role of PDC-E2. (Supplemental Fig. 4A–B). PCR analysis supported these findings (Supplemental Fig. 4 C). Pharmacological inhibition of acetyl-CoA metabolism via PDC-E2 blockade, as well as suppression of palmitate synthesis through FASN inhibition, resulted in a significant reduction in CD47 expression in tumour cells. A significant reduction in membrane-localized CD47 was also observed upon CXCR2 inhibition (Supplemental Fig. 4D). Importantly, CXCR1 expression remained unchanged under these conditions (Supplemental Fig. 4D). Together with the transcriptomic data presented in Fig. 2A, these findings strongly indicate that CXCR1 does not exhibit compensatory upregulation or function as a substitute for CXCR2 activity. These findings support the conclusion that CXCR2 serves as the principal mediator of IL-8–driven metabolic and immunological regulation in this setting. The elevated expression levels of both PDC-E2 and CPT1A suggest that CD47 upregulation is associated with metabolic reprogramming involving increased glycolysis, altered lipid metabolism, and increased acetyl-CoA levels. Consistent with these findings, cell creep immunofluorescence staining for CPT1A, PDC-E2, and CD47 revealed similar expression patterns (Supplemental Fig. 4E). Furthermore, immunofluorescence analysis of tumour tissues from tumour-bearing mice confirmed the coexpression of CXCR2, CPT1A, and CD47, supporting a functional connection between the IL-8/CXCR2 pathway and metabolic regulation (Supplemental Fig. 4 F).

We cocultured PC3 prostate cancer cells with PBMCs isolated from the spleens of NSG mice to evaluate changes in CXCR1 and CD47 expression in tumour cells and alterations in immune cell infiltration within the tumour microenvironment under conditions involving the inhibition of two key metabolic enzymes (PDC-E2 and FAS) or blockade of the CXCR2 signalling pathway (Supplemental Fig. 5 A). CXCR1 expression remained largely unaffected by these interventions, suggesting that it may be regulated independently of metabolic or chemokine signalling. In contrast, CD47 expression was significantly reduced following treatment and was closely associated with decreased infiltration of M2-like macrophages. Furthermore, Treg proliferation was moderately suppressed, whereas CD8⁺ T-cell proliferation and NK cell Ki67 expression were markedly increased, indicating the activation of multiple effector immune populations (Supplemental Fig. 5 A). Notably, CXCR2 blockade significantly increased macrophage migratory capacity in this coculture system (Supplemental Fig. 5B), suggesting that the CXCR2 axis may act as a negative regulator of macrophage chemotaxis.

To further elucidate the influence of lipid metabolism and glycolysis on CXCR2 expression, we conducted bioinformatics analysis using publicly available datasets. The results revealed that lipid metabolism-related enzymes—ACACA, CPT1A, and the glucose transporter SLC2A1—were significantly upregulated in the CXCR2-high group (Fig. 3E). Transwell migration assays confirmed increased macrophage migration in response to conditioned medium from IL-8-treated C4-2B/CXCR2 cells, along with treatment with inhibitors targeting PDC-E2 or CPT1A for 24 h (Fig. 3F). Additionally, macrophage-mediated phagocytosis of tumour cells increased when tumour cells were pretreated with PDC-E2 or CPT1A inhibitors (Fig. 3G). Interestingly, inhibition of PDC-E2 or CPT1A resulted in reduced secretion of M1 macrophage-associated cytokines and a concurrent increase in M2-associated cytokines (Fig. 3H), suggesting a shift towards a protumoral macrophage phenotype. Western blot analysis further demonstrated robust expression of signal-regulatory protein alpha (SIRPα) in the presence of an active IL-8/CXCR2 pathway, which was abolished when PDC-E2 or CPT1A was inhibited (Fig. 3I). ELISAs confirmed increased intracellular acetyl-CoA levels in IL-8/CXCR2-high cell lines, a trend that was significantly reversed upon CXCR2 blockade (Fig. 3J). Given that acetyl-CoA is a key metabolic product of both glycolysis and lipid oxidation [15], these findings suggest that IL-8/CXCR2 signalling promotes metabolic reprogramming to fuel tumour progression. Overall, we propose that the IL-8/CXCR2–acetyl-CoA axis upregulates CD47 expression and, through the CD47–SIRPα interaction, suppresses the antitumour functions of TAMs, thereby promoting immune evasion.

### Activation of the tumour-intrinsic IL-8/CXCR2 pathway promotes CD47 upregulation through increased acetylation of the transcription factor p65

To further elucidate the mechanism by which the IL-8/CXCR2 pathway regulates CD47 expression, we conducted in-depth analyses. GO biological process (BP) analysis revealed increased NF-κB signalling activity in NEPC compared with adenocarcinoma, whereas molecular function (MF) analysis revealed increased AC-CoA metabolism. KEGG pathway analysis also suggested increased lipid metabolic activity in NEPC (Supplemental Fig. 5 C). Consistently, tumour tissues from C4-2B/MDVR model mice presented increased lipid metabolism and AC-CoA activity relative to tumour tissues from C4-2B/NC model mice (Supplemental Fig. 5D), both of which were suppressed upon CXCR2 inhibition (Supplemental Fig. 5E). These results prompted us to focus specifically on the role of acetyl-CoA in mediating the downstream effects of IL-8/CXCR2 signalling.

Previous studies have demonstrated that NF-κB signalling is modulated by acetylation of the p65 (RelA) subunit [16] and that such acetylation can increase CD47 expression [17]. Consistent with these findings, our Western blot analysis of multiple cell lines confirmed that IL-8/CXCR2 activation increased the levels of RelA acetylated at lysine 310 (K310), global histone 3 acetylation, and CD47 expression (Supplemental Fig. 5 F). Notably, the nuclear localization of K310-acetylated p65 was increased following IL-8/CXCR2 activation, whereas total nuclear p65 levels and DNA-binding capacity remained unchanged (Supplemental Fig. 5G). These findings suggest that acetylation, rather than redistribution, modulates NF-κB transcriptional activity. Additionally, treatment with palmitate similarly increased p65 acetylation (Supplemental Fig. 5H), which links lipid metabolism to NF-κB activation. Furthermore, we generated a lysine-to-arginine point mutation (K310R) in the RelA/p65 expression plasmid by site-directed mutagenesis to prevent acetylation at the K310 site in C4-2B cells. This mutation significantly reduced RelA/p65 binding to the CD47 promoter, as demonstrated by chromatin immunoprecipitation assays (Supplemental Figs. 5I–J). To validate these observations, gene set enrichment analysis (GSEA) of public transcriptomic datasets was used to identify differential gene expression patterns enriched in the NF-κB pathway (Supplemental Fig. 5 K). We then used luciferase reporter assays to assess NF-κB transcriptional activity. Treatment with fatty acid oxidation (FAO) activators—palmitate, L-carnitine, and citrate—as well as AC-CoA significantly increased luciferase activity (Supplemental Fig. 6A–B). Concordantly, nuclear staining of K310-acetylated p65 increased under these treatments, whereas total p65 localization and DNA-binding activity remained constant (Supplemental Fig. 6 C). Together, these findings suggest that IL-8/CXCR2-induced metabolic reprogramming promotes CD47 expression by increasing AC-CoA levels, which increases p65 acetylation and subsequent NF-κB pathway activation, thereby supporting tumour immune evasion.

### S-palmitoylation mediated by ZDHHC2 promotes CD47 PM localization in prostate cancer

**Fig. 4:**
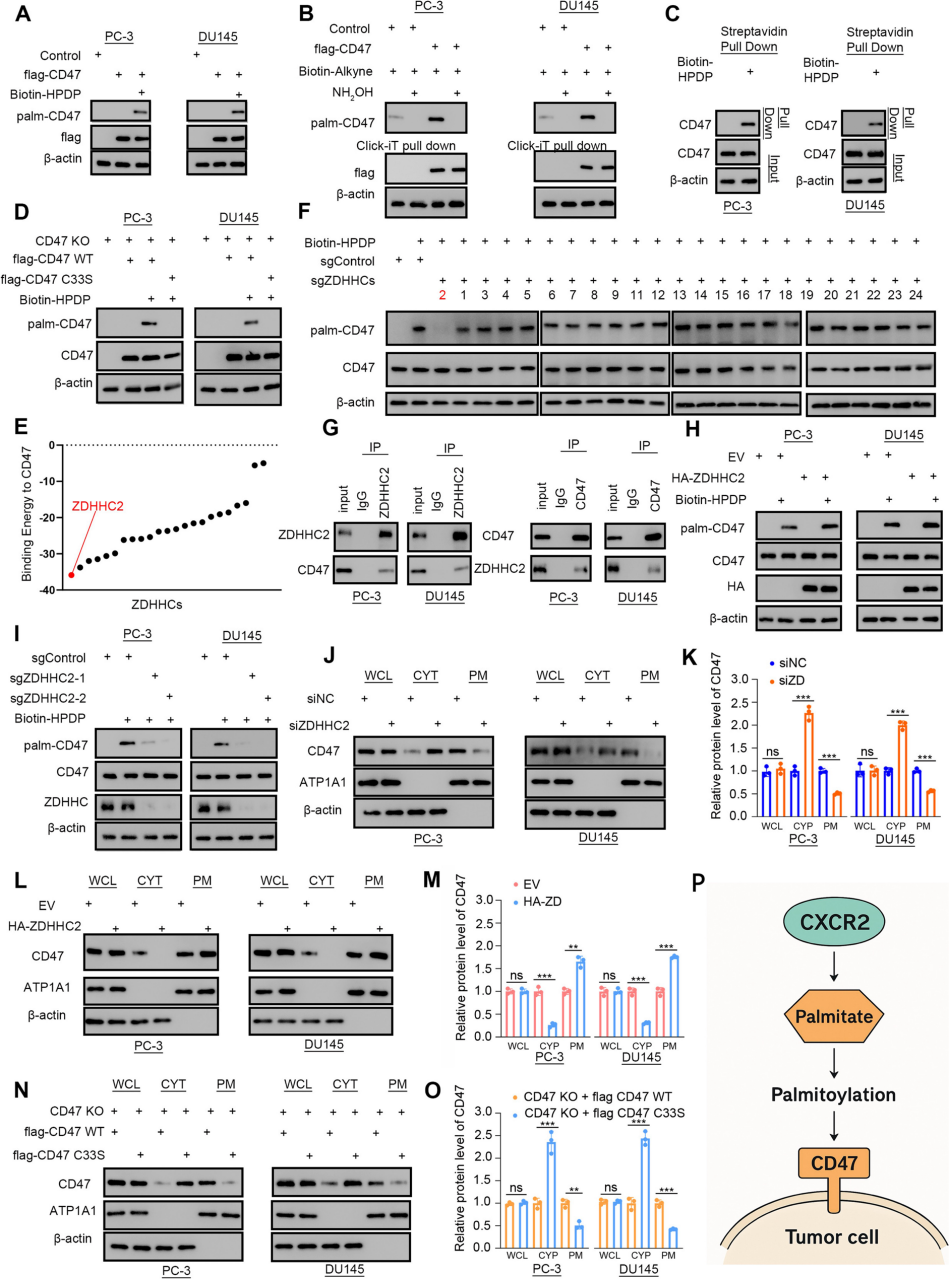
S-palmitoylation mediated by ZDHHC2 promotes CD47 PM localization in prostate cancer. PC-3 and DU145 cell lines were transfected with empty vector (EV) or flag-CD47 plasmids for 24 h, and then the ABE assay (A) and Click-iT pull-down assay (B) were performed for CD47 palmitoylation detection. **C **An ABE assay was used for endogenous CD47 palmitoylation detection in PC-3 and DU145 cell lines; in these cell-based experiments, each experimental group included three biological replicates. (D-E) Endogenous CD47 was knocked out at the C33S site (D), and then, CD47 was labelled with biotin-HPDP for streptavidin pull-down to analyse the palmitoylation level (E). **F **Calculation of the binding energy of palmitoyl acyltransferases to CD47. (G) Palmitoylation of CD47 was assessed by the ABE assay after PC-3 cells were infected with lentiviruses containing expression cassettes of control guide RNA or guide RNAs targeting different ZDHHCs and the Cas9 protein. **H **Immunoprecipitation analysis was performed in the lysates of PC-3 and DU145 cells with ZDHHC2 and CD47 antibodies. CD47 was labelled with biotin-HPDP for streptavidin pull-down to analyse the palmitoylation level after PC-3 and DU145 cell lines were transfected with empty vector (EV) or HA-ZDHHC2 plasmids for 24 h (I) or PC-3 and DU145 cell lines that were infected with lentiviruses containing expression cassettes of control guide RNA or guide RNAs targeting ZDHHC2 and the Cas9 protein (J-K). **L-M** After PC-3 and DU145 cells were transfected with empty vector (EV) or HA-ZDHHC2 plasmids for 48 h, the subcellular fractions were extracted and subjected to Western blot analysis. **N-O** PC-3 and DU145 cell lines with CD47 knockout were transfected with empty vector (EV), flag-CD47 wild-type or flag-CD47 C33S mutant plasmids for 48 h. The subcellular fractions were extracted and subjected to Western blot analysis after endogenous CD47 was knocked out.** P** Summary of the findings described above. The data are presented as the means ±SDs of at least three replicates. NS indicates no significance, * indicates *p* < 0.05, ** indicates *p* < 0.01, and *** indicates *p* < 0.001; *p* < 0.05 was considered statistically significant

CD47 is known to engage with SIRPα on macrophages, thereby suppressing the phagocytic activity of TAMs at the plasma membrane (PM) [18]. We also observed that palmitate production was upregulated via the IL-8/CXCR2 pathway (Fig. 3A). To dissect the distinct roles of palmitate and AC-CoA in modulating TAM activity, we first performed Transwell migration assays. Both palmitate and AC-CoA significantly suppressed macrophage migration, with palmitate-treated tumour cells presenting a more pronounced inhibitory effect (Supplemental Fig. 6D). We then assessed the subcellular distribution of CD47 under these conditions. While both metabolites increased total CD47 levels in whole-cell lysates (WCLs), palmitate more robustly promoted CD47 accumulation at the plasma membrane (Supplemental Fig. 6E–F). Given these findings, we focused on S-palmitoylation, a posttranslational modification known to modulate protein trafficking and membrane localization [19]. Using the prostate cancer cell lines PC-3 and DU145 transfected with FLAG-tagged CD47, we used the acyl-biotinyl exchange (ABE) method with biotin-HPDP followed by streptavidin blotting to confirm CD47 palmitoylation (Fig. 4A). Additional validation with biotin-alkyne labelling and the Click-iT reaction revealed increased CD47 palmitoylation upon overexpression and decreased levels following hydroxylamine (NH₂OH) treatment (Fig. 4B). Endogenous palmitoylation of CD47 was further confirmed by streptavidin pull-down (Fig. 4C). To identify the specific palmitoylation site, we used the CSS-Palm4.0 prediction tool, which suggested cysteine 33 (C33) as a candidate. A mutant CD47 construct with C33 substituted with serine (C33S) exhibited a complete loss of palmitoylation, confirming that C33 was the modification site (Fig. 4D). Molecular docking simulations identified ZDHHC2 as the palmitoyl acyltransferase with the highest predicted binding affinity for CD47 (Fig. 4E). We then generated a panel of PC-3 cell lines with CRISPR-mediated knockouts of various ZDHHC genes. Only ZDHHC2 knockout resulted in the loss of CD47 palmitoylation (Fig. 4F). Coimmunoprecipitation assays in PC-3 and DU145 cells confirmed the direct interaction between CD47 and ZDHHC2 (Fig. 4G). Further experiments revealed that ZDHHC2 overexpression increased CD47 palmitoylation, whereas ZDHHC2 knockout reduced it (Fig. 4H–I). Subcellular fractionation demonstrated that ZDHHC2 knockdown significantly decreased CD47 localization to the plasma membrane, whereas ZDHHC2 overexpression had the opposite effect. Importantly, total CD47 levels in WCLs remained unchanged (Fig. 4J–M). Finally, a comparison between wild-type CD47 and the C33S mutant revealed that the mutant had markedly reduced plasma membrane localization (Fig. 4N–O). In conclusion, our findings demonstrate that ZDHHC2-mediated S-palmitoylation of CD47 at C33 is essential for its localization to the plasma membrane, a process critical for its ability to suppress macrophage-mediated antitumour responses (Fig. 4P).

### Elevated CD36 expression in TAMs plays a key role in promoting M2 macrophage polarization

**Fig. 5:**
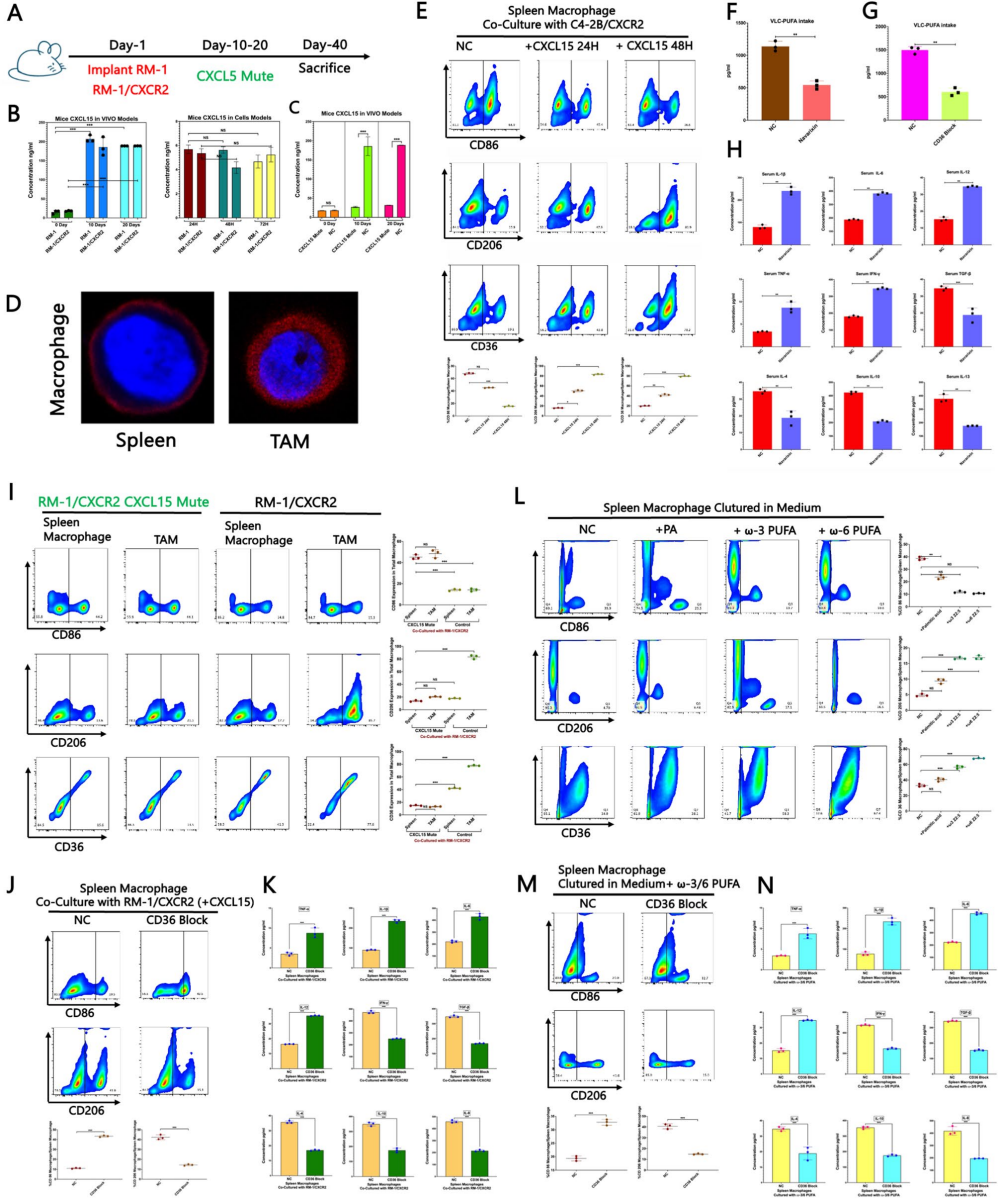
(**A**) Mouse experiment diagram drawing. **B-C** In vivo analysis of CXCL15 secretion in CXCL15Mutant/NC C57BL/6 tumour-bearing models established with RM-1 and RM-1/CXCR2 cells, along with CXCL15 production at the cellular level. **D **Immunofluorescence staining of macrophages and CD8 + T cells within the murine TME engulfing a substantial amount of lipids. **E **Spleen macrophage differentiation and CD36 expression after coculture with C4-2B/CXCR2 tumour cells in medium with CXCL15 for 24 and 48 h. **F** Metabolomic analysis of lipid uptake by macrophages after treatment with navarixin in the NSG model. **G **Metabolomic analysis of lipid uptake by macrophages after CD36 blockade at the cellular level. **H **Cell factors secreted by macrophages were assessed by ELISA of the serum samples of NSG mice after treatment with navarixin.** I** TAM differentiation in CXCL15-mutant C57BL/6 model mice. **J-K** Polarization of spleen macrophages cocultured with tumour cells under CD36 blockade conditions. **L** Polarization of spleen macrophages cultured with palmitic acid or ω−3/6 PUFAs. **M-N** Polarization of spleen macrophages cultured with ω−3/6 PUFAs under CD36 blockade conditions; data points represent biological replicates derived from three individual mice, and each experiment was independently repeated three times to ensure reproducibility. The data are presented as the means ±SDs of at least three replicates. NS indicates no significance, * indicates *p* < 0.05, ** indicates *p* < 0.01, and *** indicates *p* < 0.001; *p* < 0.05 was considered statistically significant

Our previous research demonstrated that CD47 expression in tumour cells partially accounts for its effect on macrophage infiltration within the tumour microenvironment (TME) [6]. However, these findings do not fully explain the observed M2 polarization of macrophages. Notably, macrophages within TMEs enriched with CD47^high tumour cells presented elevated expression of M2 markers (Supplemental Fig. 7 A), which prompted further investigation. To explore this, we orthotopically implanted RM-1 prostate cancer cells or RM-1 cells overexpressing CXCR2 into the prostates of C57BL/6 mice (Fig. 5A). Given that CXCL15 is the murine homologue of human IL-8, we generated CXCL15-mutant mice to suppress CXCL15/CXCR2 signalling (Fig. 5A). ELISAs revealed that CXCL15 secretion within the TME increased significantly with tumour growth in both the RM-1 and RM-1/CXCR2 models, peaking at approximately Day 10, whereas the intracellular CXCL15 levels remained unchanged (Fig. 5B–C). Our prior work linked TAM M2 polarization to lipid uptake, although the mechanism remains unclear [6]. To investigate this, we isolated macrophages from both the spleen and the TME and assessed lipid uptake. Metabolomic profiling of RM-1/CXCR2 tumours revealed elevated palmitic acid and ω−3/ω−6 very-long-chain polyunsaturated fatty acids (VLC-PUFAs) in the TME (Supplemental Fig. 7B–C), and TAMs exhibited significantly greater lipid uptake than did spleen-derived macrophages (Fig. 5D). CD36, a scavenger receptor known to mediate lipid uptake and promote M2 polarization [20], was previously shown to play a role in this process [6]. To assess the involvement of CXCL15/CXCR2 signalling, spleen-derived macrophages were cultured with CXCL15 in vitro, which resulted in increased CD36 expression and M2 marker upregulation after 48 h (Fig. 5E). Similarly, in NSG mice, blockade of either CXCR2 or CD36 significantly reduced TAM uptake of VLC-PUFAs (Fig. 5F–G) and suppressed M2-associated cytokine secretion (Fig. 5H). In CXCL15-mutant mice, disruption of the signalling axis impaired M2 polarization and downregulated CD36 in TME-infiltrating macrophages (Fig. 5I). Furthermore, in a coculture system of RM-1/CXCR2 tumour cells with spleen-derived macrophages, CD36 inhibition markedly reduced M2 polarization (Fig. 5J–K). Because the CXCL15/CXCR2 axis increased palmitate and ω−3/6 VLC-PUFA accumulation in the TME, we evaluated their direct effects on macrophage polarization. In vitro, ω−3 and ω−6 VLC-PUFAs strongly promoted M2 polarization, whereas palmitate had a limited effect (Fig. 5L). Notably, this VLC-PUFA-driven polarization was abolished by CD36 inhibition (Fig. 5M–N).

**Fig. 6:**
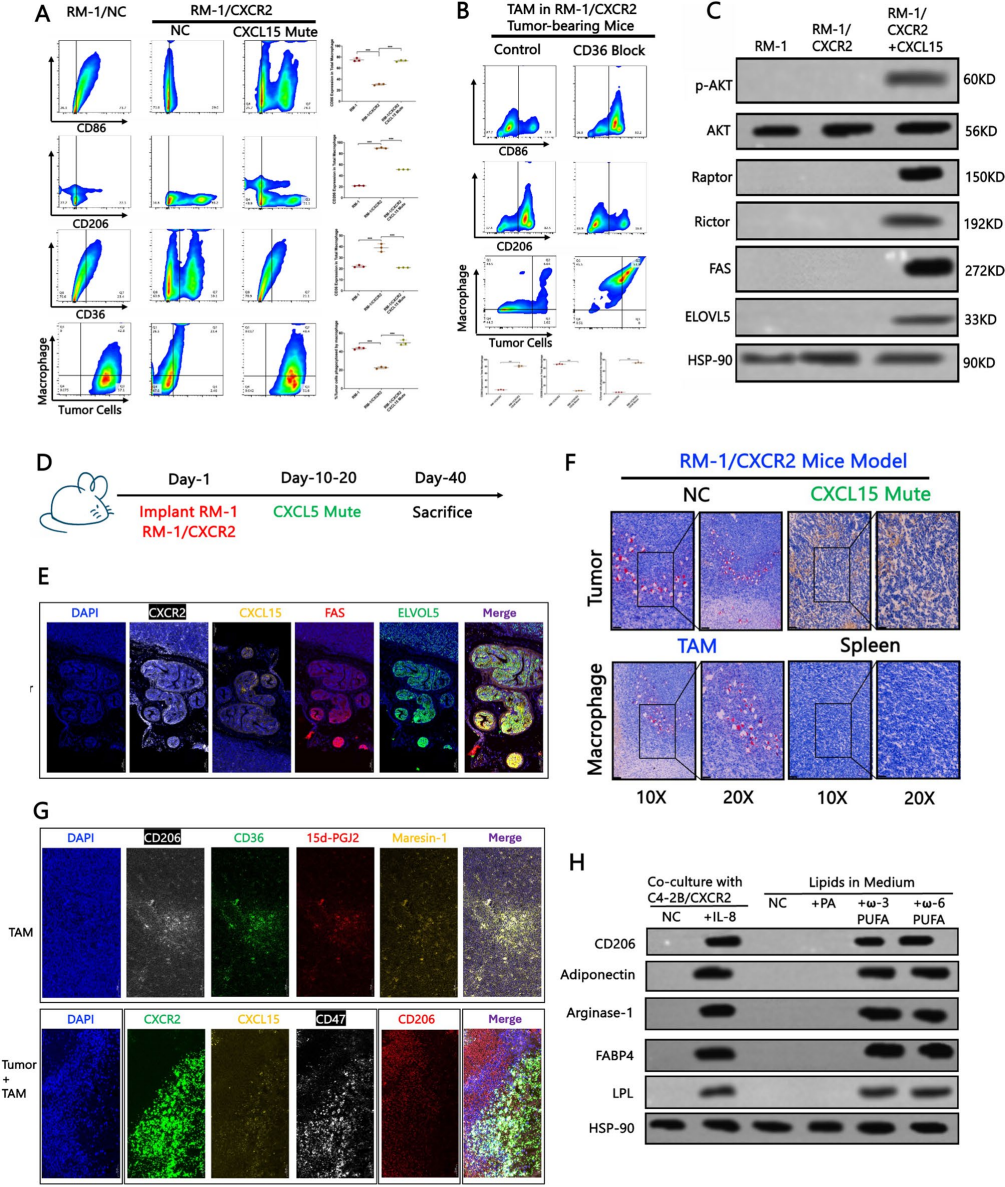
(A–B) Analysis of macrophage differentiation and CD36 expression in TAMs from C57BL/6 mouse models with or without CXCL15 mutation or CD36 inhibition. **C** Western blot analysis of the AKT–mTORC1–FASN and AKT–mTORC2–ELOVL5–ω−3/6-PUFA pathways in RM-1 and RM-1/CXCR2 cell line models to determine the source of ω−3/6 PUFAs in the TME. **D** Mouse experiment diagram drawing. **E **Immunofluorescence staining of CXCL15, CXCR2, FAS, and ELOVL5 in tumour tissues from RM-1/CXCR2 tumour-bearing C57BL/6 mouse models; data points represent biological replicates derived from three individual mice, and each experiment was independently repeated three times to ensure reproducibility.** F** Oil Red O staining was performed on tumour tissues from RM-1/CXCR2 tumour-bearing C57BL/6 mice, with or without CXCL15 mutation, as well as on macrophages within the TME and spleen tissues.** G** Immunofluorescence analysis of CD206, CD36, 15d-PGJ2, maresin-1, CXCL15, CXCR2, and CD47 expression in the TME of RM-1/CXCR2 tumour-bearing C57BL/6 mouse models. **H** Isolation of splenic macrophages from NSG mice cocultured with different tumour cells or supplemented with various lipids, followed by Western blot analysis to assess activation of the PPAR-γ pathway. The data are presented as the means ±SDs of at least three replicates. NS indicates no significance, * indicates *p* < 0.05, ** indicates *p* < 0.01, and *** indicates *p* < 0.001; *p* < 0.05 was considered statistically significant

Finally, in C57BL/6 model mice implanted with RM-1/CXCR2 tumour cells, TAMs exhibited prominent M2 polarization upon CXCL15 activation. This effect was abolished in CXCL15 knockout mice with CD36 inhibition or when tumour cells lacked CXCR2 expression (Fig. 6A–B). Collectively, these findings delineate a CXCL15/CXCR2–ω−3/6 PUFA–CD36 signalling axis that drives M2 polarization in TAMs. However, further investigations are warranted to elucidate the downstream regulatory mechanisms involved in the uptake of ω−3/6 VLC-PUFAs by macrophages.

### Lipids taken up via CD36 can activate the PPAR-γ pathway in Macrophages and regulate M2 polarization

Our previous study demonstrated that the IL-8/CXCR2 pathway regulates the AKT–mTORC1–FASN/mTORC2–ELOVL5 axis, leading to the accumulation of free fatty acids (FFAs) and VLC-PUFAs in the TME [6]. To investigate the source of ω−3/6 PUFAs in the TME, we utilized the RM-1/CXCR2 cell line model and confirmed this phenomenon (Fig. 6C). In a corresponding mouse tumour model established with RM-1/CXCR2 cells, tumour tissues were harvested for analysis after tumour development (Fig. 6D). The results revealed elevated expression of CXCR2, CXCL15, FAS, ELOVL5, and CD47 (Supplemental Figs. 8 A–B). Immunofluorescence analysis confirmed the coexpression of CXCR2, CXCL15, FAS, and ELOVL5 within the tumours (Fig. 6E). Oil Red O staining revealed that the tumour cells secreted abundant lipids, which were subsequently taken up by macrophages. However, in CXCL15-mutant mice, tumour cells failed to secrete sufficient lipids into the TME, and compared with TAMs within the TME, splenic macrophages exhibited reduced lipid uptake (Fig. 6F). The ω−6 PUFA metabolite arachidonic acid (20:4) is taken up by macrophages via CD36 and further metabolized into 15-deoxy-Δ12,14-prostaglandin J2 (15d-PGJ2) [21]. Upon nuclear entry, 15d-PGJ2 activates the PPAR-γ signalling pathway, promoting M2 macrophage polarization [21]. Similarly, the ω−3 PUFA metabolite docosahexaenoic acid (DHA, 22:6) is metabolized by macrophages into resolvin D1/D2 and maresin-1, which also activate nuclear PPAR-γ and induce M2 polarization [22]. Our study revealed that maresin-1 and 15d-PGJ2 colocalized with CD36 in M2 macrophages, which predominantly infiltrated areas enriched for CXCL15, CXCR2, and CD47 expression (Fig. 6G). Immunohistochemistry staining further confirmed the high expression of maresin-1 and 15d-PGJ2 in tumours from RM-1/CXCR2-bearing mice, with downregulation observed following CXCL15 mutation (Supplemental Figs. 8 C–D). To functionally validate these findings, we isolated splenic macrophages from NSG mice and cocultured them with different tumour cells or supplemented the culture media with various lipids. Western blot analysis of total protein and nuclear protein revealed that both the IL-8/CXCR2 pathway and ω−3/6 PUFAs promoted M2 polarization via activation of the PPAR-γ signalling pathway (Fig. 6H & Supplemental Fig. 8E).

### Validation of the therapeutic efficacy of the CXCR2 blocker in both the NSG and C57BL/6 mouse models

**Fig. 7:**
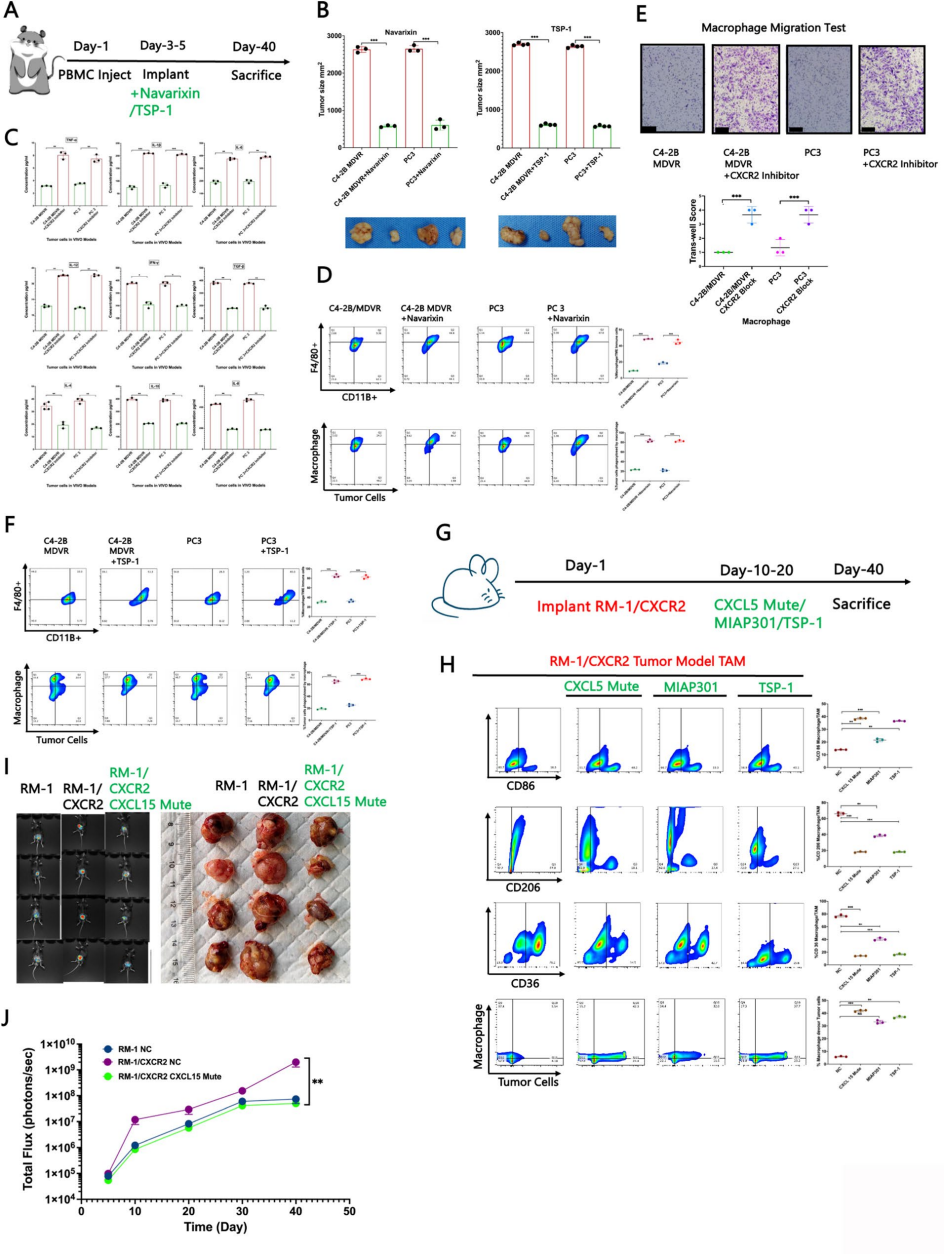
(**A**-**B**) Mouse experiment diagram drawing and tumour size in tumour-bearing mouse models after treatment with navarixin/TSP-1; data points represent biological replicates derived from three individual mice, and each experiment was independently repeated three times to ensure reproducibility. **C **Cell factors secreted by tumour-associated macrophages after CXCR2 blockade were assessed by ELISA; data points represent biological replicates derived from three individual mice, and each experiment was independently repeated three times to ensure reproducibility. **D **Tumour-associated macrophages (CD11b + F4/80+) were assessed by flow cytometry from tumours collected from mice bearing tumours constructed from different cell lines after treatment with navarixin; the ability of tumour-associated macrophages to engulf tumour cells after CXCR2 blockade was assessed by flow cytometry, with data points representing biological replicates derived from three individual mice and each experiment independently repeated three times to ensure reproducibility. **E **Macrophage migration test by the Transwell method in several cell Lines, with the black bars in the images representing 200 μm; in this cell experiment, each experiment was independently repeated three times to ensure reproducibility. **F **Tumour-associated macrophages (CD11b + F4/80+) identified by flow cytometry from tumours collected from mice bearing tumours constructed from different cell lines after TSP-1 treatment; the ability of tumour-associated macrophages to engulf tumour cells after TSP-1 treatment was assessed by flow cytometry, with data points representing biological replicates derived from three individual mice and each experiment independently repeated three times to ensure reproducibility. **G **C57BL/6 mouse experiment diagram drawing. **H **The comparative effects of TSP-1, CD47 blockade and CXCL15/CXCR2 blockade therapies on macrophage polarization; data points represent biological replicates derived from three individual mice, and each experiment was independently repeated three times to ensure reproducibility;. **I-J **Tumour size and tumour growth curve after the CXCR2 blockade in C57BL/6 mice. The data are presented as means ±SDs of at least three replicates. NS indicates no significance, * indicates *p* < 0.05, ** indicates *p* < 0.01, and *** indicates *p* < 0.001; *p* < 0.05 was considered statistically significant

To evaluate the therapeutic potential of targeting the CXCR2 axis, we conducted in vivo experiments using NSG mouse models. Compared with untreated controls, tumour-bearing mice treated with navarixin—an orally bioavailable small-molecule antagonist of CXCR1/2—or with thrombospondin-1 (TSP-1), which simultaneously blocks CD47 on tumour cells and CD36 on macrophages, presented significantly reduced tumour volumes (Fig. 7A–B). Notably, the antitumour effects of TSP-1 treatment were comparable to those achieved with navarixin (Fig. 7A–B). Furthermore, cytokine profiling following CXCR2 inhibition revealed a substantial decrease in M2-associated cytokines alongside an increase in M1-associated cytokines, indicating a shift towards a proinflammatory macrophage phenotype (Fig. 7C). Flow cytometry further revealed an overall increase in the population of TAMs in tumours from mice treated with CXCR2 inhibitors (Fig. 7D). However, the phagocytic activity of TAMs towards tumour cells was impaired after CXCR2 blockade (Fig. 7D–E). Importantly, navarixin treatment also resulted in increased Ki67 expression in CD8⁺ tumour-infiltrating lymphocytes (TILs) and promoted M1 macrophage differentiation. Concurrently, a significant reduction in Tregs and M2 macrophages was observed (Supplemental Fig. 9 A). Similarly, TSP-1 treatment restored macrophage infiltration and increased the phagocytic activity of macrophages towards tumour cells (Fig. 7F). It also promoted macrophage migration (Supplemental Fig. 9B) and shifted the macrophage phenotype towards the M1 phenotype, as evidenced by increased M1 cytokine levels and reduced M2 cytokine levels in CXCL15-mutant mice (Supplemental Fig. 9 C). Parallel changes in Ki67 expression in tumour-infiltrating CD8⁺ T cells and infiltration of macrophages were observed in CXCL15-deficient mice, further supporting the regulatory role of the CXCL15/CXCR2 axis (Supplemental Fig. 9D). Comparable results were obtained in the C57BL/6 mouse model (Fig. 7G). Notably, blocking CXCL15 alone yielded superior therapeutic outcomes compared with CD47 inhibition via MIAP301, more effectively reversing TAM M2 polarization and restoring antitumour macrophage function (Fig. 7H). Overall, our findings demonstrate that inhibition of the CXCL15/CXCR2 signalling axis significantly suppresses tumour progression and elicits a robust antitumour immune response in preclinical models (Fig. 7I–J).

## Discussion

The interaction between the immune system and the TME plays a pivotal role in cancer progression and metastasis [23]. Among the various cytokines and chemokines present in the TME, IL-8 and its receptor CXCR2 have drawn considerable attention for their roles in prostate cancer biology [24–27]. The IL-8/CXCR2 axis is frequently upregulated across multiple malignancies and is associated with poor prognosis and increased tumour aggressiveness [27]– [7]. IL-8 promotes inflammation by recruiting immune cells while also shaping an immunosuppressive microenvironment that enables tumours to evade immune surveillance [27]. Notably, the IL-8/CXCR2 pathway regulates CD47 expression, revealing a key connection between inflammatory signalling and immune checkpoint modulation [7]. Our findings further demonstrate that IL-8 signalling through CXCR2 upregulates CD47 expression in tumour cells, potentially influencing macrophage behaviour within the TME.

CD47, an integrin-associated protein (IAP), is broadly expressed on the surface of both haematopoietic and nonhematopoietic stem cells and functions as a well-known “do not eat me” signal [8]– [9, 28–30]. As a transmembrane protein, CD47 interacts with signal regulatory protein alpha (SIRPα) on macrophages, thereby inhibiting phagocytosis and contributing to immune evasion—a mechanism that has garnered increasing interest in cancer research [18]. In tumours, elevated CD47 expression is commonly associated with impaired immune clearance of cancer cells by macrophages [8]– [9, 31]. While normal cells use CD47 expression to avoid unintended phagocytosis [32], cancer cells exploit this pathway by overexpressing CD47 to evade immune detection and promote malignant progression [33]– [34]. Crosstalk between prostate cancer cells and M2-polarized macrophages has also been reported, further supporting the role of immune suppression in disease progression [35]. In our study, we propose that IL-8/CXCR2 blockade reduces CD47 expression, which renders tumour cells more vulnerable to macrophage-mediated phagocytosis and increases antitumour immunity. This finding aligns with emerging therapeutic approaches targeting CD47 to increase macrophage infiltration and activity within tumours [8]– [9]. Given the central role of macrophages in the TME and their impact on tumour progression [36], the interplay between IL-8/CXCR2 signalling, CD47 regulation, and macrophage infiltration holds significant clinical relevance.

Additionally, emerging evidence suggests that lipid accumulation contributes to macrophage-regulated prostate cancer aggressiveness [37]– [38]. Prostate cancer cells can shift their metabolic preference from glycolysis to a FAO-centred pathway, which not only sustains the high energy demand required for aggressive tumour growth but also fortifies these cells against macrophage-mediated phagocytosis. This metabolic reprogramming, coupled with CD47-mediated immune evasion, provides a dual survival advantage for therapy-resistant and recurrent cancer cells [39–41]. Notably, recent studies—including our own—indicate that CD47 expression can be induced by AC-CoA, a key metabolic intermediate, and that glycolysis itself may serve as a source of AC-CoA in tumour cells [39–41]. To investigate this further, we conducted experiments to explore how IL-8/CXCR2 signalling modulates cellular metabolism and, in turn, promotes CD47 expression. Importantly, our findings reveal that IL-8/CXCR2 signalling regulates both the expression and membrane localization of CD47 in prostate cancer cells through palmitoylation, a lipid modification process. This mechanism provides a compelling explanation for how the IL-8/CXCR2 axis increases CD47 surface expression and enables tumour cells to evade macrophage-mediated clearance. Furthermore, CXCR2 expression in NEPC cells is known to stimulate the secretion of proangiogenic factors, thereby promoting angiogenesis [24]. It also contributes to the development of the NE phenotype and therapeutic resistance [24]. Thus, targeting CXCR2 could not only reverse CD47/SIRPα-mediated resistance to phagocytosis and suppress M2 macrophage polarization but also counteract NED in prostate cancer cells. Together, these findings underscore the multifaceted therapeutic potential of CXCR2 inhibition in overcoming immune evasion, metabolic reprogramming, and treatment resistance in prostate cancer.

Furthermore, the interplay between IL-8/CXCR2 signalling, CD47 regulation, and macrophage infiltration has significant clinical implications in prostate cancer [42]– [43]. M1 and M2 macrophages perform distinct functions within the TME [44]. A higher M1/M2 macrophage ratio is generally associated with improved prognosis and better responses to therapy [44–46]. M1 macrophages produce proinflammatory cytokines such as TNF-α and IL-12, which support antitumour immunity, whereas M2 macrophages secrete immunosuppressive cytokines such as IL-10 and TGF-β, which promote tumour growth and immune tolerance [45].

In prostate cancer, the balance between M1 and M2 macrophage infiltration has been shown to influence tumour progression and clinical outcomes [45]. Our findings suggest that targeting the IL-8/CXCR2 axis may shift macrophage polarization from the immunosuppressive M2 phenotype towards the proinflammatory M1 phenotype, thereby increasing antitumour immune responses. Conversely, an enrichment of M2 macrophages is linked to immune evasion, metastasis, and poor prognosis because of their ability to suppress immune responses and promote tumour angiogenesis and migration [44–46]. Mechanistically, we observed that IL-8/CXCR2 signalling promotes prostate cancer cells to secrete ω−3/6 PUFAs, which are taken up by macrophages via CD36 and contribute to M2 polarization, thus fostering an immunosuppressive TME. Interestingly, TSP-1 has been shown to bind both CD36 and CD47, inhibiting tumour growth and modulating the TME [42]– [43]. Although the therapeutic potential of TSP-1 in prostate cancer remains underexplored, some clinical studies support its antitumour effects [41–43]. Our data demonstrate that CXCR2 inhibition achieves immunotherapeutic effects comparable with those of TSP-1, further supporting its promise as a therapeutic target. Collectively, these findings highlight the link between macrophage polarization and clinical outcomes in prostate cancer, suggesting that combining IL-8/CXCR2 inhibition with existing immunotherapies may offer synergistic benefits for patients.

While our study focused primarily on macrophage reprogramming via CXCR2 signalling, additional evidence from our ongoing and parallel work suggests that IL-8/CXCR2 blockade may also affect CD8⁺ T cells, Tregs, and NK cells. Specifically, CXCR2 activation leads to increased lipid enrichment in the TME, which promotes CD8⁺ T-cell ferroptosis and increases Treg infiltration [6]. Moreover, CXCR2 inhibition has been shown to increase NK cell infiltration in tumours, which is consistent with other findings in the field [47]. These insights broaden the understanding of the immunological impact of CXCR2 signalling and support further investigations into its role in modulating both innate and adaptive immunity.

Targeting the IL-8/CXCR2 signalling axis represents a compelling strategy to increase antitumour immunity. Inhibition of this pathway suppresses CD47 membrane localization on tumour cells, thereby attenuating the “do not eat me” signal and restoring macrophage-mediated phagocytosis. Additionally, IL-8/CXCR2 blockade reduces the secretion and macrophage uptake of immunosuppressive lipids—such as ω−3/6 polyunsaturated fatty acids—thereby limiting M2 macrophage polarization and promoting a shift towards a more immunostimulatory tumour microenvironment. When combined with current immunotherapies, including anti-PD-1- or CD47-targeting agents, IL-8/CXCR2 inhibition may potentiate both innate and adaptive immune responses, ultimately increasing therapeutic efficacy. Our findings identify IL-8/CXCR2 as a druggable upstream regulator of immune‒metabolic crosstalk and a promising therapeutic target for restoring antitumour immunity in NEPC. This dual role positions CXCR2 as an attractive target for combination therapies. In particular, cotargeting CXCR2 with immune checkpoint inhibitors—such as anti-CD47 or anti-PD-1/PD-L1 antibodies—may increase therapeutic efficacy.

Despite these promising findings, our study has certain limitations that warrant further investigation. For example, a deeper exploration of macrophage heterogeneity within the tumour microenvironment could provide insights into the diverse roles and plasticity of these immune cells, thereby refining macrophage-targeted strategies. Furthermore, investigating the crosstalk between IL-8/CXCR2 signalling and other immune checkpoint pathways may reveal additional regulatory mechanisms with therapeutic potential. Emerging studies have shown that CXCR2 blockade can synergize with ICIs to overcome resistance in immunosuppressive tumours [48]– [49]. For example, in rhabdomyosarcoma, CXCR2 inhibition reduces MDSC recruitment, a key barrier to the PD-1 response [49]. The core mechanism of IFN-I → ATP → DC/T-cell activation proposed in the study by Zhou et al., combined with our previous findings on IFN pathway-mediated PD-L1 upregulation and Treg infiltration, provides a theoretical foundation for CD47 + CTLA-4 combination therapy [50]. Additionally, CXCR2 inhibition has been linked to decreased CD47 expression (as demonstrated in our data), whereas CD47 blockade directly increases macrophage phagocytosis. Dual targeting of this axis has shown promise in models such as pancreatic cancer models, in which combining CD47 inhibition with necroptosis inducers significantly curtailed metastasis [51]. Future studies should further explore the role of metabolic cues—such as VLC-PUFA depletion downstream of CXCR2—as predictive biomarkers for therapeutic synergy. Moreover, our study focused primarily on the role of IL-8/CXCR2; however, other S-palmitoyltransferases, such as ZDHHC5 and ZDHHC17, may also regulate CD47 function in a context-dependent manner [52]. ZDHHC5, which palmitoylates integrins and EGFR [52], can indirectly stabilize CD47 by modulating its interaction partners in the plasma membrane. ZDHHC17, known for its role in neuronal protein trafficking, might similarly influence CD47 localization in neuroinflammatory or brain tumour microenvironments [52]. Future studies should systematically profile the palmitoyltransferase ‘landscape’ governing CD47 modification across cancer types, as combinatorial inhibition of multiple ZDHHCs may increase the efficacy of CD47-targeted therapies.

## Conclusion

Our findings reveal that the IL-8/CXCR2 pathway plays a crucial role in regulating CD47 expression and TAM polarization within the TME. Targeting this signalling axis may increase antitumour immunity and offer a novel therapeutic strategy for improving the outcomes of cancer immunotherapy.

## Experimental model and method details

### Patients and samples

Human tissue samples were obtained from twenty prostate cancer patients who underwent surgical resection at Sichuan People’s Hospital between 2021 and 2024. Adenocarcinoma samples were collected from patients undergoing robot-assisted radical prostatectomy (RRPC). Castration-resistant prostate cancer (CRPC) samples were obtained from patients with primary prostate adenocarcinoma who initially received hormonal therapy instead of surgery and eventually experienced tumour recurrence, classified as CRPC, leading to urinary obstruction, for which transurethral resection of the prostate was performed. Neuroendocrine prostate cancer (NEPC) samples were obtained from patients whose primary prostate NEPC was located within in situ prostate tissue. Each group included 9 samples, with average patient ages of 71 ± 3.2 years (BPH), 72 ± 1.2 years (hormone-sensitive adenocarcinoma), 74 ± 3.1 years (CRPC), and 70 ± 2.2 years (NEPC). Androgen receptor (AR) expression was notably present in the BPH, hormone-sensitive adenocarcinoma, and CRPC groups, whereas only 1 patient in the NEPC group exhibited low AR expression. All patients provided written informed consent prior to sampling, in accordance with the Declaration of Helsinki.

### Cell lines and reagents

The benign prostate cell line BPH-1 and prostate cancer cell lines LNCaP, C4-2, CWRR-1, PC3, DU145, LASCPC-01, and NCI-H660 were obtained from ATCC. Although LNCaP cells are metastatic in origin, they are widely recognized as androgen-dependent prostate cancer cells and, in this study, served as a model for studying androgen-sensitive prostate cancer [46]. NCI-H660 and LASCPC-01 cells were cultured in HITES medium, and DU145 cells were cultured in DMEM, while LNCaP, C4-2, CWRR-1, and PC3 cells were maintained in RPMI medium supplemented with 10% foetal bovine serum (FBS) and 1% penicillin. BPH-1 cells were cultured in RPMI medium supplemented with 20% FBS and 1% penicillin. Cell lines with overexpression or knockdown of specific genes, including C4-2B/CXCR2 overexpression (OE), LNCaP/CXCR2 OE, C4-2B/IL-8 OE, PC3/CXCR2 knockdown (KD), and PC3/CD47 KD, were generated by Hanbio Tech (Shanghai, China). shRNA targeting CD47 or CXCR2 was subcloned and inserted into the GFP-positive lentiviral vector FG12 (Addgene) to create FG12-shFASN/CXCR2, with scrambled shRNA used for control vectors. These modified cell Lines were cultured in RPMI medium supplemented with 10% FBS and 1% penicillin. Additionally, the C4-2B/MDVR and LNCaP/MDVR cell lines were derived from C4-2B and LNCaP cells and cultured in the presence of 10 µM enzalutamide. To investigate the effects of various treatments, the cells were exposed to 100 ng/mL human interleukin-8 (hIL-8; Hanbio Tech), 50 µM WZB117 (PDC-E2 inhibitor; Hanbio Tech), 50 µM etomoxir (CPT-1 A inhibitor; Hanbio Tech), or 10 µg/mL B6H12 (CD47 inhibitor; Hanbio Tech). For Lipid impact studies, cells were cultured in RPMI medium without FBS, supplemented with 1 mM palmitate/acetyl-CoA (AC-CoA) as needed, or, alternatively, in RPMI medium containing Lipid-depleted FBS. All the cell Lines were incubated at 37 °C in a 5% CO₂ atmosphere. Some cells were harvested for flow cytometry analysis experiments, while for others, supernatants were collected following cell culture.

Humans cannot synthesize PUFAs with multiple double bonds de novo; ω−6 and ω−3 PUFAs (mainly α-linolenic acid and linoleic acid) must be obtained from dietary sources and elongated to form VLC-PUFAs with more than 20 carbon atoms and multiple double bonds [53]. Because standard culture media contain limited amounts of these essential fatty acids, we supplemented the media with α-linolenic acid and linoleic acid in our cell culture experiments and analysed their metabolic profiles and gene expression.

The RM-1 murine prostate cancer cell Line was cultured under standard conditions to ensure optimal growth and viability. The cells were maintained in high-glucose DMEM supplemented with 10% heat-inactivated FBS, 1% penicillin‒streptomycin (100 U/mL), and optionally with 2 mM L-PDC-E2amine or PDC-E2aMAX™ to support metabolic activity. The cells were incubated at 37 °C with 5% CO₂ and 95% humidity to prevent medium evaporation. Murine CXCL15 was added at a final concentration of 50 ng/mL as needed. The FA6-152 antibody at a final concentration of 10 µg/mL was selected for in vitro experiments to investigate the role of CD36 in regulating immune cell lipid uptake.

### Xenograft animal models

Animal care and experiments were conducted in accordance with the guidelines of the Animal Committee of the National Cancer Center and were approved by the Ethics Review Committee for Animal Experimentation of the National Cancer Center. Immunocompromised NSG (NOD.Cg-Prkdc^scid Il2rg^null/SzJ) and nude (nu/nu) mice were obtained from SPF (Beijing) Biotechnology (Beijing, China). The required sample size was estimated with GraphPad. Before randomization, the animals were labelled with unique identifiers using ear tags, and group assignments were made with Excel, eliminating human bias in group allocation. One researcher implemented the experimental protocol, while another analysed the data, with both blinded to the group assignments. The principal investigator later combined the analysed data with group assignments to summarize the results, ensuring investigator blinding During the execution and analysis of the study. Initially, 3× 10⁴ PBMCs were injected via the tail vein into 3- to 6-week-old male NSG mice. After 3 to 5 days, a total of 2 × 10⁶ cells from various cell Lines were suspended in 0.1 mL of 1× RPMI 1640 medium supplemented with 10% FBS and 50% Matrigel (Corning) and then subcutaneously inoculated into the bilateral flanks of the mice. The mice were sacrificed either when the tumour xenografts reached 1000 mm³ (tumour volume calculated as volume (mm³) = length×height²/2) or 40 days after the experiment began. In certain experimental groups, 80 ng of hIL-8 in 200 µL of Iscove’s modified Dulbecco’s medium (IMDM; Life Technologies, Grand Island, NY) was administered once daily for two consecutive days, whereas the control group received PBS. To assess metabolic changes following blockade of the CXCR2‒CD47 pathway, navarixin (CXCR2 inhibitor) and CC-90,002 (CD47 inhibitor) were administered intraperitoneally at 50 mg/kg and 20 mg/kg weekly, respectively. Tumour samples and serum were collected three weeks after tumour cell injection for tumour-infiltrating lymphocyte (TIL) analysis and ELISA tests. Tumours were also harvested for metabolomics mass spectrometry, with spleen samples collected as controls. Tumour sizes were recorded throughout the study. This research was approved by the Ethics Committee of the First Affiliated Hospital of Guangzhou Medical University.

### Peripheral blood mononuclear cell culture models

Peripheral blood mononuclear cells (PBMCs) were isolated from donor whole blood samples by density gradient centrifugation and treated with IL-2, M-CSF, and GM-CSF to generate tumour-associated macrophages. Palmitate (Nacalai Tesque, Kyoto, Japan) or acetyl-CoA (Huabio, China) was dissolved in 100% ethanol at a concentration of 200 mM and conjugated to fatty acid-free bovine serum albumin (BSA) at a 5:1 molar ratio, resulting in a final concentration of 8 mM palmitate-BSA or acetyl-CoA–BSA. This mixture was prepared by vortexing and sonicating at 37 °C for 3–4 h. A total of 1 × 10⁵ whole PBMCs or 1 × 10⁴ sorted T-cell subsets, in the presence of 1 × 10⁵ irradiated antigen-presenting cells (APCs), were stimulated with an anti-CD3 monoclonal antibody (clone: OKT3) and an anti-CD28 monoclonal antibody (clone: CD28.2). The cells were cultured in glucose-free RPMI medium (Thermo Fisher Scientific) supplemented with a 10% lipid solution without FBS (Biowest, Nuaillé, France), 10 IU/mL IL-2, 20 ng/mL IL-7, 1 mM glucose, and the indicated concentrations of palmitate–BSA or acetyl-CoA–BSA. Additionally, PBMCs were cultured in supernatant collected from the medium used for tumour cell culture. Although the data presented in this manuscript correspond to individual donors, during the implementation of each experiment, it was ensured that PBMCs from the same donor were used, thereby negating the need for statistical comparison across groups. All the donors were healthy individuals.

### C57BL/6 mouse experiments and RM-1 cell inoculation

To construct the RM-1/CXCR2 cell line, RM-1 cells were transduced with a CXCR2-overexpressing lentivirus and selected with puromycin, and stable clones were verified by qPCR, Western blotting, and flow cytometry for CXCR2 expression. C57BL/6 male mice (6–8 weeks old) were housed under specific pathogen-free (SPF) conditions with a 12-hour light/dark cycle, controlled temperature (22–25 °C), and free access to food and water. Before tumour cell implantation, the mice were acclimated for one week to minimize stress-related variability.

CXCL15 mutant mice were generated by electroporating C57BL/6J zygotes with a mixture containing 50 ng/µL Cas9 protein (Millipore Sigma), 0.6 pmol/µL each crRNA [spacer sequence GCTGAGCCTTCTACCTGGGA; NCBI GRCm38.4 Chr5:90213456–90213475(+)] and tracrRNA (Millipore Sigma), along with 50 ng/µL ssODN donor (50GCTGAGCCTTCTACCTGGGAAGTGAAGGCTGCCATTGCTCCAGTGGATGCTGGAAGCCTTCTTGCAGTGCTGAGGGTGGAACCTCCCGGAAGGTGCAAGTGAAGCCTGGAAGcgtACCCAGGTAGAAGGCTCAGC, lower case indicates sequence modification; Integrated DNA Technologies, Ultramer). The electroporated zygotes were transferred the same day into pseudopregnant mice, which were allowed to give birth to potential founders. Sanger sequencing identified three founder mice carrying the intended CXCL15 mutation. The PCR primers used for genotyping were as follows: CXCL15-F: 5’-TGGCATCTGAGGAAGACACC-3’; and CXCL15-R: 5’-CAGGGAAGTCTGCTGCTGTA-3’.

Luciferase-expressing RM-1-Luc and RM-1/CXCR2-Luc cells were cultured under standard conditions (described below), harvested at 80% confluence, and resuspended in phosphate-buffered saline (PBS) or serum-free medium at a concentration of 5 × 10⁶ cells/mL. Each mouse was anaesthetized with isoflurane or intraperitoneal ketamine/xylazine, and 100 µL of RM-1-Luc cells and RM-1/CXCR2-Luc cell suspensions (5 × 10⁵ cells per mouse) were injected orthotopically into the dorsal prostate region. Tumour progression was monitored in vivo by bioluminescence imaging (BLI) with an IVIS imaging system. The mice were injected intraperitoneally with D-luciferin (150 mg/kg, 10 min before imaging), and fluorescence signals were captured at different time points to assess tumour growth. The mice were also observed daily for body weight changes and overall health status. At the experimental endpoint, the mice were euthanized, and the tumours were harvested for histological analysis, flow cytometry, and molecular assays.

### Immunohistochemistry and Immunofluorescence staining of tissue samples

For immunohistochemistry (IHC) staining preparation, all sections, including target sample slides, positive control slides, and negative control slides, were deparaffinized, rehydrated, and subjected to antigen retrieval by boiling in citrate buffer (pH 6.0) for 40 min in a water bath prior to antibody staining. The slides were then incubated with primary antibodies optimized for dilution for 1 h at room temperature. Horseradish peroxidase (HRP)-conjugated secondary antibodies (Dako EnVision + Kit) were applied for 30 min, followed by visualization with diaminobenzidine (DAB) after a 30-minute incubation at room temperature. For immunofluorescence, after being washed with PBS, the sections were incubated with secondary antibodies—donkey anti-rabbit (H + L) Alexa Fluor 532 (Invitrogen), donkey anti-mouse (H + L) Alexa Fluor 594 (Invitrogen), and donkey anti-goat (H + L) Alexa Fluor 488 (Invitrogen)—for 90 min at room temperature and counterstained with DAPI (Sigma‒Aldrich). A complete List of antibodies is provided in Supplemental File 1.

### Cell Immunofluorescence

The cells were seeded the day prior to fixation at a density of 8 × 10³ cells per well. The cells were washed twice with PBS (14190-094; Gibco) and fixed with 4% paraformaldehyde (PFA) for 15 min. Following fixation, the cells were washed three times with PBS, permeabilized with 0.1% Triton X-100 in PBS for 20 min, and blocked with 3% BSA in PBS for 30 min. The cells were then incubated with primary antibodies diluted in 1% BSA in PBS for 1.5 h at 37 °C. After primary antibody incubation, the cells were washed three times with PBS, followed by the addition of secondary antibodies (anti-mouse Alexa 488, Invitrogen) diluted in PBS and Hoechst stain (1:500) for 30 min at 37 °C in the dark. After incubation, the cells were washed three times with PBS and stored in PBS at 4 °C until imaging. A complete List of antibodies is provided in Supplemental File 1.

### Western blotting and ELISA analyses

After culturing, the cells were lysed, and proteins were extracted with RIPA buffer in accordance with the manufacturer’s instructions. The extracted proteins were separated by 10% sodium dodecyl sulfate‒polyacrylamide gel electrophoresis (SDS‒PAGE). The protein bands were visualized by the chemiluminescence method (ECL Plus Western blot Detection System; Amersham Biosciences, Foster City, CA, USA). The concentrations of human cytokines were quantified by specific sandwich ELISAs according to the manufacturer’s protocols (Elabscience, Wuhan, China). A complete List of kits and antibodies is provided in Supplemental File 1.

### THP-1 phagocytosis assay

In vitro phagocytosis assays were conducted using human THP-1 monocyte cells, which were differentiated into macrophages by incubation with 40 nM phorbol 12-myristate 13-acetate (PMA) for 48 h. Tumour cell lines were stably transfected with GFP. GFP-expressing tumour cells (1–1.5 × 10⁶) were cocultured with macrophages (1 × 10⁶) in a six-well plate for 2–4 h at 37 °C. After coculture, the cells were collected and washed with 0.5% BSA-PBS. The cell pellets were incubated in the dark with an APC/cyanine 7-labelled CD11b primary antibody (catalogue #101225, BioLegend) for 30 min, followed by three washes with 0.5% BSA-PBS. Phagocytic activity was analysed via flow cytometry (Becton Dickinson Canto II, BD, NJ, USA) and FlowJo software (Three Star, Inc., Ashland, OR, USA).

### Real-time PCR

Total RNA was extracted with TRIzol reagent, and cDNA was synthesized with the iScript™ cDNA Synthesis Kit (Bio-Rad, Richmond, CA, USA). GAPDH was used as the housekeeping gene and served as an internal control. PCR amplification was performed with SYBR Premix Ex Taq II (TAKARA, Dalian, Liaoning, China) according to the manufacturer’s instructions. The reactions were carried out on a Bio-Rad iQ5 thermal cycler (Hercules, CA, USA), and the quality of the PCR products was assessed through post-PCR melting curve analysis.

### Liquid chromatography‒mass spectrometry assay for free fatty acid species

A liquid chromatography‒mass spectrometry (LC‒MS) assay was used to measure the concentrations of fatty acid (FA) species in the culture medium. LC was performed with an LC-20ADXR ternary pump system equipped with a DGU-20A5R degassing unit, a SIL-20AC autosampler, and a CTO-20AC column oven (Shimadzu Co., Ltd., Kyoto, Japan). The LC system was coupled to an LTQ Orbitrap XL hybrid linear ion trap–Fourier transform mass spectrometer (Thermo Fisher Scientific). Fatty acids were detected by extracting the ion chromatograms of deprotonated ions ([M-H]⁻) with a mass tolerance of 10 ppm. Instrument control, data acquisition, and processing were conducted with Xcalibur 2.1.0 software (Thermo Fisher Scientific).

### Flow cytometry analysis

Flow cytometry (FCM) staining and analysis were performed as previously described [5]. The antibodies used for FCM analyses are detailed in Supplemental File 1. The cells were washed with a wash solution, stained with surface antibodies, and treated with a fixable viability dye (Thermo Fisher Scientific). Intracellular staining was subsequently performed using intracellular antibodies and various Staining Buffer Sets (Thermo Fisher Scientific) following the manufacturer’s instructions. After washing, the cells were analysed with an LSR Fortessa or Symphony instrument (BD Biosciences) and FlowJo software (BD Biosciences). We utilized flow cytometry to isolate target cell types, and the gating process involved the following parameters. For initial gating, forwards scatter (FSC) and side scatter (SSC) were used to exclude debris and select live, single cells, and dead cells were excluded with viability dyes. For lineage markers, for immune cell isolation, lineage-specific markers such as CD45 for leukocytes, were used; for specific cell types, CD8 + T cells were gated as CD45 + CD3 + CD8+, Tregs were gated as CD45 + CD3 + CD4 + CD25 + FOXP3+, and macrophages were gated as CD45 + F4/80 + CD11b+. The gating of positive populations was determined with fluorescence minus one (FMO) controls for each marker, and unstained cells were used to define background fluorescence levels.

### Apoptosis and proliferation analyses

Apoptosis was evaluated by FCM with FITC-annexin V, 7-AAD, Phen Green SK, CM-H2DCFDA, propidium iodide, and active caspase-3 staining (Thermo Fisher Scientific). The staining reagents were diluted according to the manufacturer’s instructions. Cell proliferation was assessed by FCM on the basis of the dilution of Ki67-labelled cells (Thermo Fisher Scientific). All the staining reagents were prepared according to the manufacturer’s protocols.

### Transwell invasion assay

A Transwell invasion assay was performed to evaluate the migratory ability of macrophages towards different cell lines. Matrigel (BD Biosciences) was diluted in coating buffer (0.01 M Tris, pH 8.0, 0.7% NaCl) to a final concentration of 200–300 µg/ml (1:40–1:45 dilution from stock). A total of 100 µl of the diluted Matrigel was applied to the upper chamber of a 24-well Transwell insert (Corning, NY) and incubated at 37 °C for 2 h to allow gelling. After the coating buffer was removed from the permeable support membrane, the test cells were resuspended in medium containing 1% FBS at a density of 2.5 × 10⁴ cells/300 µl and added to the upper chamber. The lower chamber was filled with 800 µl of fresh culture medium containing 5 µg/ml fibronectin (Santa Cruz, USA). The cells were incubated for 48 h, and the cell invasion capacity was assessed by staining the membrane with a Diff-Quick Stain Kit (K7128, IMEB INC).

### Principles of the transwell score

The Transwell staining results were assigned as the mean score considering the proportion of stain. This measure is also known as the frequency, defined as follows: 0 for less than 5%, 1 for less than 25%, 2 for 26%−50%, 3 for 51%−75%, and 4 for greater than 75%.

### Oil red O staining and cell lipid uptake fluorescence assay

To assess Lipid accumulation in tissues, Oil Red O staining was utilized. Frozen tissue slides were incubated for 12 h, followed by treatment with 250 µM free fatty acid (FFA) oleate (oleic acid: palmitic acid = 2:1) for 24 h. The cells were rinsed twice with PBS and fixed with 4% paraformaldehyde for 30 min at room temperature, followed by two washes with deionized water (dH2O). Approximately 50 µl of Oil Red O working solution (prepared by mixing Oil Red O stock solution with dH2O at a 6:4 ratio) was applied in the dark, and the slides were incubated for 15 min at room temperature. The Oil Red O solution was removed, and 50 µl of 60% isopropanol was added to each well for 20 s at room temperature. The slides were washed 2–5 times with dH2O until all excess stain was completely removed. The dye was eluted with 100 µl of DMSO and incubated for 10 min with gentle shaking. Lipid accumulation was quantified with a fluorescence microplate spectrophotometer (Molecular Devices) at 510 nm, and images were captured with a Nikon microscope (Eclipse, E1000M, Japan). IHC staining analyses were performed on slides from the same sample, with representative views captured.

For the cell lipid uptake fluorescence assay, the cells of interest were seeded in culture plates and allowed to grow to the desired confluence. The cells were incubated with the lipid-specific fluorescent dye BODIPY. After incubation, the cells were washed with PBS to remove any excess dye or unincorporated lipids. If necessary, the cells were fixed with paraformaldehyde to preserve the cellular structure. A fluorescence microscope or flow cytometer was used to assess the fluorescence intensity, which indicates lipid uptake by the cells. The fluorescence signal was quantified to assess the level of lipid uptake by the cells.

### Gene expression data analysis

Differential gene expression analysis was performed on patient tissues and tumour tissues from mouse models, which were organized into multiple groups. Gene set enrichment analysis (GSEA) was conducted on the differentially expressed genes. The analysis referenced several databases, including the Kyoto Encyclopedia of Genes and Genomes (KEGG), Gene Ontology (GO) for Biological Process (BP), Molecular Function (MF), Lipidmap, Human Metabolome Database (HMDB), and Clusters of Orthologous Groups (COG). Hallmark analysis was also conducted, referencing publicly available databases.

### Luciferase reporter assay

Luciferase reporters (pGL2-basic-CD47) driven by the human CD47 promoter and the NF-κB promoter were constructed. The cells were seeded in triplicate in 96-well plates at a density of 8,000 cells per well and cotransfected with the PolIII–Renilla control reporter and the luciferase reporter using TurboFect transfection reagent (Thermo Scientific, USA, catalogue #R0533) for 48 h. After transfection, the cells were subjected to various treatments for different Durations. The cell lysates were prepared by adding 100 µl of lysis buffer (containing 1 mM DTT, 8 mM MgCl₂, 4 mM EGTA, and 100 nM PMSF), followed by a 15-minute incubation at room temperature. Subsequently, 10 µl of the lysate was mixed with 50 µl of luciferase buffer (containing 5 mM DTT, 100 nM ATP, 150 µg/ml AC-CoA, and 500 nM luciferin). Luciferase activity was measured with a Turner TD20/20 luminometer, with Renilla luciferase as an internal control to normalize the transfection efficiency.

### ^13^C-metabolite labelling

The cells were cultured in RPMI medium (11879-020, Thermo Fisher) supplemented with 11 mM ^13^C6-glucose (Cambridge Isotope Laboratories) and 10% dialyzed FBS. After 5 days, quenching and metabolite extraction were performed as previously described. Metabolite abundances and ^13^C-labelling patterns were analysed by gas chromatography–mass spectrometry (GC‒MS). Mass distribution vectors were extracted from the raw ion chromatograms with MATLAB and corrected for natural isotopes following the method of Fernandez et al. [54], and the fractional carbon contribution was calculated according to the method of Buescher et al. [55]. Palmitate uptake was measured with ^13^C16-labelled palmitic acid (Sigma‒Aldrich), and the difference in labelled palmitate in the medium after 5 days was normalized to the total protein content in each well. Fractional de novo fatty acid synthesis was determined by isotopomer spectral analysis following 5 days of exposure to ^13^C6-glucose. For histone extraction and SILAC analysis, 3D spheroids were collected after 5 days, and core histones were isolated via acid extraction. 4T1 cells cultured in SILAC DMEM (Silantes) supplemented with ^13^C615N4 L-arginine and ^13^C615N2 L-lysine (Cambridge Isotope Laboratories) served as internal histone standards (2:1 ratio). Histones were digested in 50 mM ammonium bicarbonate with Arg-C protease (Promega), and peptides were desalted with a C18 StageTip, resuspended in 1% trifluoroacetic acid (TFA) and 0.2% formic acid, and injected into an EASY-nLC system (Thermo Scientific) coupled with a mass spectrometry (MS) instrument. Peptides were separated on a 20-cm fused silica emitter (New Objective) packed with reverse-phase Reprosil Pur Basic 1.9 μm beads (Dr. Maisch) and eluted at a flow rate of 300 nL/min with a 60-minute Linear gradient from 5 to 30% buffer B (80% acetonitrile, 0.1% formic acid). The peptides were analysed by electrospray ionization using an Orbitrap Fusion Lumos (Thermo Scientific)by electrospray ionization. MS data were acquired with Xcalibur software (Thermo Scientific) and processed with MaxQuant (v.1.6.6.3) with a multiplicity of 2 and false discovery rates (FDRs) set to 1% for both proteins and peptides. Further data analysis was conducted with Perseus (v.1.6.2.2).

### PM protein extraction and subcellular fractionation

A Plasma Membrane Protein Extraction Kit (Abcam, ab65400) was used for the isolation of the plasma membrane (PM) and intracellular membrane (ICM) fractions. The cells were washed three times with PBS buffer and incubated at 4 °C in 2 mL of homogenized buffer. The cells were then homogenized 50 times and spun at 700 × g for 5 min after being harvested with a cell scraper. The supernatants were collected and centrifuged at 10,000 × g for 30 min at 4 °C. Total cellular membrane proteins were present in the pellet, while the cytosolic proteins were present in the supernatant. To separate the proteins in the PM and ICM, the pellet was resuspended in 200 µL of upper-phase solution. The mixture of upper-phase solution and isopyknic lower-phase solution was centrifuged at 1,000 × g for 5 min at 4 °C in an ice bath for 5 min. After the upper- and lower-phase solutions were separated, both solutions were mixed with 5× volumes of water and centrifuged at 15,000 × g for 30 min at 4 °C to collect the proteins. The PM proteins were in the upper phase, whereas the ICM proteins were in the lower phase.

### Acyl-biotinyl exchange assay

To block free thiols exhaustively, 20 mmol/L methyl methanethiosulfonate (Sigma‒Aldrich) and 1 mmol/L PMSF (Beyotime, China) were used to incubate the cell lysates at 50 °C for 30 min. Proteins were precipitated with acetone and then resuspended in 1 mol/L hydroxylamine (pH 7.4, Sigma‒Aldrich) to promote depalmitoylation. Afterwards, the proteins were incubated with 0.2 mmol/L biotin-HPDP (Top Science) for 1 h at room temperature. Biotinylated proteins were purified with streptavidin (Yeasen) and analysed by immunoblotting.

### ChIP‒qPCR

Chromatin immunoprecipitation (ChIP) followed by quantitative PCR (qPCR) was performed to assess the binding of RelA/p65 (wild-type or K310R mutant) to the CD47 promoter. C4-2B cells were transfected with either wild-type RelA or the K310R-mutant construct and treated as indicated. The cells were crosslinked with 1% formaldehyde for 10 min at room temperature to fix protein‒DNA interactions, followed by quenching with 125 mM glycine for 5 min. Nuclei were isolated, and chromatin was sheared into 200–500 bp fragments by sonication. The lysates were incubated overnight at 4 °C with an anti-RelA/p65 antibody (Cell Signaling Technology; 8242) or control IgG, followed by pull-down with protein A/G magnetic beads. After reversal of cross-linking and purification of the DNA, qPCR was performed using primers targeting the CD47 promoter region containing the NF-κB binding site. The relative enrichment was calculated by normalizing ChIP DNA signals to input controls and expressed as the fold enrichment over IgG. Comparisons between the WT and K310R groups were used to evaluate the impact of K310 acetylation on CD47 promoter binding.

### Click-iT pull-down

Click chemistry and streptavidin pull-down were performed for Click-iT identification of palmitoylation [56]. The cells were incubated with 100 µmol/L Click-iT palmitic acid azide (Thermo Fisher Scientific) for 6 h and then lysed for protein extraction. A Click-iT Protein Reaction Buffer Kit (#C10276; Thermo Fisher Scientific) was used to catalyse the reaction between protein samples and biotin-alkyne. Streptavidin (Yeasen, China) was used to precipitate the biotin–alkyne–azide–palmitic–protein complex. For immunoblotting analysis, the bound proteins were eluted by boiling with SDS‒PAGE sample buffer without DTT for 10 min at 95 °C.

### CRISPR/Cas9 technique

sgRNAs of ZDHHCs were designed with https://www.synthego.com. sgRNAs were then cloned and inserted into the lentiCRISPR v2 vector (Addgene, #52961). The sequences of the sgRNAs were provided in our previous study [57].

### Quantification and statistical analysis

Each experiment was independently repeated at least three times to ensure reproducibility. In the mouse model, the data points represent biological replicates derived from three individual mice; for all in vivo and ex vivo assays, we used three biological replicates (samples from three individual mice per group). For each mouse, samples were analysed in three technical replicates (three separate wells), and the mean of these technical replicates was used as that mouse’s single data point. For qPCR analyses, three technical replicates were performed per sample. For the cell-based experiments, each experimental group included three biological replicates.

Statistical analysis was conducted with GraphPad Prism 7 (GraphPad Software, San Diego, California, USA; RRID: SCR_002798) or R version 3.1.1 (R Foundation for Statistical Computing, Vienna, Austria; RRID: SCR_001905). Comparisons between two groups were performed with either Student’s t test or the Mann–Whitney U test, depending on the distribution and design of the data. For normally distributed data with equal variances, t tests (paired or unpaired; one- or two-tailed, as appropriate) were applied. Normality was assessed by the Shapiro–Wilk test, and homogeneity of variance was tested before applying parametric methods. When the assumption of normality was violated, nonparametric Mann–Whitney U tests were used. For each statistical test, the test statistic, exact p value, degrees of freedom, and sample sizes were reported. Where applicable, effect sizes with 95% confidence intervals and relevant descriptive statistics were included. All analyses assumed random sampling and independence between groups. Tumour volume curves were analysed by two-way ANOVA, with treatment and time as between-subject factors. Both the main effects and their interactions were included in the model. No blocking factors were applied. When appropriate, post hoc tests were conducted to further investigate group differences. The data met the assumptions of normality and homogeneity of variances across groups. The reported statistical outcomes include F statistics, degrees of freedom, exact p values for main effects and interactions, descriptive statistics, and results of post hoc analyses. A p value less than 0.05 was considered statistically significant for all tests.

### Resource availability lead contact

Source Identifier: Please direct any requests for further information or reagents to the lead contact, Yi Sun (yisun666@gzhmu.edu.cn).

Materials Availability: Further information and requests for resources and reagents should be directed to and will be fulfilled by the lead contact, Yi Sun (yisun666@gzhmu.edu.cn).

## Supplementary Information

Supplementary Material 1.

Supplementary Material 2.

Supplementary Material 3.

Supplementary Material 4. Supplemental fig. 2 (A) Cell creep immunofluorescence analysis of several cell lines (multiple stains with DAPI, CXCR2 and CD47); the white bars in the images represent 100 μm. (B) Tumour-associated macrophages (CD11b + F4/80+) identified by flow cytometry from a coculture model of immune cells and tumour cells; the ability of macrophages to engulf tumour cells was assessed by flow cytometry; live, single CD45⁺ cells were gated with FSC/SSC parameters and FSC-A vs. FSC-H plots. Dead cells were excluded with a viability dye. M1 (CD86⁺) and M2 (CD206⁺) macrophages were gated from the CD11b⁺F4/80⁺ population. Fluorescence minus one (FMO) controls were used to define gating thresholds for CD86 and CD206. (C) Differentiation of PBMCs into CD8 + T cells, CD4 + Tregs, M1 macrophages (CD86⁺) and M2 macrophages (CD206⁺) was assessed by flow cytometry, and Ki67 was used as a marker of the proliferation status of CD8 + T cells and Tregs. (D) Cell factors secreted by tumour-associated M1/M2 macrophages were assessed by ELISA, and standard concentrations were prepared by serial 1:2 dilutions of a recombinant protein standard provided by the ELISA kit. The data are presented as the means ±SDs of at least three replicates. NS indicates no significance, * indicates *p* < 0.05, ** indicates *p* < 0.01, and *** indicates *p* < 0.001; *p* < 0.05 was considered statistically significant. Supplemental Fig. 2. (A) Tumour size in different tumours-bearing mice (constructed by different cell lines). (B) Cell proliferation of tumour-infiltrating CD8 + T cells and CD4 + Tregs was determined by Ki67 expression and assessed by flow cytometry; M1 macrophages (CD86⁺) and M2 macrophages (CD206⁺) were assessed by flow cytometry. (C) Cell proliferation of tumour-infiltrating CD8 + T cells and CD4 + Tregs was determined by Ki67 expression and assessed by flow cytometry after PBMC injection; differentiation of M1 macrophages (CD86⁺) and M2 macrophages (CD206⁺) was also assessed by flow cytometry. (D) Cell factors secreted by tumour-associated macrophages when the IL-8/CXCR2 pathway was active were assessed by ELISA. The data are presented as the means ±SDs of at least three replicates. NS indicates no significance, * indicates *p* < 0.05, ** indicates *p* < 0.01, and *** indicates *p* < 0.001; *p* < 0.05 was considered statistically significant. Supplemental Fig. 3. The top 20 differentially expressed genes were analysed, and NEPC and adenocarcinoma patient tissues were subjected to (A) Gene Ontology enrichment (GO) analysis, (B) Kyoto Encyclopedia of Genes and Genomes (KEGG) analysis, (C) Clusters of Orthologous Groups (COG)/Karyotic Orthologous Groups (KOG), (D) lipid-map annotation analysis, (E) regulated subcell classification, and (F) Human Metabolome Database (HMDB) annotation analysis. The data are presented as the means ±SDs of at least three replicates. NS indicates no significance, * indicates *p* < 0.05, ** indicates *p* < 0.01, and *** indicates *p* < 0.001; *p* < 0.05 was considered statistically significant. Supplemental Fig. 4. (A) Schematic diagram of the mechanism by which PDC-E2 regulates acetyl-CoA synthesis. (B) Western bblot of PDC-E2-1 and CPT-1 A in several cell lines, (C) CPT-1 A, PDC-E2-1 and CD47 expression in several cell lines, as determined by PCR. (D) Western blot analysis of membrane-expressed CXCR1 and CD47 in tumour cells following pharmacological inhibition of PDC-E2 (a key acetyl-CoA regulatory enzyme), treatment with a FASN inhibitor (to block palmitate synthesis), or CXCR2 blockade, compared with vehicle-treated controls. (E) Cell creep immunofluorescence analysis demonstrates the expression of CXCR2, PDC-E2, CPT-1 A, and CD47 in several cell lines, including LNCaP/MDVR, LNCaP/CXCR2 + IL-8, C4-2B/MDVR, C4-2B/CXCR2 + IL-8, NCI-H660, and PC3, and the white bars in the images represent 100 μm. (F) Immunofluorescence staining analysis shows the expression of CPT-1 A, CD47, and CXCR2 in tumour tissues from tumour-bearing mice implanted with PC3, C4-2B/MDVR, and C4-2B/CXCR2 + IL-8 cells. The data are presented as the means ±SDs of at least three replicates. NS indicates no significance, * indicates *p* < 0.05, ** indicates *p* < 0.01, and *** indicates *p* < 0.001; *p* < 0.05 was considered statistically significant. Supplemental Fig. 5. (A) The expression levels of tumour CXCR1 and CD47, as well as the proliferation of CD8⁺ T cells, Tregs, and NK cells and the infiltration of M1 macrophages (CD86⁺) and M2 macrophages (CD206⁺), were analysed by flow cytometry following PDC-E2 inhibition, FAS inhibition, or CXCR2 blockade. (B) Macrophage migration was assessed with a Transwell assay following CXCR2 blockade in tumour cells within the coculture model. The black bars in the images represent 200 μm. (C) Biological process (BP) analysis, KEGG analysis, and MF analysis of NEPC and adenocarcinoma patient tissues. Tumour tissues from the model mice were subjected to the following analyses: (D) BP analysis, KEGG analysis, and MF analysis of C4-2B/MDVR tumour tissue and C4-2B tumour tissue. (E) BP analysis, KEGG analysis, and molecular function (MF) analysis were also performed on PC 3 tumour tissue and PC 3/CXCR2 KD tumour tissue. (F) Western blot of RelA K310 acetylation and histone 3 acetylation in several cell lines. (G-H) Acetylated p65 (NF-κB p65AC) in the nucleus; histone H3 is shown as a loading control of NF-κB p65AC. (I-J) To block acetylation at the K310 site of RelA/p65 in C4-2B cells treated with acetyl-CoA, we generated a lysine-to-arginine point mutation (K310R) in the RelA/p65 expression plasmid by site-directed mutagenesis and then assessed whether RelA binds to the CD47 promoter with ChIP‒PCR. (K) GSEA was performed on the differentially expressed genes, with a focus on the NF-κB pathways (data collected from public publication databases). The data are presented as the means ±SDs of at least three replicates. NS indicates no significance, * indicates *p* < 0.05, ** indicates *p* < 0.01, and *** indicates *p* < 0.001; *p* < 0.05 was considered statistically significant. Supplemental Fig. 6. (A) CD47 luciferase activity in cell lines treated with palmitic acid, FAO enhancer L-carnitine, citrate, or acetyl-CoA at the indicated concentrations for 48 h and in cell lines after activation of the IL-8/CXCR2 pathway. (B) NF-κB luciferase activity in cell lines treated with palmitic acid, FAO enhancer L-carnitine, citrate, or acetyl-CoA at the indicated concentrations for 48 h and in cell lines after activation of the IL-8/CXCR2 pathway. (C) Acetylated p65 (NF-κB p65AC) in the nucleus; histone H3 is shown as a loading control for NF-κB p65AC. (D) Macrophage migration test by the Transwell method in several cell lines. The black bars in the images represent 200 μm; PC-3 and DU145 cells were treated with AC-CoA and palmitic acid for 48 h. (E) The subcellular fractions were extracted and subjected to Western blot analysis. (F) The protein level was quantified with ImageJ software. The data are presented as the means ±SDs of at least three replicates. NS indicates no significance, * indicates *p* < 0.05, ** indicates *p* < 0.01, and *** indicates *p* < 0.001; *p* < 0.05 was considered statistically significant. Supplemental Fig. 7. (A) The expression levels of M2 marker genes were used to estimate M2 cell levels; all samples were then used as a threshold to classify samples into two groups: high M2 and low M2. The comparison of the log2-transformed expression of CD47 in these two groups is visualized in a boxplot. (B-C) Quantification of MS data from samples collected from mice bearing tumours established from different cell lines. Data are presented as the means ±SDs of at least three replicates. NS indicates no significance, * indicates *p* < 0.05, ** indicates *p* < 0.01, and *** indicates *p* < 0.001; *p* < 0.05 is considered statistically significant. Supplemental Fig. 8. (A-B) IHC staining for CXCR2, CXCL15, FAS, ELOVL5 and CD47 expression in RM-1 and RM-1/CXCR2 tumour-bearing C57BL/6 mice with/without CXCL15 mutation; the black bars in the IHC images represent 200 μm. (C-D) IHC staining for 15d-PGJ2 and maresin-1 expression in RM-1 and RM-1/CXCR2 tumour-bearing C57BL/6 mice with/without CXCL15 mutation; the black bars in the IHC images represent 200 μm. (E) Isolation of splenic macrophages from NSG mice cocultured with different tumour cells or supplemented with various lipids, followed by nuclear protein extraction and Western blot analysis to assess activation of the PPAR-γ pathway. The data are presented as the means ±SDs of at least three replicates. NS indicates no significance, * indicates *p* < 0.05, ** indicates *p* < 0.01, and *** indicates *p* < 0.001; *p* < 0.05 was considered statistically significant. Supplemental Fig. 9. (A) Cell proliferation of tumour-infiltrating CD8 + T cells and CD4 + Tregs determined by Ki67 expression and assessed by flow cytometry; the differentiation of M1 macrophages (CD86⁺) and M2 macrophages (CD206⁺) after treatment with navarixin was assessed by flow cytometry. (B) Macrophage migration test by the Transwell method in several cell lines; the black bars in the images represent 200 μm. (C) Cell factors secreted by tumour-associated macrophages in CXCL15-mutant mice were assessed by ELISA. (D) Cell proliferation of tumour-infiltrating CD8 + T cells and CD4 + Tregs was determined by Ki67 expression and assessed by flow cytometry; the differentiation of M1 macrophages (CD86⁺) and M2 macrophages (CD206⁺) in CXCL15-mutant mice was also assessed by flow cytometry. The data are presented as the means ±SDs of at least three replicates. NS indicates no significance, * indicates *p* < 0.05, ** indicates *p* < 0.01, and *** indicates *p* < 0.001; *p* < 0.05 was considered statistically significant. 

Supplementary Material 5.

## Data Availability

No datasets were generated or analysed during the current study.
