# Supplementary figures and images for: Targeting CXCR2 in prostate cancer cells can block CD47-SIRPα interaction and reverse M2 macrophage polarization in the TME
Source: Mol Cancer. 2025 Oct 30;24:273. doi: 10.1186/s12943-025-02436-1 (PMC12574227; doi:10.1186/s12943-025-02436-1)

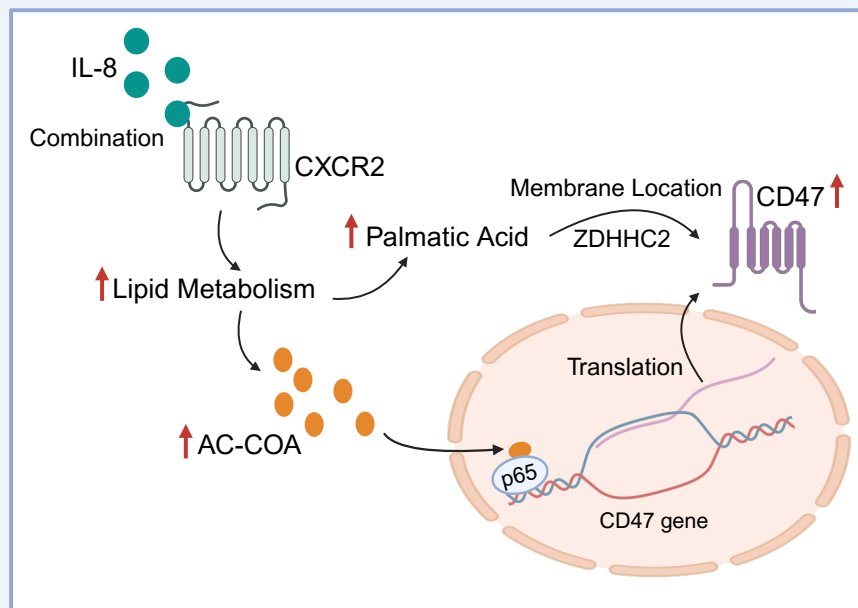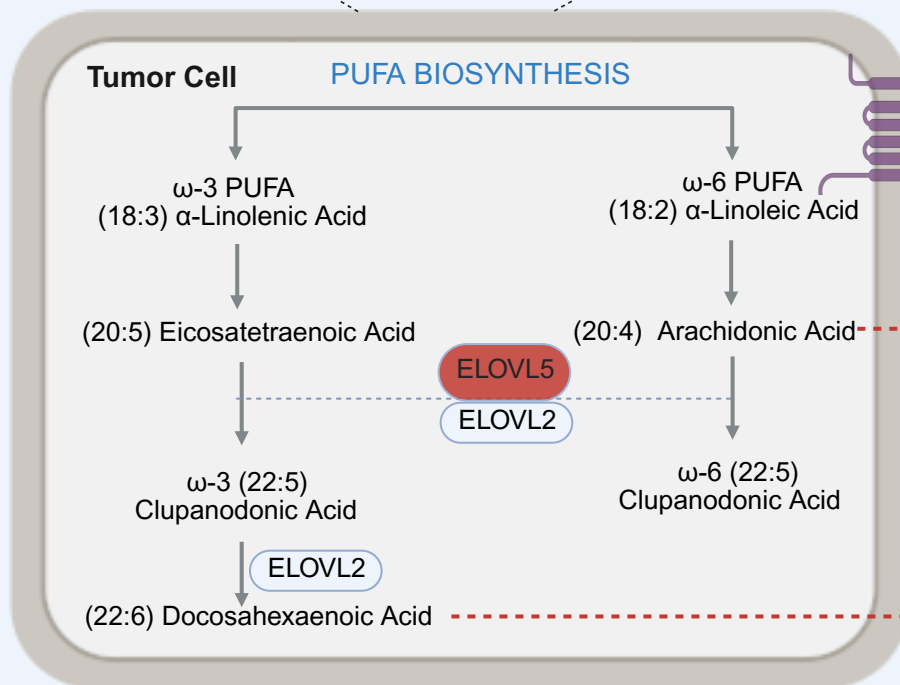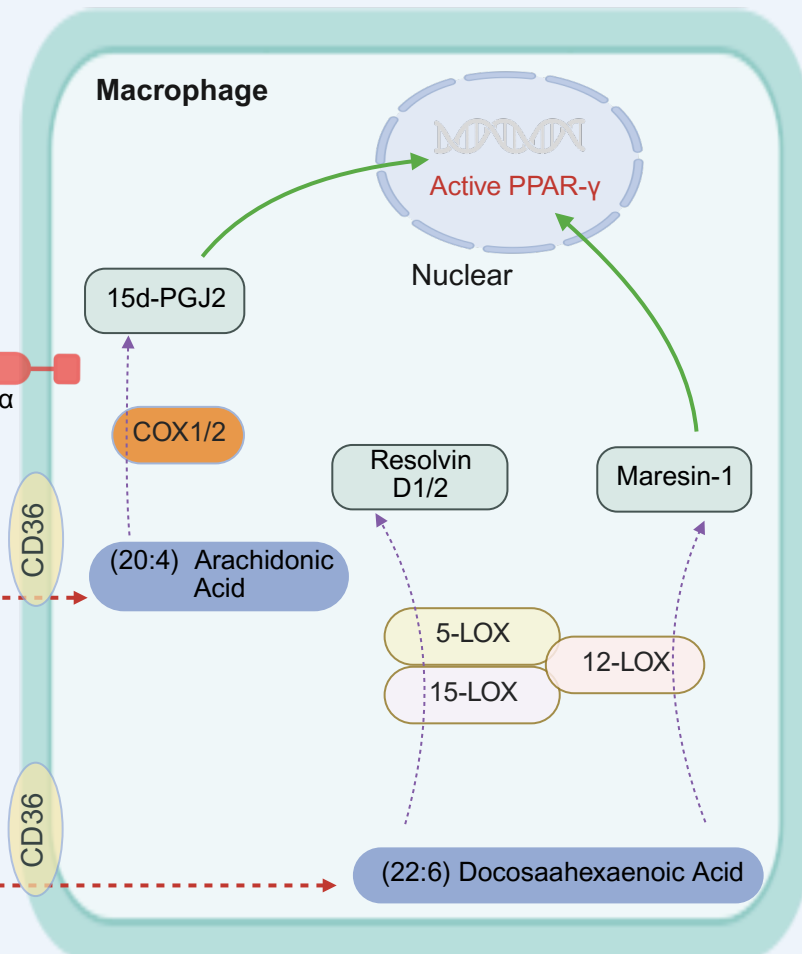

Supplement: Supplementary file 1 — Supplementary Material 1. [file 12943_2025_2436_MOESM1_ESM.pdf]

Figure 2h

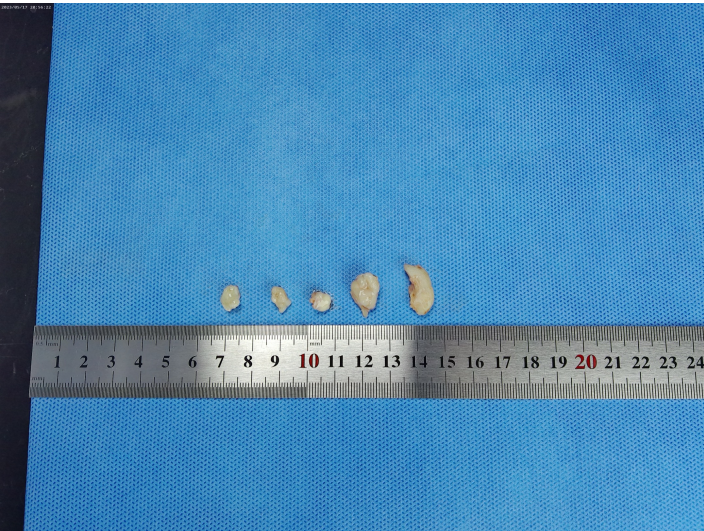

S-Figure 2b

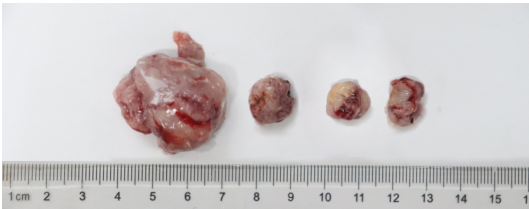

Figure 7b

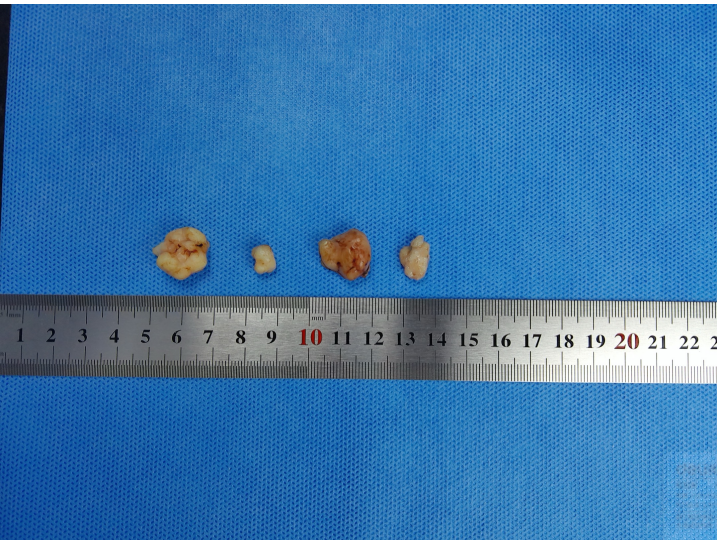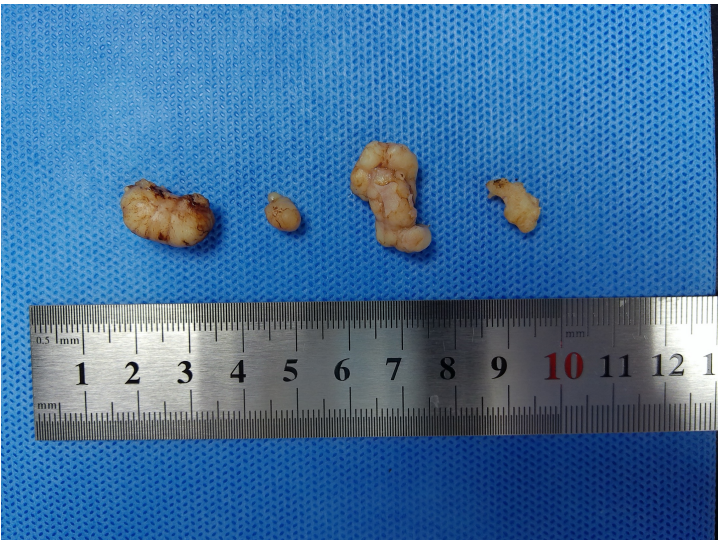

Supplement: Supplementary file 3 — Supplementary Material 3. [file 12943_2025_2436_MOESM3_ESM.pdf]

Figure 1G

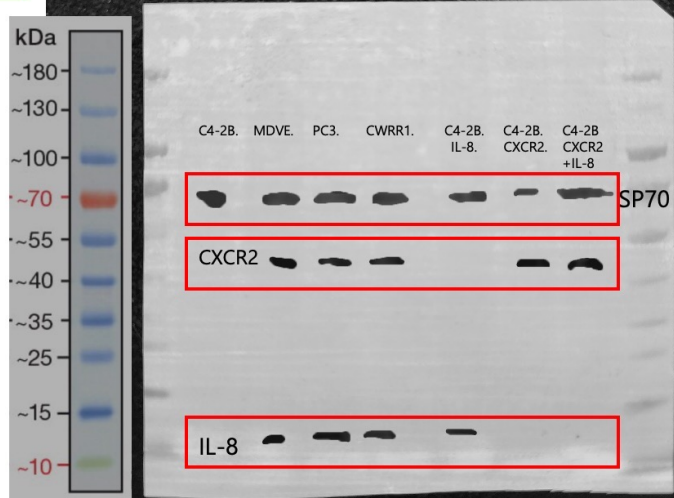

Figure 1H

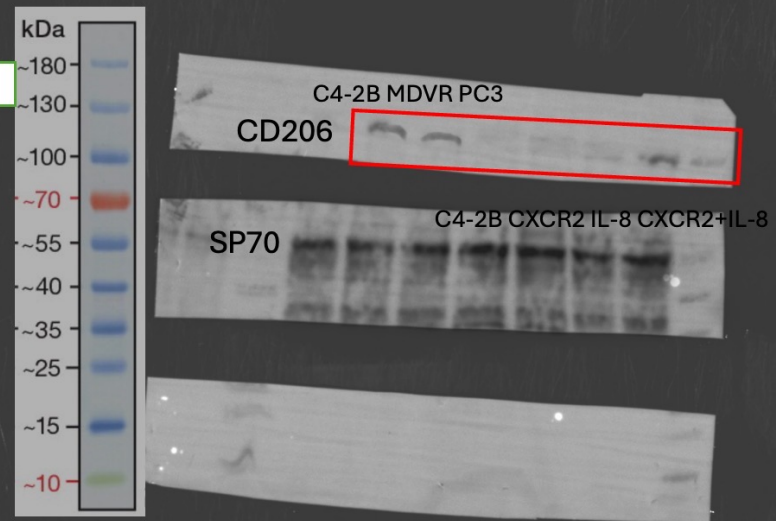

Figure 3I

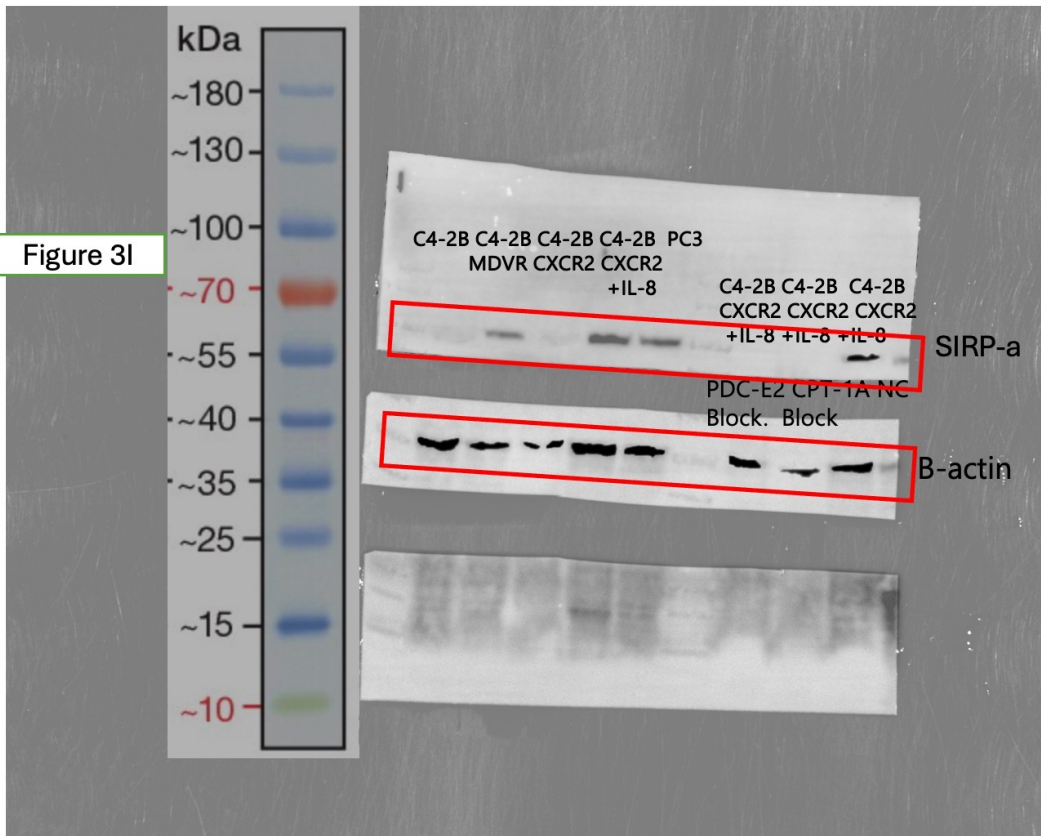

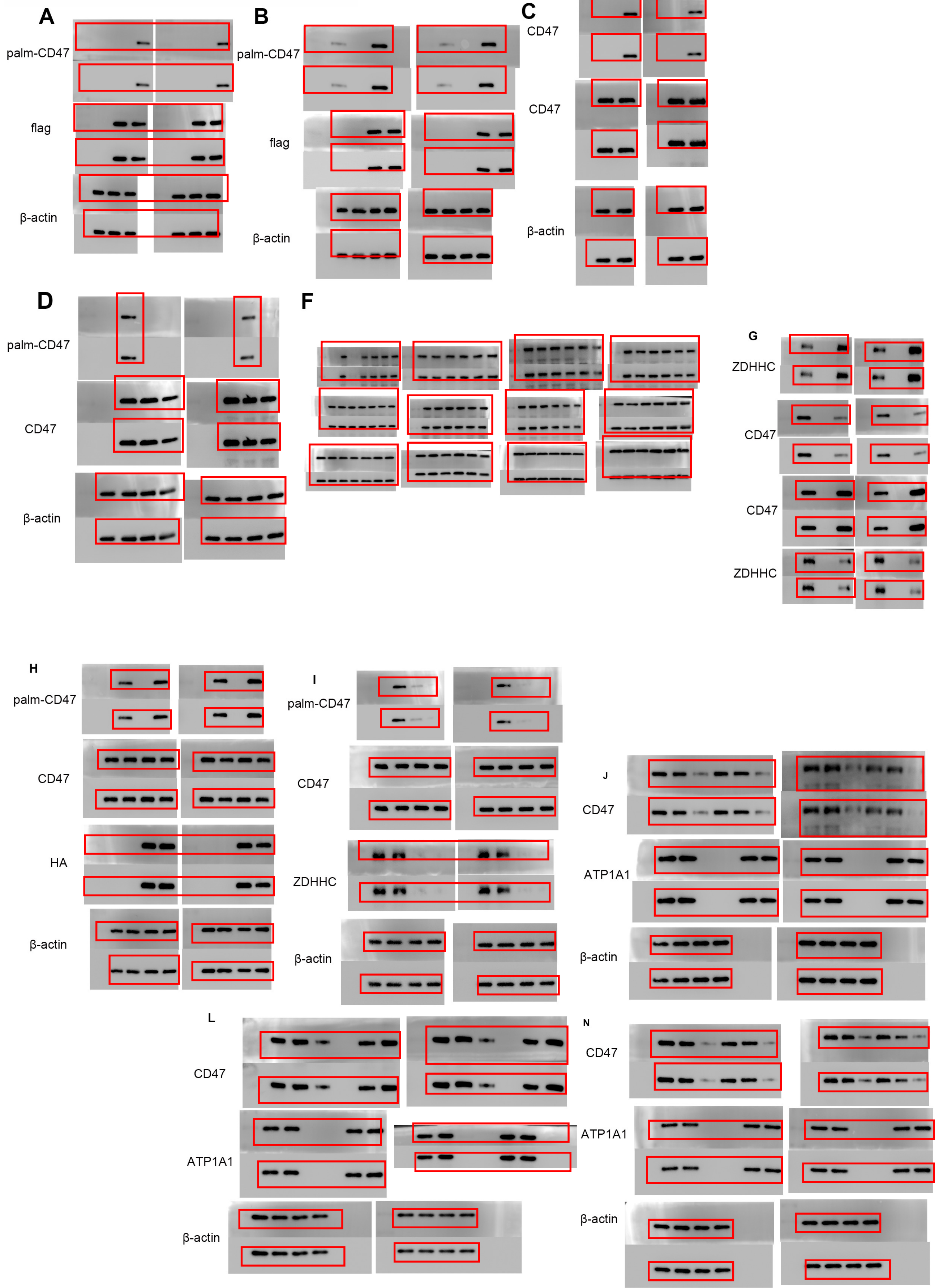

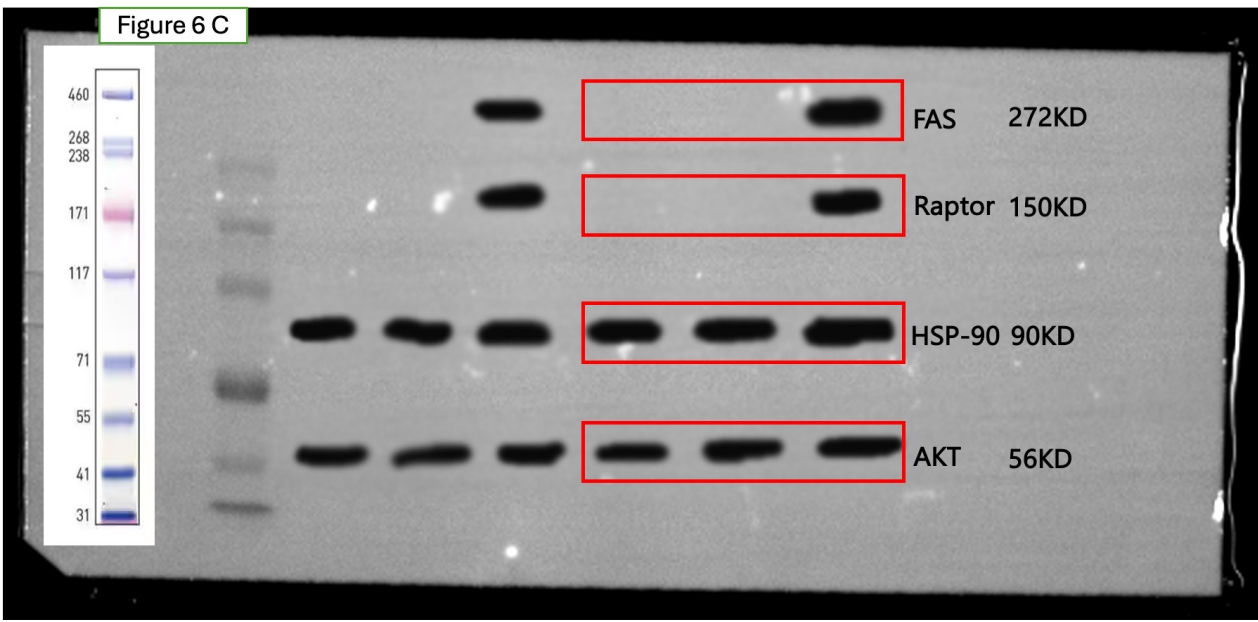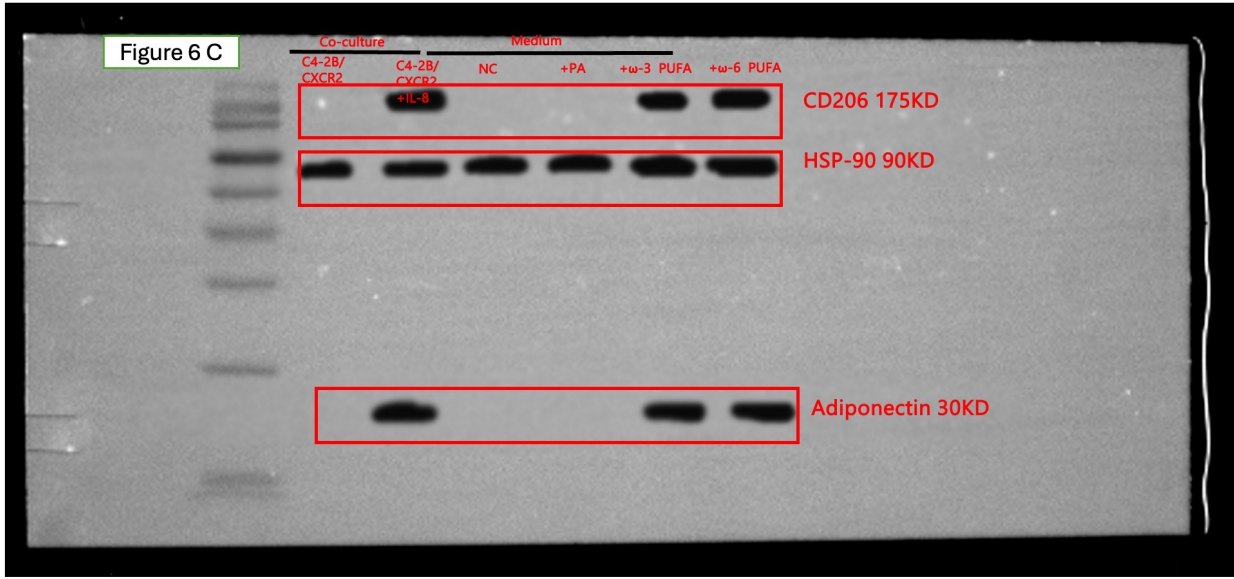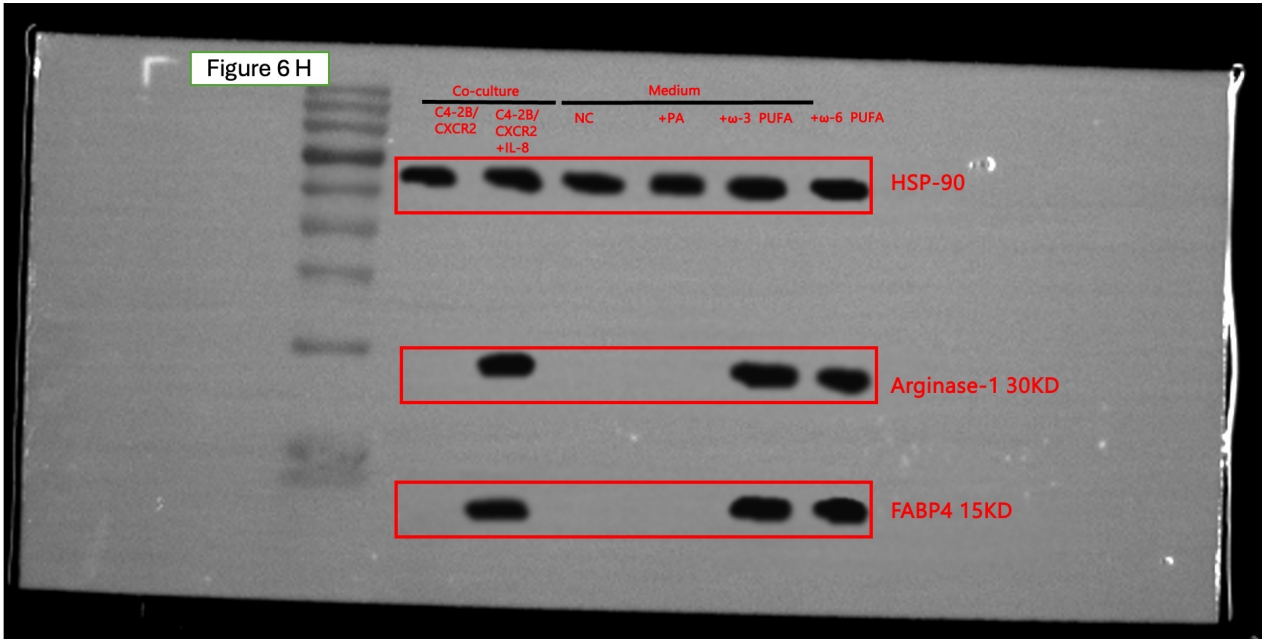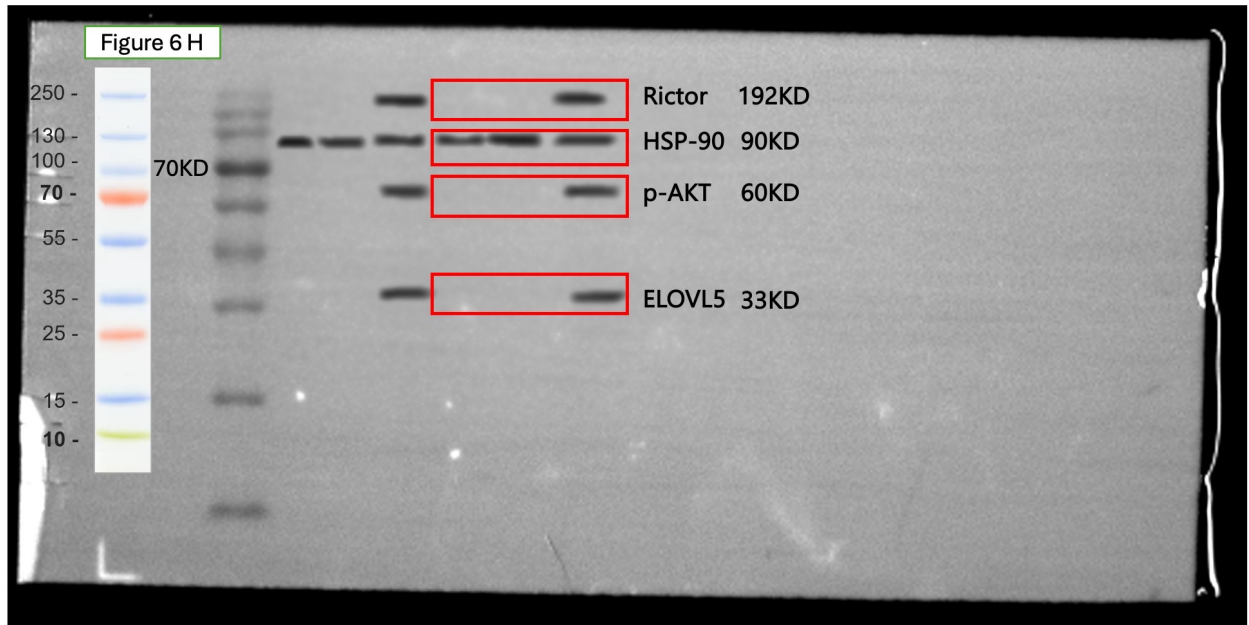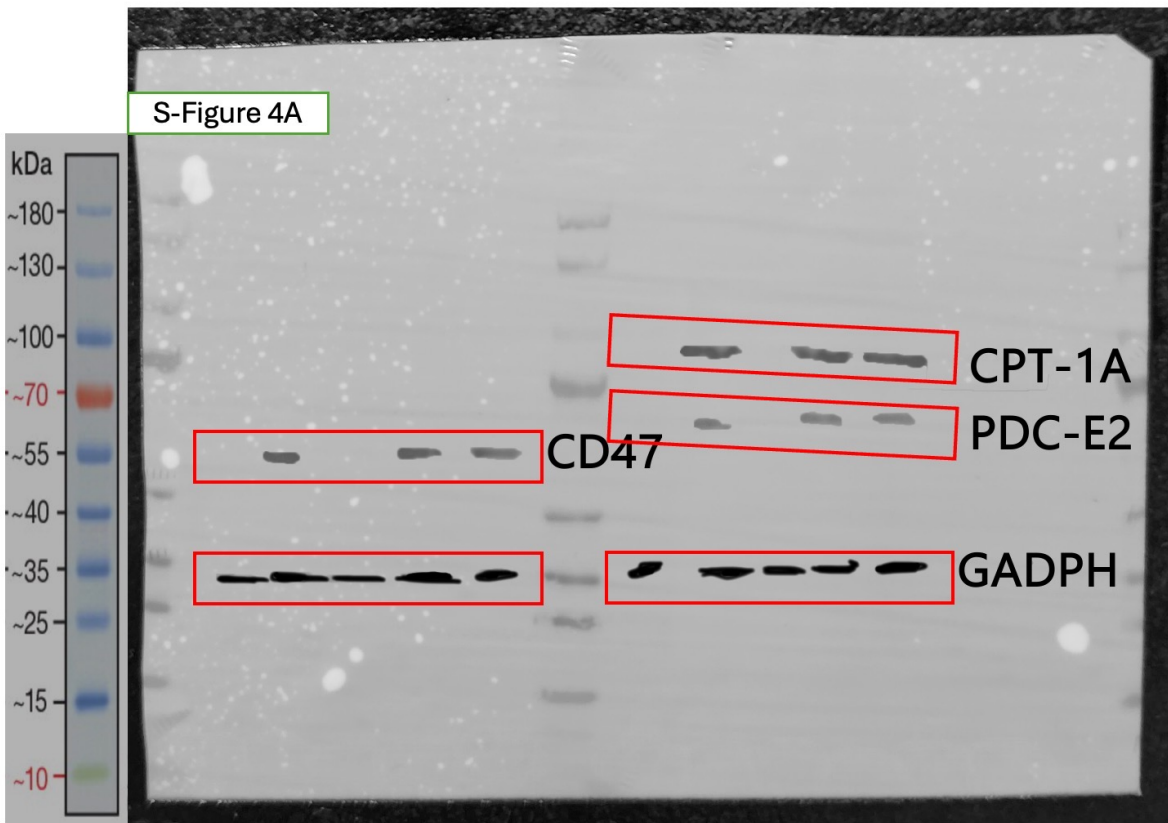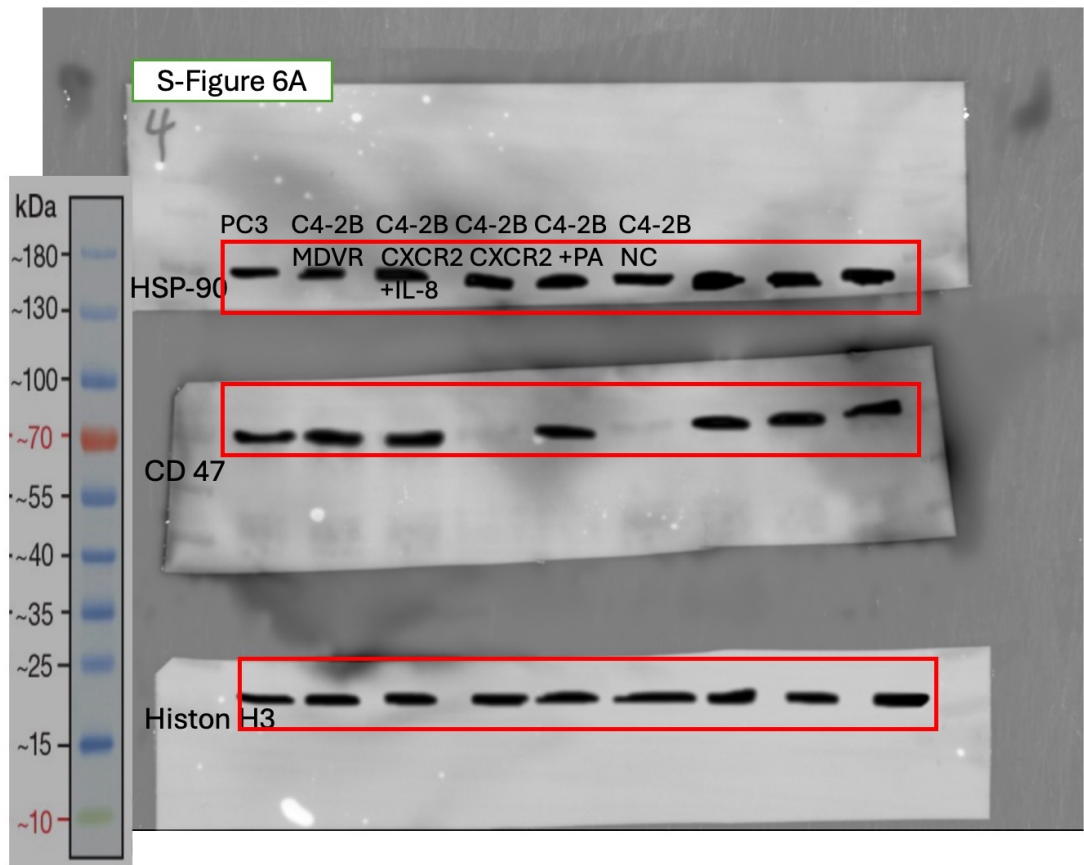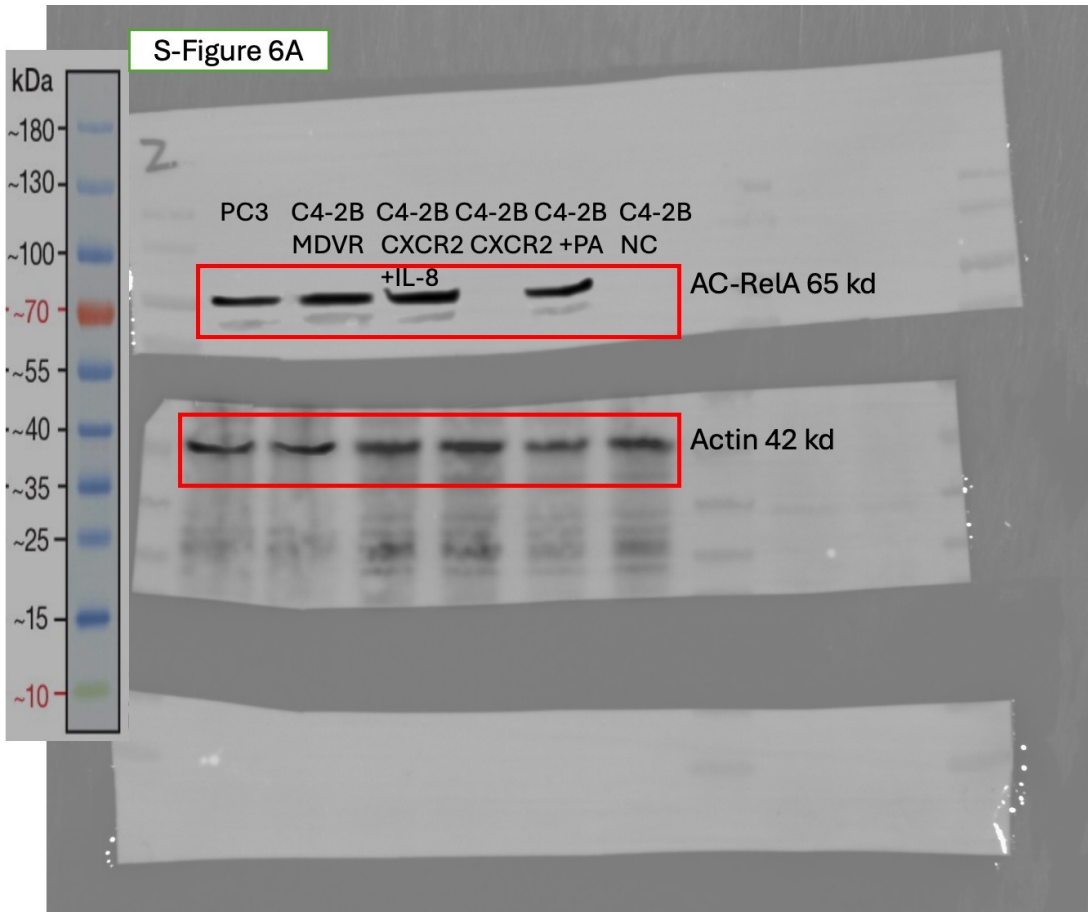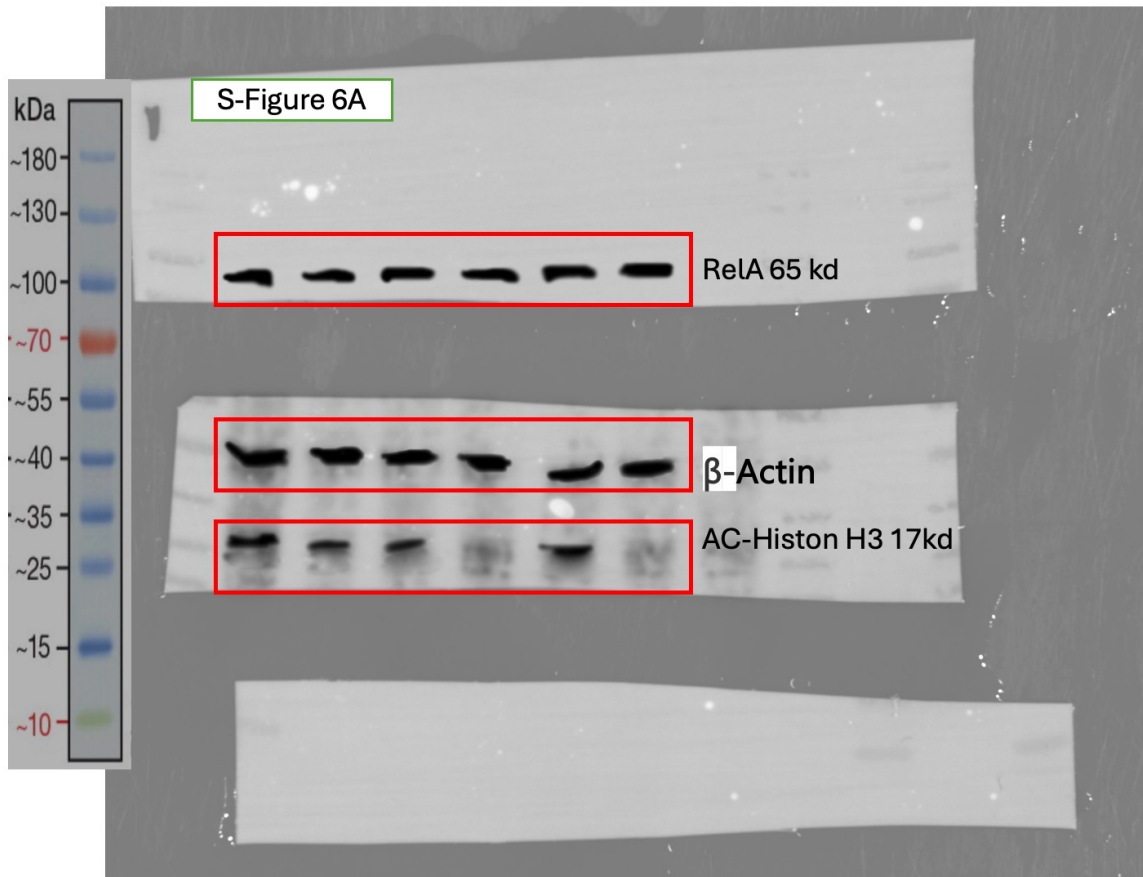

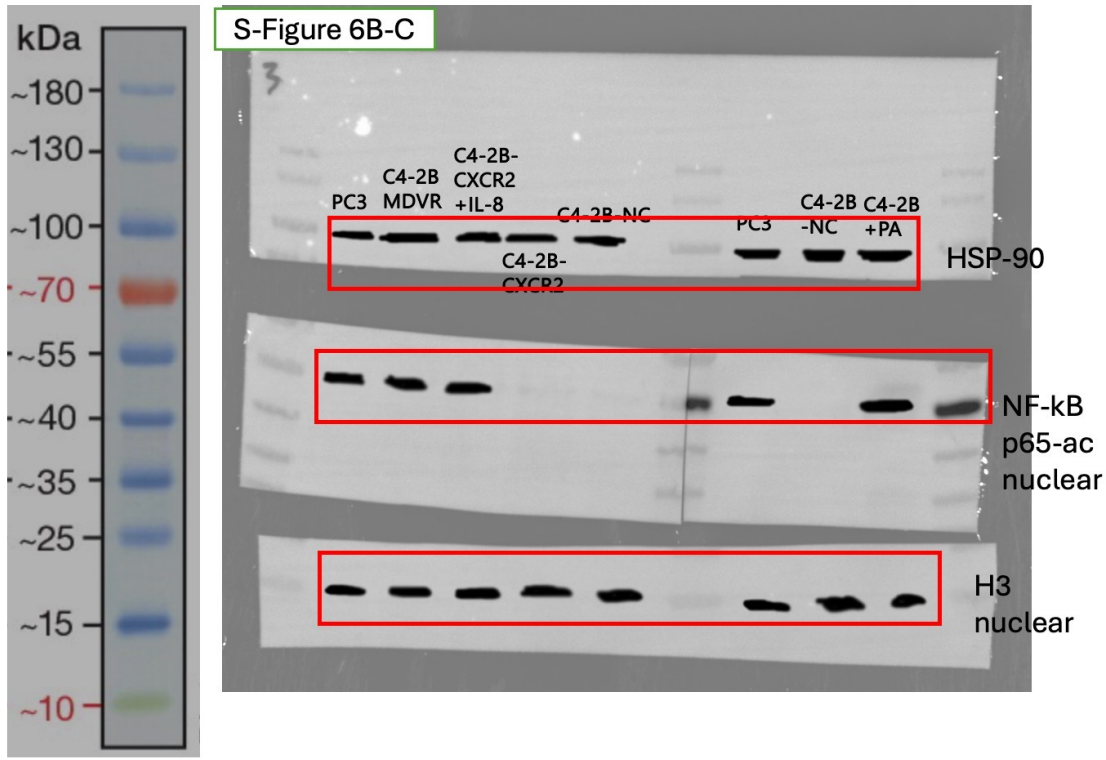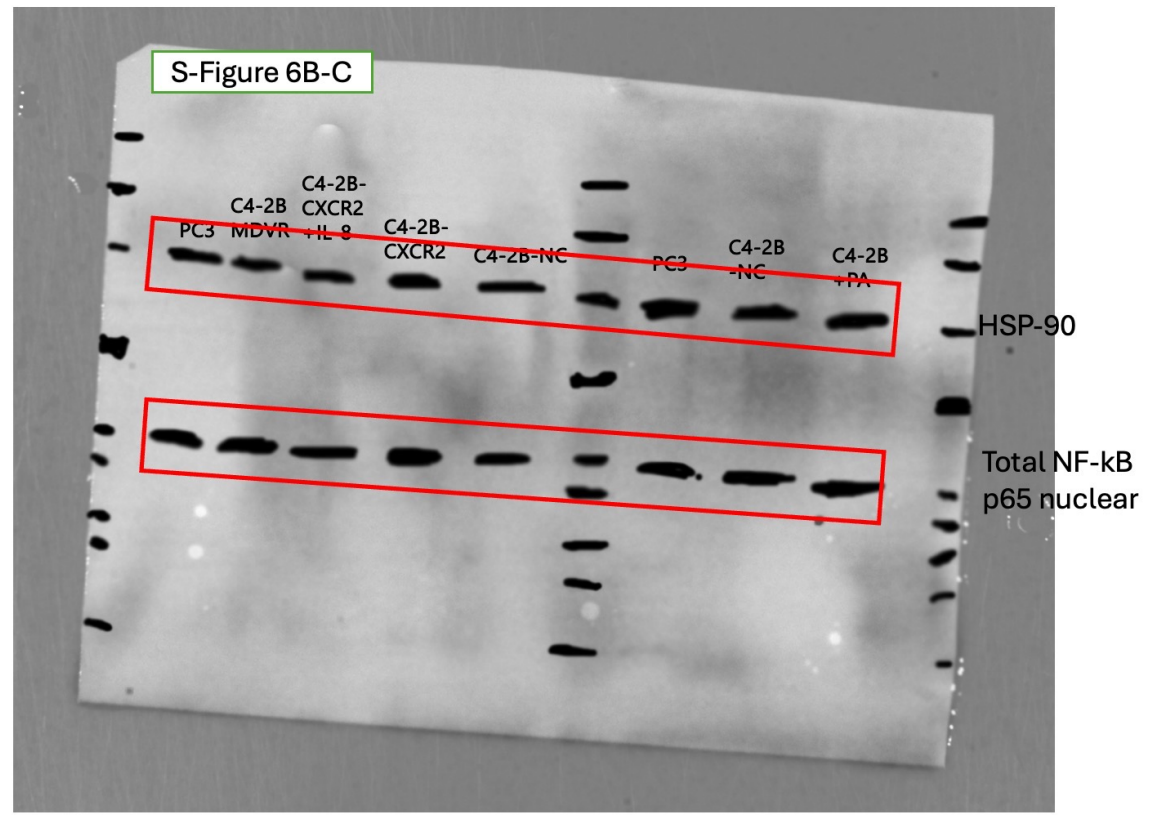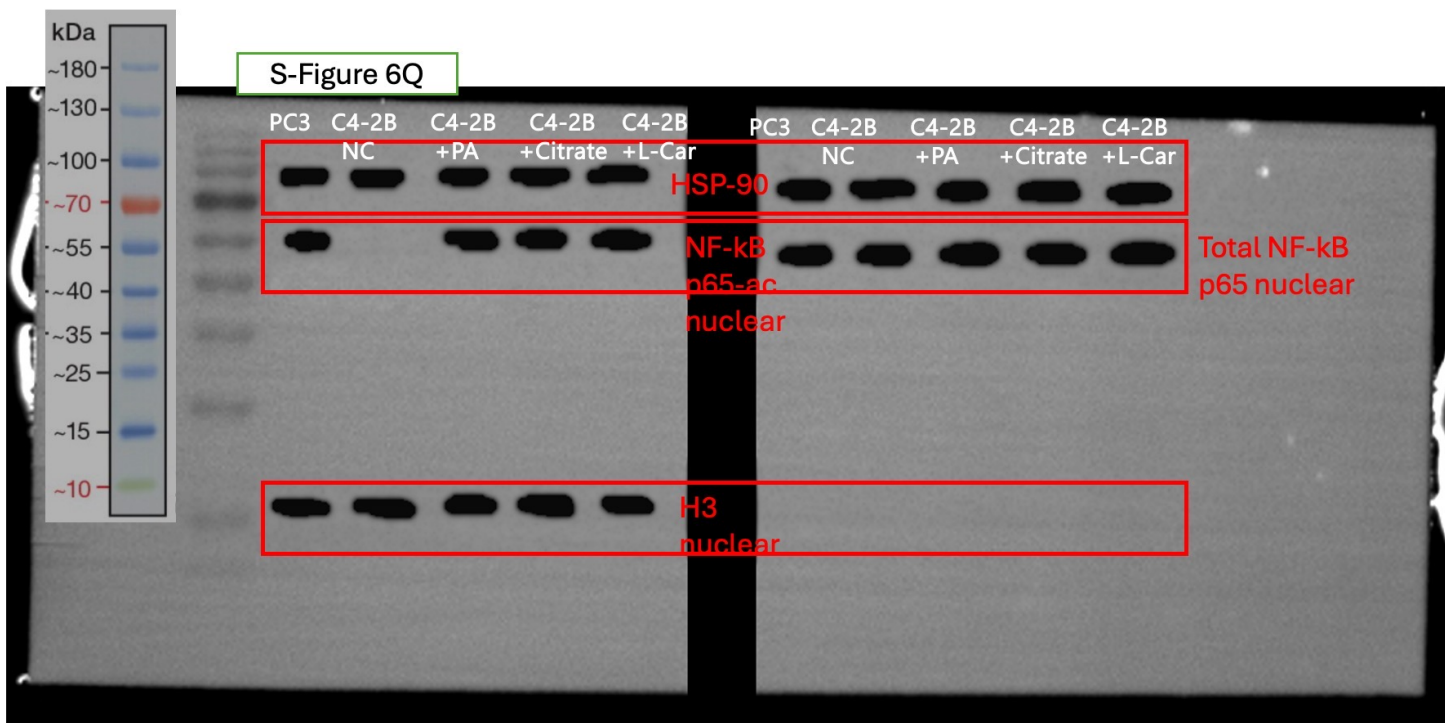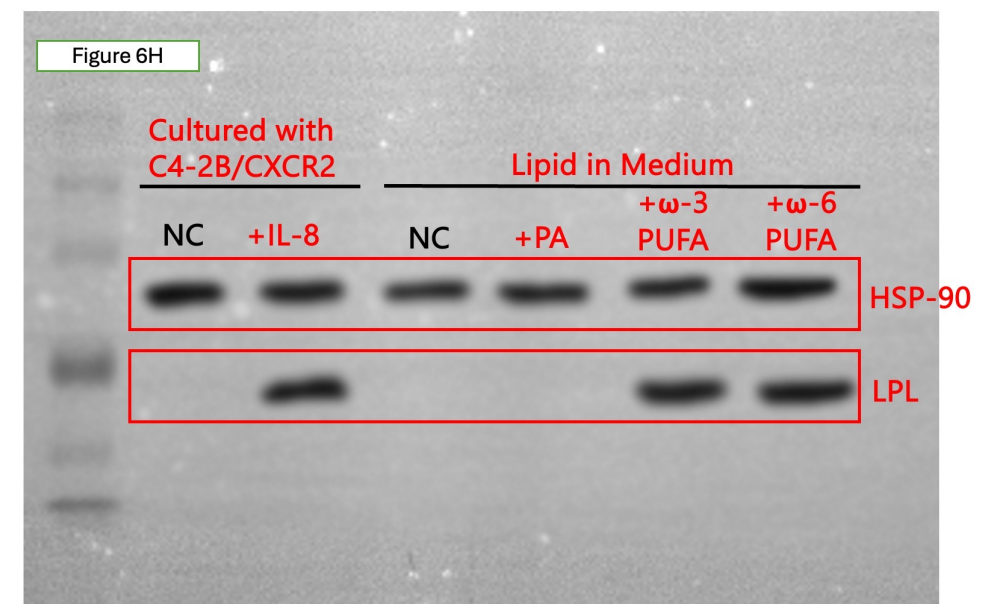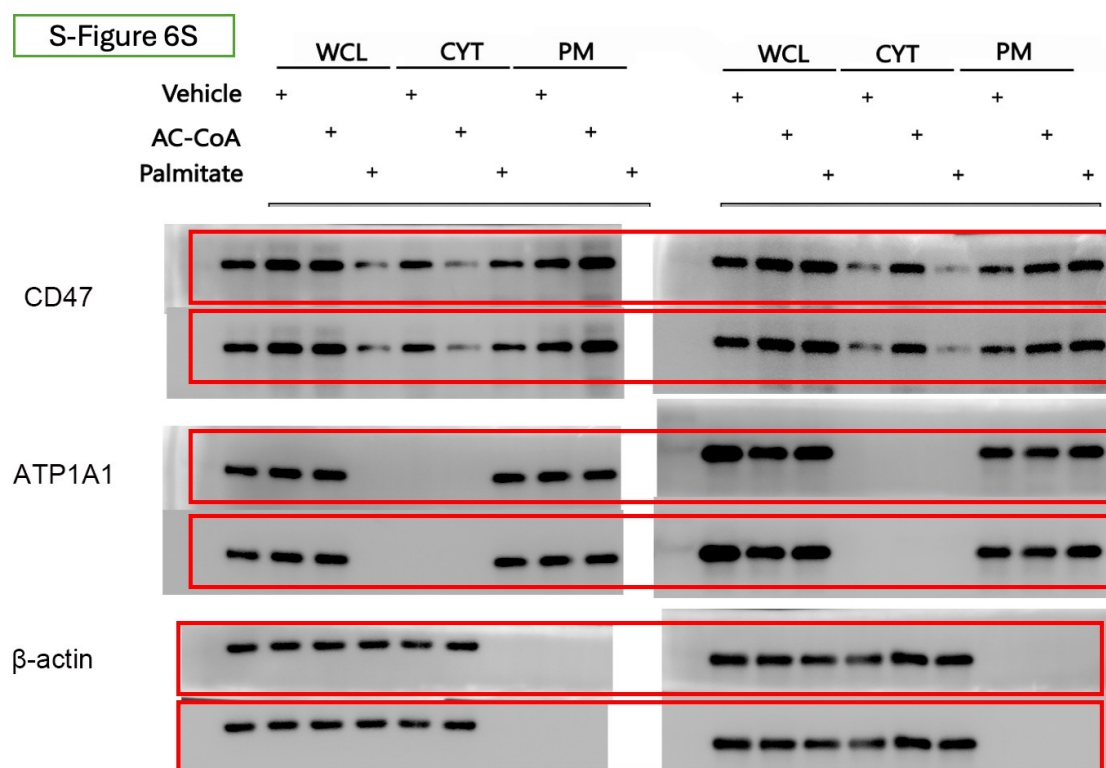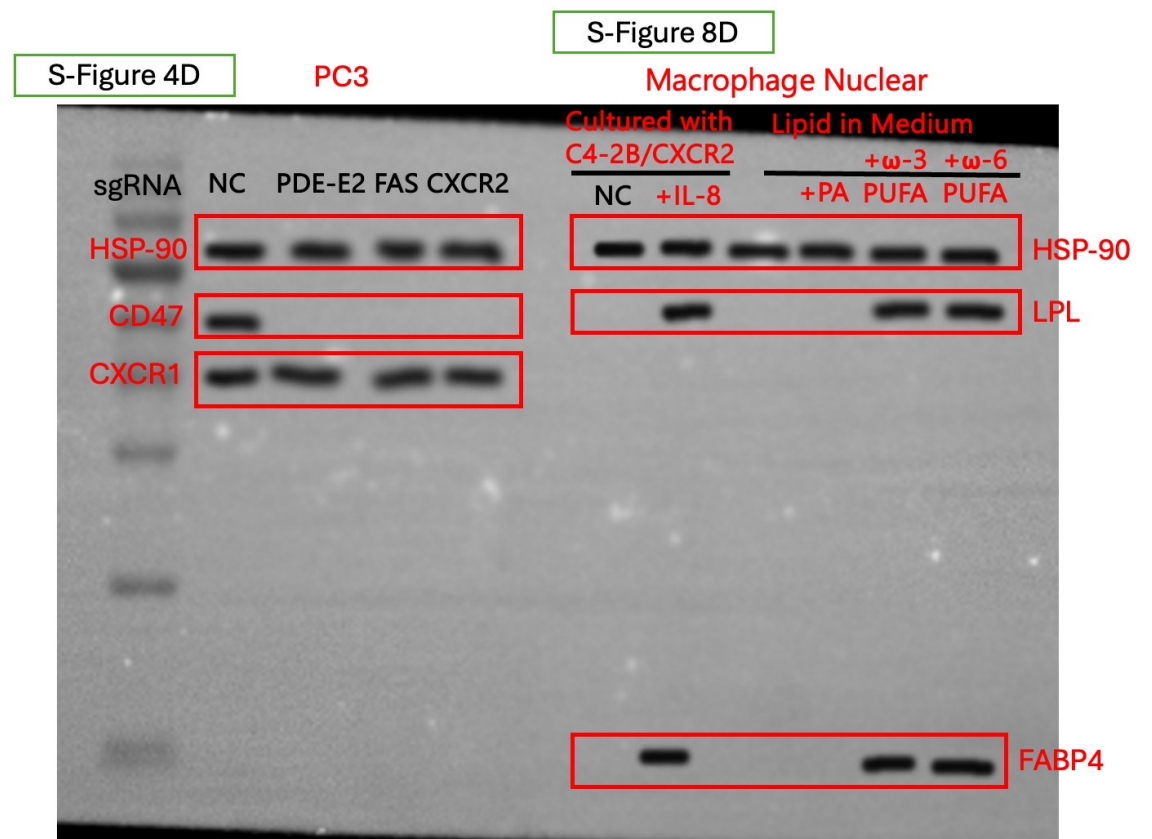

Supplement: Supplementary file 5 — Supplementary Material 5. [file 12943_2025_2436_MOESM5_ESM.pdf]
