# Supplementary material for: Targeting CXCR2 in prostate cancer cells can block CD47-SIRPα interaction and reverse M2 macrophage polarization in the TME
Source: Mol Cancer. 2025 Oct 30;24:273. doi: 10.1186/s12943-025-02436-1 (PMC12574227; doi:10.1186/s12943-025-02436-1)
Supplement: Supplementary file 2 — Supplementary Material 2. [file 12943_2025_2436_MOESM2_ESM.doc]

**Table 1** Reagent, Antibody and Sequences list

| **Reagent** | **Manufacturers** | **Catalog Number** |
| --- | --- | --- |
| **IL-8 (human)** | huabio | HA210642 |
| **THBS1 / Thrombospondin-1 (human)** | LSBio | LS-G131343 |
| **THBS1 / Thrombospondin-1 (mice)** | LSBio | LS-G12478 |
| **PCR Kits** | huabio | HB122332 |
| **ELISA Kits CXCL10** | elascience | E-EL-H0050 |
| **ELISA Kits CXCL11** | elascience | E-EL-H0051 |
| **ELISA Kits CCL 1** | elascience | E-EL-H6226 |
| **ELISA Kits CCL 17** | elascience | E-EL-H6228 |
| **ELISA Kits CCL 22** | elascience | E-EL-H0106 |
| **ELISA Kits 4-HNE** | elascience | E-EL-H0302 |
| **IL-8 (human)** | huabio | HA210642 |
|  |  |  |
| **Antibody** | **Manufacturers** | **Catalog Number** |
| **CXCR2 (WB & IHC)** | huabio | ER1906-87 |
| **CD 47 (WB & IHC& FCM)** | ThermoFisher | MA5-11895 |
| **IL-8 (WB & IHC)** | huabio | ER1901-61 |
| **Glut-1 (WB & IHC)** | ThermoFisher | 6513-MSM4-P0 |
| **CPT-1A (WB & IHC)** | ThermoFisher | MA5-46944 |
| **SIRP-α (WB)** | ThermoFisher | MA5-44115 |
| **CD 68 (WB & IHC)** | Santa Cruz | CAS 107430-66-0 |
| **CD 86 (WB & IHC)** | Santa Cruz | CAS 1150586-64-3 |
| **CD 206 (WB & IHC)** | Santa Cruz | CAS 125333-60-1 |
| **AC-RELA (WB)** | Santa Cruz | sc-71677 |
| **RELA (WB)** | Santa Cruz | sc-71677 |
| **HISTON-H3 (WB)** | Santa Cruz | sc-24516 |
| **AC-HISTON-H3 (WB)** | Santa Cruz | sc-518011 |
| **NF-kB p65 AC Nuclear (WB)** | Santa Cruz | sc-7151 |
| **H3-Nuclear (WB)** | Santa Cruz | sc-57946 |
| **Total NF-kB p65 Nuclear (WB)** | Santa Cruz | sc-109 |
| **CD11B+(WB)** | ThermoFisher | MA-44076 |
| **F4/80+ (FCM)** | ThermoFisher | MF48005 |
| **CD11B+(FCM)** | ThermoFisher | A18613 |
| **CD68(FCM)** | ThermoFisher | MA5-13324 |
| **CD86 (FCM)** | ThermoFisher | MHCD8601 |
| **CD206(FCM)** | ThermoFisher | MA5-16872 |
| **CD56(FCM)** | BD Biosciences | 555516 |
| **Ki67(FCM)** | huabio | HA721115 |
|  |  |  |
| **Gene** | **sh/siRNA** | **Targeted Sequence 5'-3'** |
| **CD47** | shRNA | GGAACAGCTTGTTGTACTTAT |
| **CXCR2** | shRNA | GGA​GGA​GUA​UCU​GGA​GAA​A |
| **IL-8** | shRNA | GGA​GAU​GGA​GAG​UGC​UGA​A |
| **ELOVL5** | siRNA | GGA​GAU​GGA​GAG​UGC​UGA​A |
| **RelA** | K310 site mutation | **F: 5′-GAG GGA AGA AGT TCA AG**AGGCC AAA AGA GAC CCA CAA G-3 |
|  |  | R: 5′-CTT GTG GGT CTC TTT TTCCTC TTG TTT CTT CCC TC-3 |
