# Supplementary material for: Targeting CXCR2 in prostate cancer cells can block CD47-SIRPα interaction and reverse M2 macrophage polarization in the TME
Source: Mol Cancer. 2025 Oct 30;24:273. doi: 10.1186/s12943-025-02436-1 (PMC12574227; doi:10.1186/s12943-025-02436-1)
Supplement: Supplementary file 4 — Supplementary Material 4. Supplemental fig. 2 (A) Cell creep immunofluorescence analysis of several cell lines (multiple stains with DAPI, CXCR2 and CD47); the white bars in the images represent 100 μm. (B) Tumour-associated macrophages (CD11b + F4/80+) identified by flow cytometry from a coculture model of immune cells and tumour cells; the ability of macrophages to engulf tumour cells was assessed by flow cytometry; live, single CD45⁺ cells were gated with FSC/SSC parameters and FSC-A vs. FSC-H plots. Dead cells were excluded with a viability dye. M1 (CD86⁺) and M2 (CD206⁺) macrophages were gated from the CD11b⁺F4/80⁺ population. Fluorescence minus one (FMO) controls were used to define gating thresholds for CD86 and CD206. (C) Differentiation of PBMCs into CD8 + T cells, CD4 + Tregs, M1 macrophages (CD86⁺) and M2 macrophages (CD206⁺) was assessed by flow cytometry, and Ki67 was used as a marker of the proliferation status of CD8 + T cells and Tregs. (D) Cell factors secreted by tumour-associated M1/M2 macrophages were assessed by ELISA, and standard concentrations were prepared by serial 1:2 dilutions of a recombinant protein standard provided by the ELISA kit. The data are presented as the means ±SDs of at least three replicates. NS indicates no significance, * indicates p < 0.05, ** indicates p < 0.01, and *** indicates p < 0.001; p < 0.05 was considered statistically significant. Supplemental Fig. 2. (A) Tumour size in different tumours-bearing mice (constructed by different cell lines). (B) Cell proliferation of tumour-infiltrating CD8 + T cells and CD4 + Tregs was determined by Ki67 expression and assessed by flow cytometry; M1 macrophages (CD86⁺) and M2 macrophages (CD206⁺) were assessed by flow cytometry. (C) Cell proliferation of tumour-infiltrating CD8 + T cells and CD4 + Tregs was determined by Ki67 expression and assessed by flow cytometry after PBMC injection; differentiation of M1 macrophages (CD86⁺) and M2 macrophages (CD206⁺) was also assessed by f [file 12943_2025_2436_MOESM4_ESM.pdf]

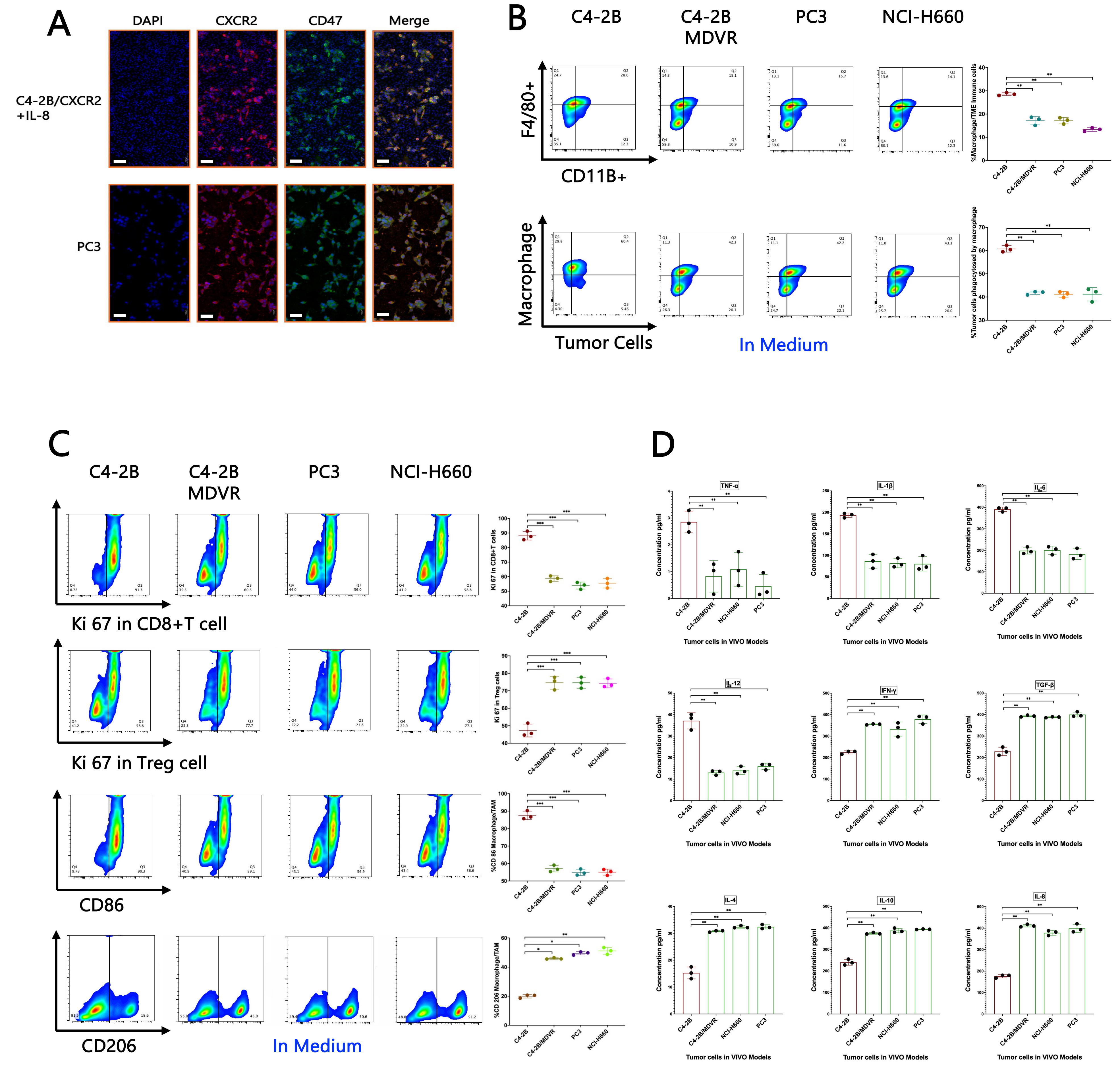

Supplemental Figure 1

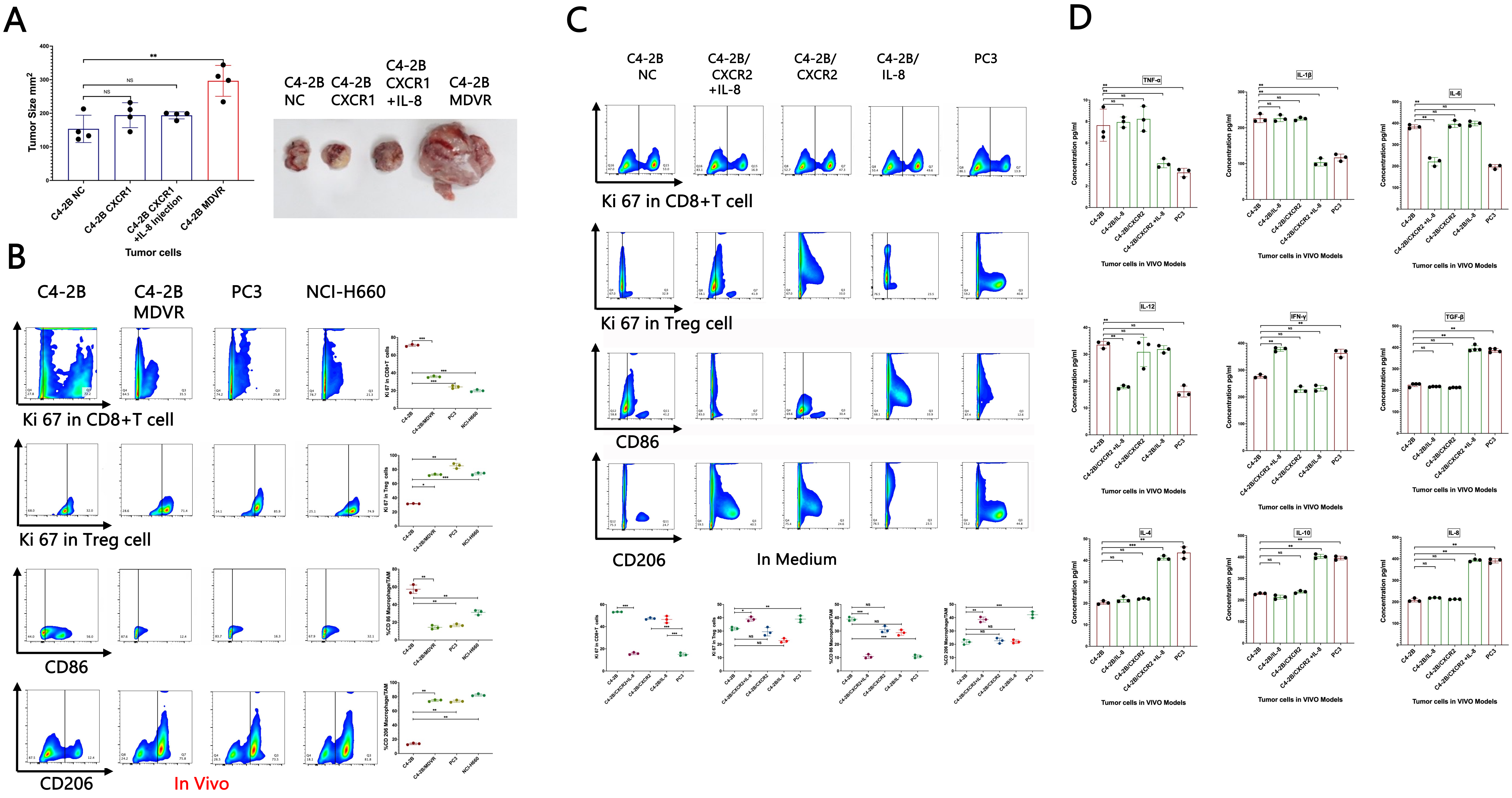

Supplemental Figure 2

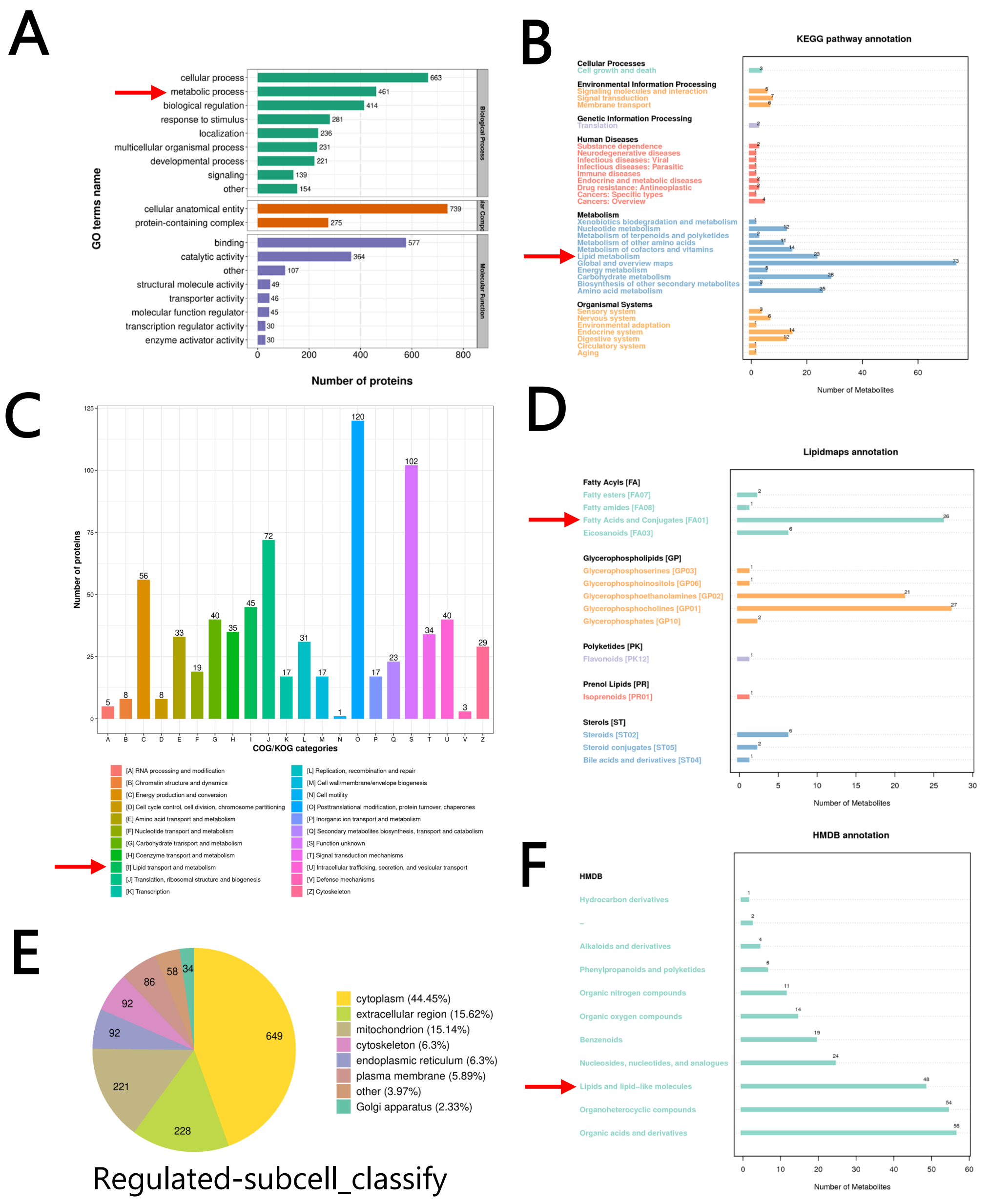

NEPC Compared with Adenocarcinoma

Supplemental Figure 3

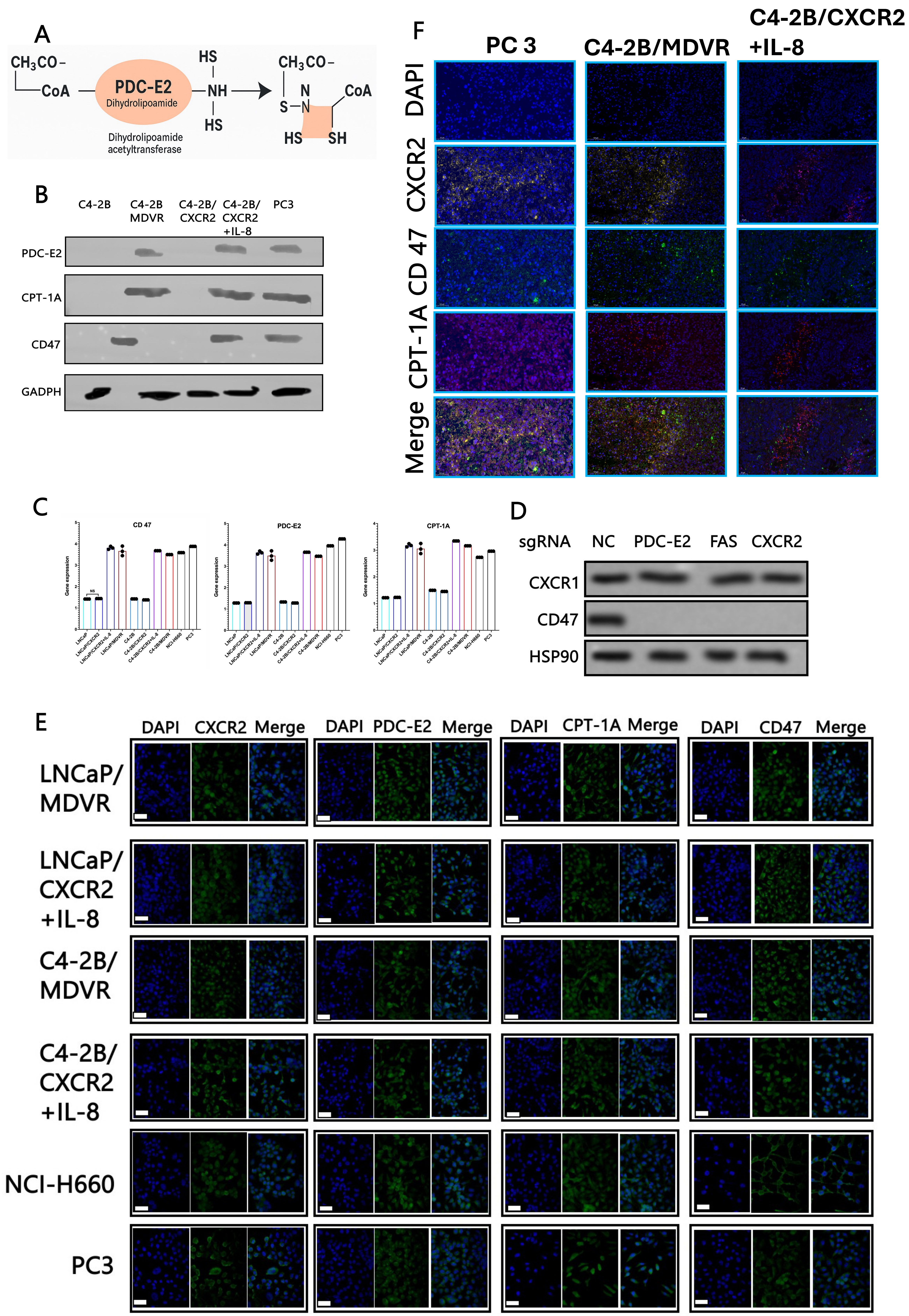

Supplemental Figure 4

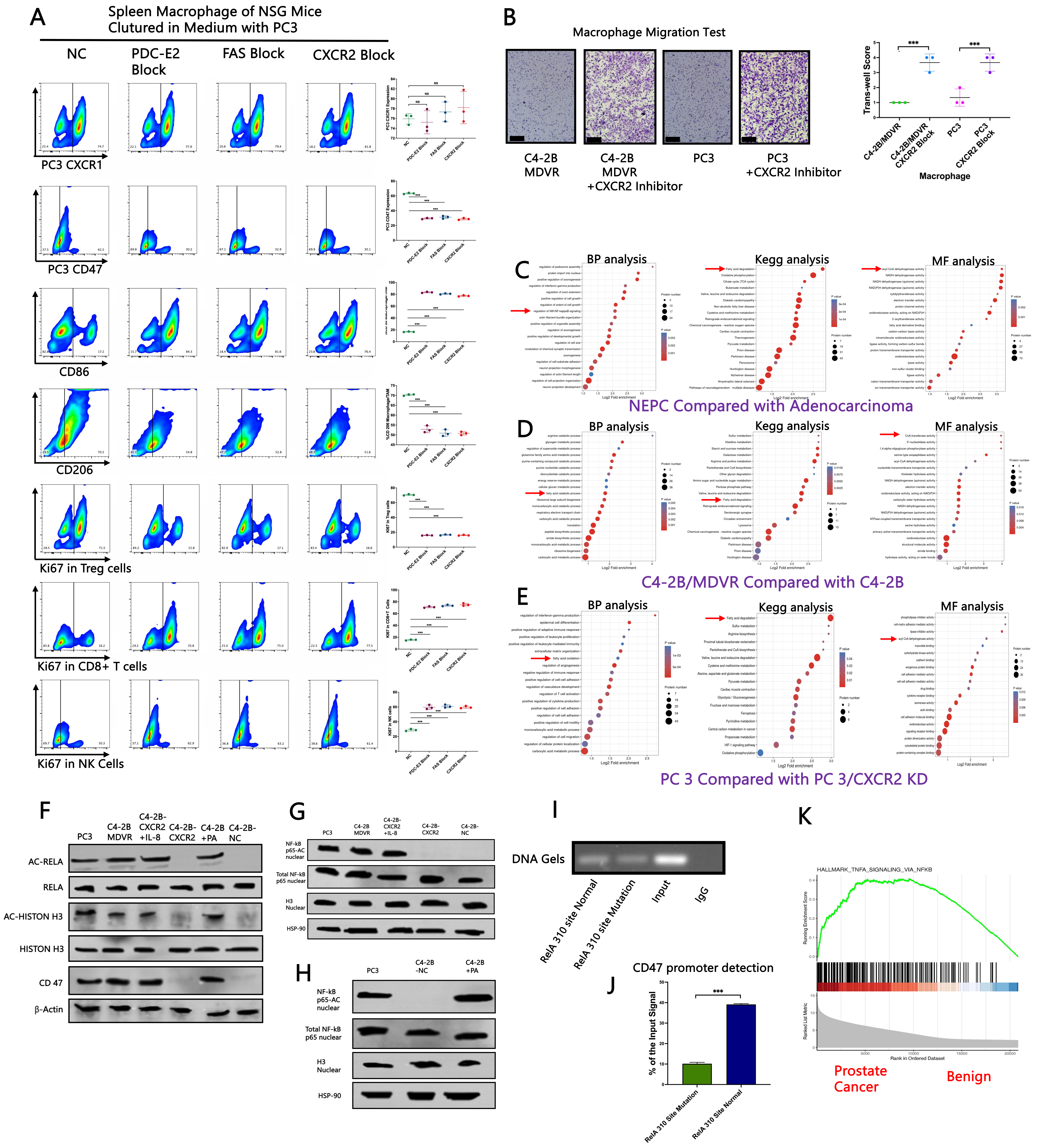

Supplemental Figure 5

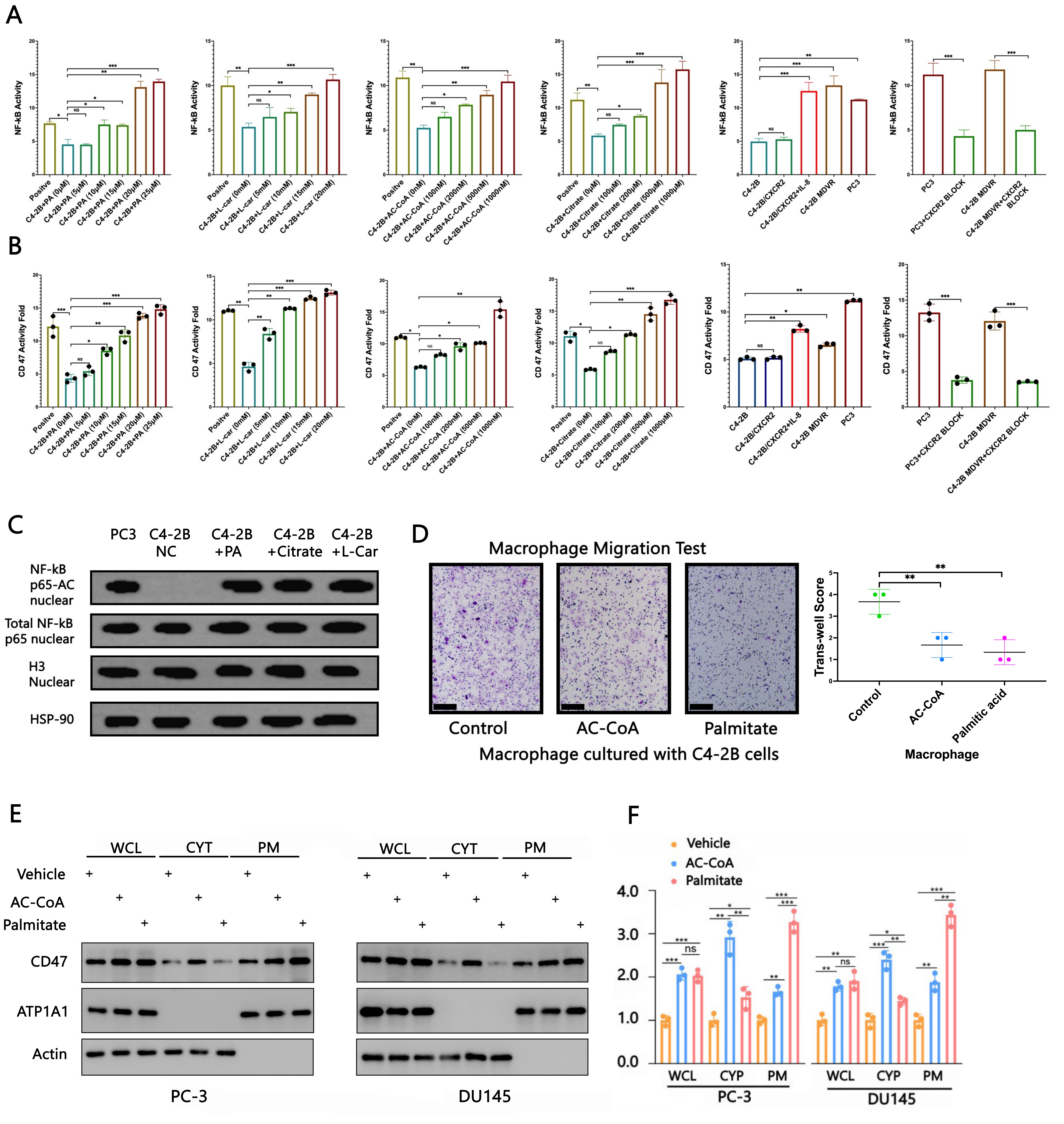

Supplemental Figure 6

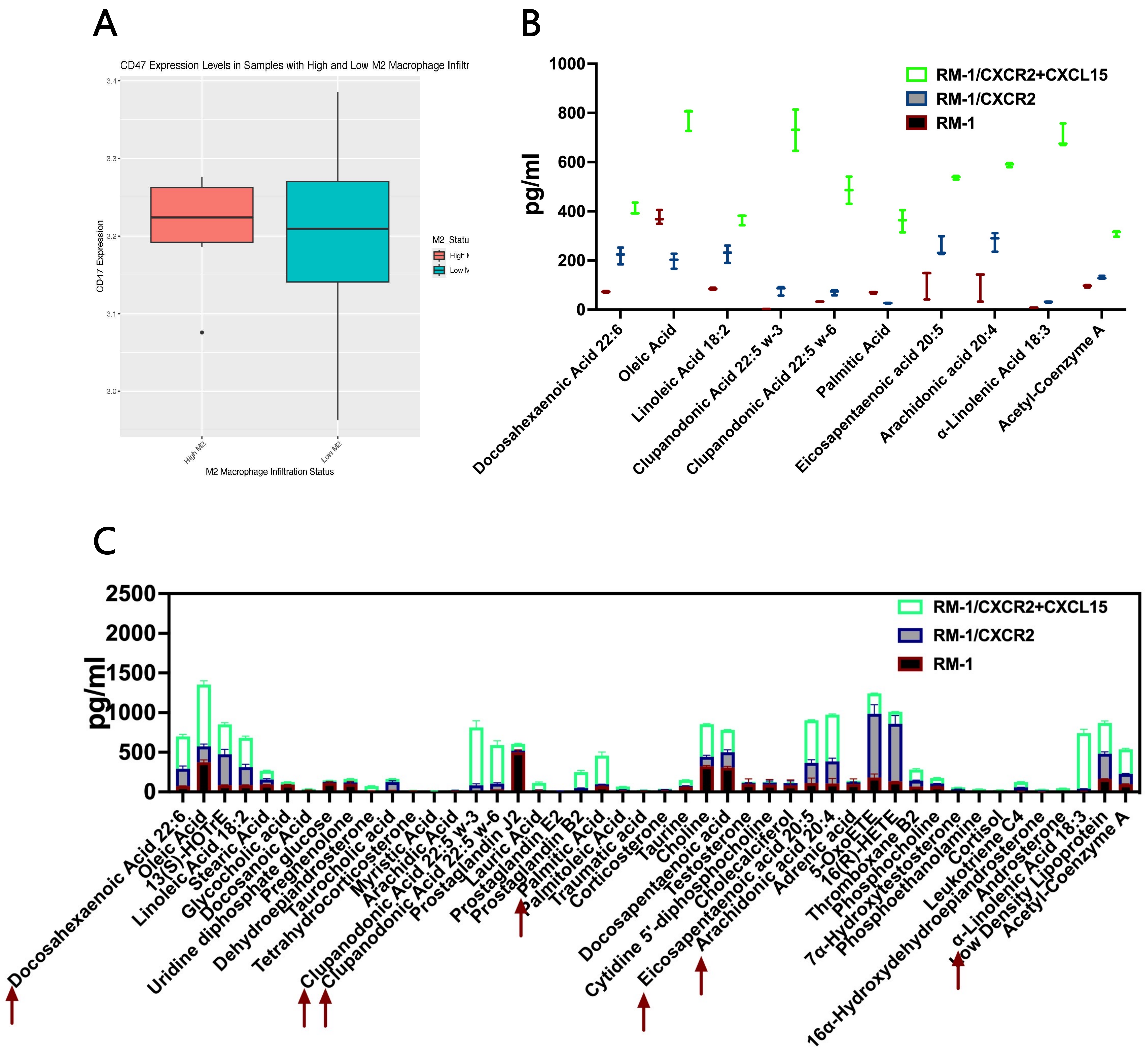

Supplemental Figure 7

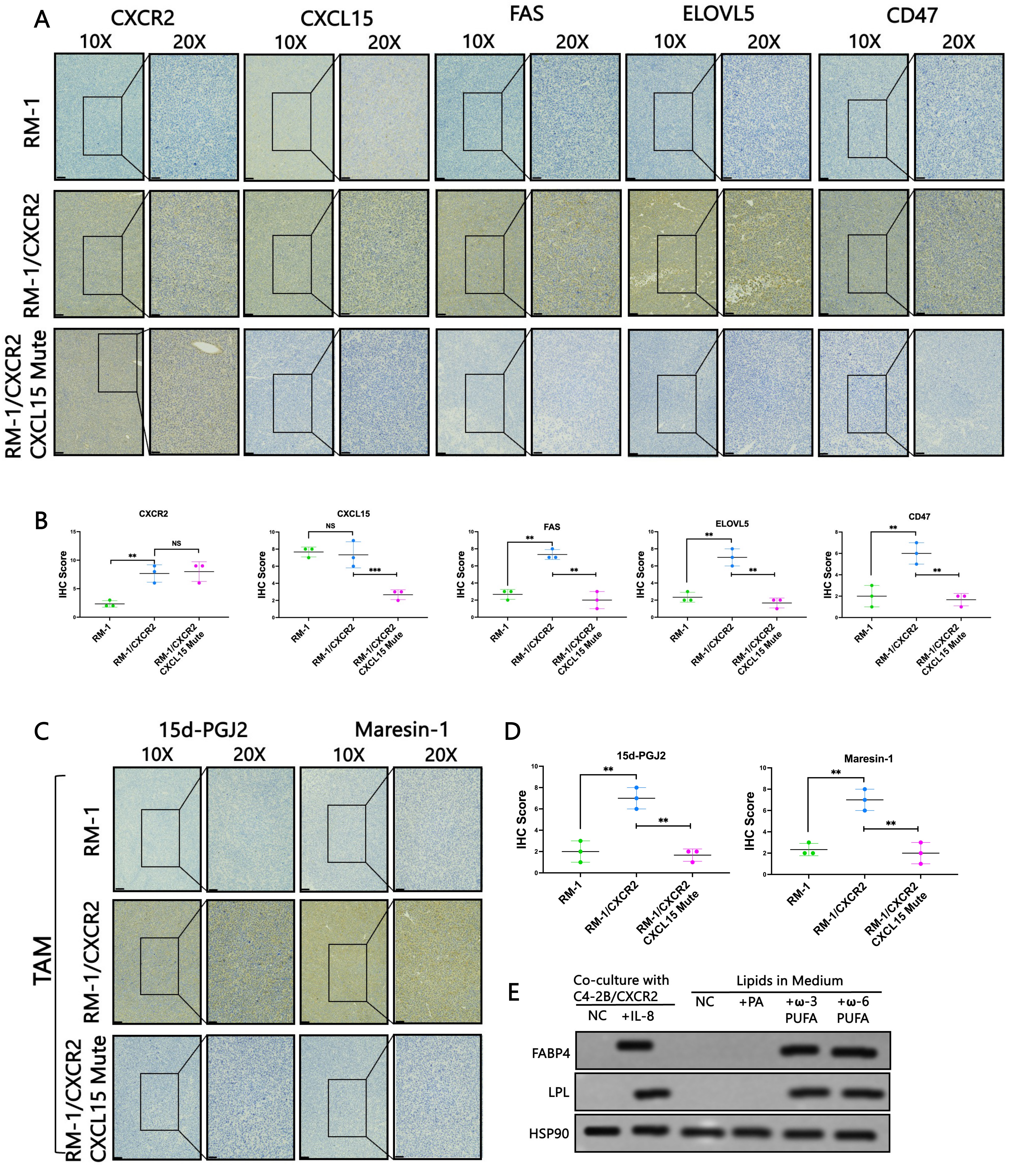

Supplemental Figure 8

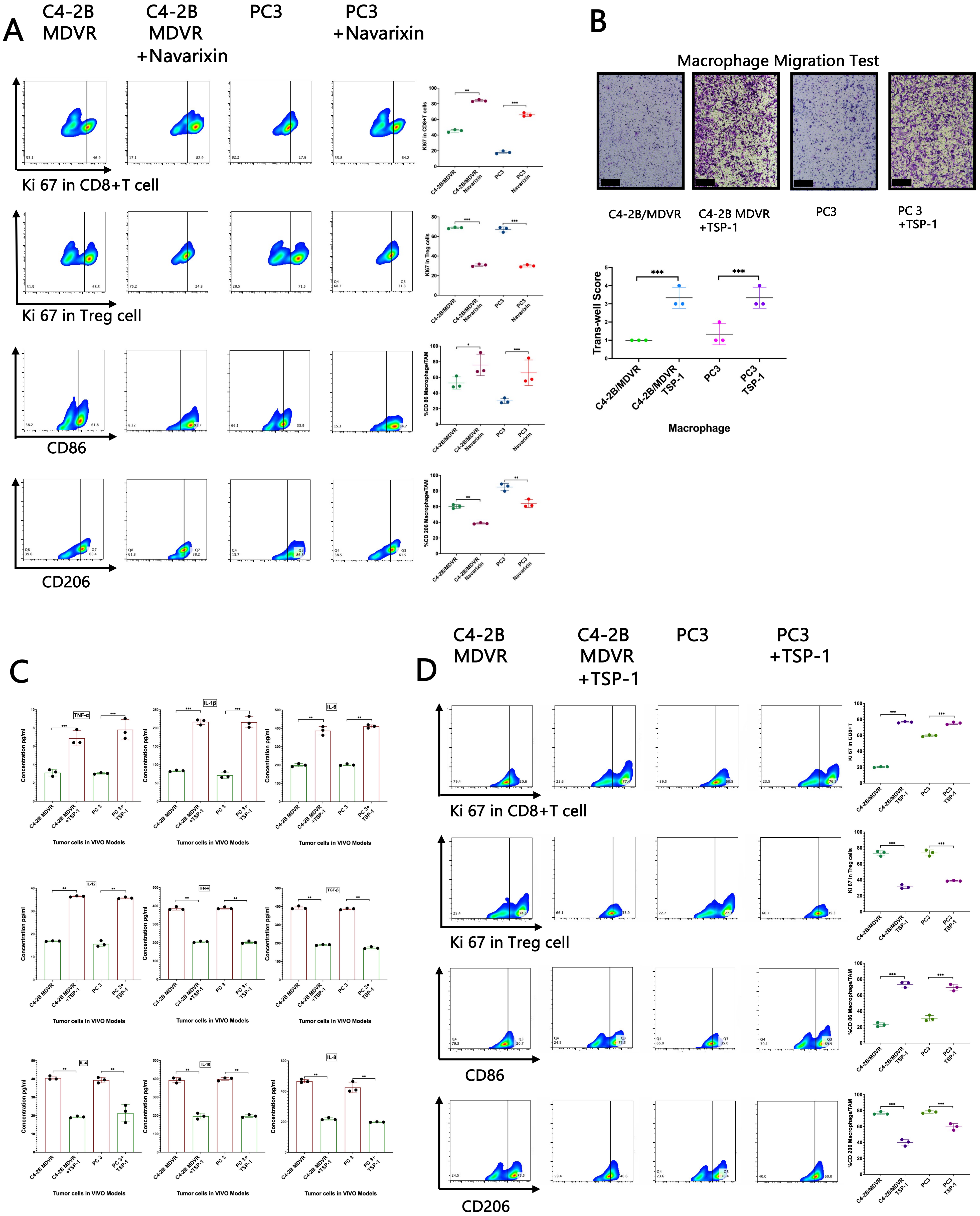

Supplemental Figure 9
